# Genes associated with genotype-specific DNA methylation in squamous cell carcinoma as candidate drug targets

**DOI:** 10.1186/1752-0509-8-S1-S4

**Published:** 2014-01-24

**Authors:** Ryoichi Kinoshita, Mitsuo Iwadate, Hideaki Umeyama, Y-h Taguchi

**Affiliations:** 1 Department of Physics, Chuo University, 1-13-27 Kasuga, Bunkyo-ku, Tokyo 112-8551, Japan; 2 Department of Biological Science, Chuo University, 1-13-27 Kasuga, Bunkyo-ku, Tokyo 112-8551, Japan

**Keywords:** genotype, DNA methylation, principal component analysis, protein tertiary structure, cancer, *in silico *drug discovery, gene selection

## Abstract

**Background:**

Aberrant DNA methylation is often associated with cancers. Thus, screening genes with cancer-associated aberrant DNA methylation is a useful method to identify candidate cancer-causing genes. Aberrant DNA methylation is also genotype dependent. Thus, the selection of genes with genotype-specific aberrant DNA methylation in cancers is potentially important for tailor-made medicine. The selected genes are important candidate drug targets.

**Results:**

The recently proposed principal component analysis based selection of genes with aberrant DNA methylation was applied to genotype and DNA methylation patterns in squamous cell carcinoma measured using single nucleotide polymorphism (SNP) arrays. SNPs that are frequently found in cancers are usually highly methylated, and the genes that were selected using this method were reported previously to be related to cancers. Thus, genes with genotype-specific DNA methylation patterns will be good therapeutic candidates. The tertiary structures of the proteins encoded by the selected genes were successfully inferred using two profile-based protein structure servers, FAMS and Phyre2. Candidate drugs for three of these proteins, tyrosine kinase receptor (ALK), EGLN3 protein, and NUAK family SNF1-like kinase 1 (NUAK1), were identified by ChooseLD.

**Conclusions:**

We detected genes with genotype-specific DNA methylation in squamous cell carcinoma that are candidate drug targets. Using *in silico *drug discovery, we successfully identified several candidate drugs for the ALK, EGLN3 and NUAK1 genes that displayed genotype-specific DNA methylation.

## Background

Promoter methylation is widely recognized as an important factor that regulates gene expression, especially in cancers [1,2]. Many genes with tumor-specific methylated promoters have been identified. For example, the promoters of the PAK3, NISCH, KIF1A, and OGDHL genes are specifically methylated in several cancers, including breast, esophagus, lung, pancreas, colon, prostate, gastric, cervix, thyroid, kidney, head and neck, ovary, and bladder cancers [3]. Because genes with methylated promoters are believed to be suppressive, genes with tumor-specific hypermethylated promoters were assumed to be tumor suppressors. Similarly, genes with tumor-specific hypomethylated promoters were supposed to be oncogenic (i.e., expressed in tumors) and potential oncogene targets. Identification of promoter methylation in cancer genes is important in helping to find critical genes that can cause cancer formation.

Genotype, on the other hand, is another critical factor that can affect cancer formation [3]. Many genotypes are known to be associated with cancers. Currently, there are no established mechanisms that can relate gene mutations to cancer formation. For example, a cancer-specific single nucleotide polymorphism (SNP) is often associated with specific cancers [4], but this SNP is located in an intron of the gene. It is still unclear how intronic SNPs affect gene expression. Typically, cancer-associated genotypes work solely as biomarkers.

Despite of the known importance of DNA methylation and genotype on cancer formation, how DNA methylation and genotype cooperatively mediate cancer formation has rarely been discussed. An exception is the recent association study reported by Scherf et al. [5] who found that genotype-specific promoter DNA methylation of the oncogene CHRNB4 was related to lung cancer. Opavsky et al. [6] also found that the P53, E2f2 and Pten genes in a mouse model of lymphoma were methylated in a genotype-specific manner. Thus, genotype and DNA methylation may contribute cooperatively to cancer formation in many other cancers.

In this paper, we sought to detect genotype-specific DNA methylation in esophageal squamous cell carcinoma (ESCC). Many previous studies have reported ESCC-specific genotypes. For example, Abnet et al. [7] found that genotypic variants at position 2q33 on the human chromosome were related to risk of ESCC. Maeng et al. [8] found that phosphoinositide-3-kinase and BRAF mutations were associated with metastatic ESCC and Wang et al. [9] found that ESCC was related to polymorphisms in ALDH2 and ADH1B in Chinese females. Thus, genotype-specific DNA methylation is expected to exist widely in ESCC. In this study, we used two publicly available distinct SNP microarray data sets to identify genotype-specific DNA methylation in ESCC.

## Methods

### DNA methylation profiles and genotypes

DNA methylation profiles and genotypes of blood, and normal and tumor tissues for 30 patients from two SNP arrays, Nsp and Sty, were downloaded from the Gene Expression Omnibus (GEO) at the National Center for Biotechnology Information [GEO:GSE20123] [10]. A total of 90 samples for each of the DNA methylation and genotypes were obtained. The normalized data were used without further preprocessing.

### Principal component analysis of DNA methylation profiles and genotypes

The downloaded samples were analyzed by principal component analysis (PCA) after substituting a zero for missing values. Principal components (PCs) that exhibited differences between the blood, normal tissue, and tumor tissue samples were selected for further analysis.

### Selection of SNPs (probes) based on PCs and a *t*-test

The top *N *outliers among the PCs were selected as described previously [11]. The DNA methylation profiles and genotypes were investigated by three pairwise one-sided *t*-test comparisons: normal tissue vs tumor, blood vs tumor, and blood vs normal tissue. Then, the SNPs (probes) with significant *P-*values (*P <*0.05, adjusted by the Bonferroni correction [12]) for all three pairwise comparisons were considered to be genes that displayed significant differences between all three cell types. Finally, genes that are selected in common for DNA methylation and genotypes were picked up for further analysis.

### Gene annotation using the Gendoo server

Gene annotation was performed with Gendoo (gene, disease features ontology-based overview system) [13,14]. The RefSeq mRNA IDs for the selected genes were extracted from GEO and transformed to the gene symbols. The gene symbols were then uploaded to the Gendoo server and diseases that were associated with gene symbols were listed with their *P-*values, which indicated the significance of the associations.

### Feature selection based on correlation coefficients

Suppose *x_ij _*is the microarray measurement for the *i*th probe (SNP) at the *j*th sample and *y_j _*depends on the class to which the *j*th sample belongs, then

$$y_{i}=\left\{\begin{array}{cc} 1 & \left(j\in \text{blood}\right)\\ 2 & \left(j\in \text{normal tissue}\right).\\ 3 & \left(j\in \text{tumor}\right) \end{array}\right)$$

The Pearson and Spearman correlation coefficient for the *i*th probe (SNP) was then computed between *x_ij _*and *y_j_*. Finally, the 300 probes (SNPs) with the largest correlation coefficients were selected.

### Feature selection based on partial least squares

Partial least squares (PLS) provides a bilinear representation of data and PLS-based feature selection aims to select features that have the most weight to linear combinations [15]. For simplicity, we employed the PLS+MCLASS strategy [15], where PLS was applied directly to multiclass samples. This strategy is, at most, the third-best depending on the data set being tested (Other strategies include, for example, a voting strategy based on pairwise PLS applications [15]). However, because there are only three classes in our study, very little improvement can be expected even if the best strategy is employed, as shown previously [15].

### Stepwise feature selection

Stepwise feature selection was performed by adding/removing features iteratively, until the performance reached its maximum. In this study we performed stepwise variable selection using the stepclass function with the lda function as implemented in R [16].

### Lasso-based feature selection

Least absolute shrinkage and selection operator (Lasso) [17] is another frequently used feature extraction method. Lasso applies linear discriminant analysis with minimizing sum of regression coefficients. This results in the elimination of redundant features. To apply Lasso to our data set, we employed the LARS function implemented in R [16] by specifying the type=" lasso" option.

### *t*-test of the microarray measurements between genotype and DNA methylation

For the SNPs that were selected in common between genotype and DNA methylation, we used the one-sided *t*-test that rejects the null hypothesis that the microarray measurement of genotype is as large as the DNA methylation value in favor of the microarray measurement of genotype is more than the DNA methylation value. For random sampling, the same set of SNPs was used for the genotype and DNA methylation measurements.

### Protein tertiary structure prediction

To predict the tertiary structure of the proteins encoded by the selected genes we used the FAMS [18,19] and Phyre2 software [20,21].

### Screening drug candidate compounds from the DrugBank database

We downloaded 6583 compounds in smiles format from DrugBank [22,23]. The smiles format was transformed to three dimensional structures by Babel [24]. The structures of 6510 of the compounds were obtained. Tanimoto indices were computed between the individual compounds and ligands that bind to template proteins. Compounds with Tanimoto indices larger than the threshold values (0.25 for tyrosine kinase receptor (ALK), 0.20 for the other proteins) were selected as candidate drug compounds.

### Selection of template proteins and ligands

The template protein structures that we used for *in silico *drug discovery were selected as follows: first, each template must be used as a model protein for the ligand binding region of the target protein; second, the protein structures that ligands could bind to were selected as templates; and third, as many as possible of the ligands that could bind to several of the model proteins, including those not selected as templates, were selected and fitted to a template protein. These ligands were the "fingerprint" for drug discovery and were used for to compute the Tanimoto index.

### Docking simulation using ChooseLD

Docking between the screened compounds and template proteins was performed using ChooseLD [25]. The FPAScore [25] (minimization of free energy between each compound and template protein) were computed ten times for each compound. The compounds were ranked based on the best score among the ten values. Whole computations were performed independently three times and consistency between the three trials was evaluated.

### Estimation of coincident of highly ranked compounds between three independent trials

Suppose that $r_{i}^{\left(n\right)}$, (*i *= 1, *..*., *N_c_*, *n *= 1, 2, 3) is the descending rank order of the FPAScore attributed to the *i*th compound at the *n*th trial, where *Nc *is the total number of compounds considered, then,

$$S^{\left(n\right)}\left(k\right)=\left\{i|r_{i}^{\left(n\right)}\leq k\right\}$$

is the set of *k *highly ranked compounds at the *n*th trial. Then, the expected number of compounds selected in common up to the *k*th rank, *n*_0_(*k*), is computed when there are no correlations between the $r_{i}^{\left(n\right)}\text{s}$. Because the probability that *S*^(2)^(*k*) includes compounds in *S*^(1)^(*k*) is *k/Nc*, *S*^(2)^(*k*) is expected to include *k*^2^*/Nc *compounds that exist in *S*^(1)^(*k*). Thus, the number of unique compounds in *S*^(1)^(*k*) and *S*^(2)^(*k*) is expected to be

$$k+k-\frac{k^{2}}{N_{c}}=k\left(2-\frac{k}{N_{c}}\right)$$

and the probability that *S*^(3)^(*k*) includes compounds in either *S*^(1)^(*k*) or *S*^(2)^(*k*) is

$$\frac{k}{N_{c}}\left(2-\frac{k}{N_{c}}\right)$$

Thus, *S*^(3)^(*k*) is expected to include

$$\frac{k^{2}}{N_{c}}\left(2-\frac{k}{N_{c}}\right)$$

compounds that exist in either *S*^(1)^(*k*) or *S*^(2)^(*k*). Finally, the total number of unique compounds in *S*^(1)^(*k*), *S*^(1)^(*k*), and *S*^(3)^(*k*) is expected to be

$$n_{0}\left(k\right)=k\left(2-\frac{k}{N_{c}}\right)+k-\frac{k^{2}}{N_{c}}\left(2-\frac{k}{N_{c}}\right)=k\left\{1+\left(1-\frac{k}{N_{c}}\right)\left(2-\frac{k}{N_{c}}\right)\right\}$$

When the number of highly ranked compounds selected in common between the three independent trials is much less than this number and is close to *k*, we can conclude that consistency between the three trials is high.

## Results

### Estimation of genotype-specific DNA methylation

There is no unique criterion that can estimate genotype-specific DNA methylation. Aberrant methylation itself can be estimated by various criteria; for example, using the ratio or the difference of mean values between normal and tumor tissues or using *P-*values obtained by a statistical test such as a *t*-test. Each of the criterion may give a different genotype-specific DNA methylation set of genes. In addition, some genotypes are either heavily demethylated or methylated in tumor tissue compared with normal tissue. If this genotype is very rare in the tumor tissue, it is clearly unreasonable to regard this genotype-specific DNA methylation as being the cause of the tumor. Ideally, to be sure that a particular genotype-specific DNA methylation could cause the tumor, the following conditions should be satisfied:

1. The genotype is specifically demethylated/methylated in the tumor tissue compared with other genotypes (strength of aberrant DNA methylation).

2. The genotype is abundant in the tumor tissue (abundance of aberrant DNA methylation).

The best balance between these two conditions is not easy to estimate, because there is no standard understanding about the kind of gene abnormalities that generally cause tumors. In this study, we used three kinds of samples: blood, normal and tumor tissues. This made the comparisons more difficult than a comparison between only normal and tumor tissues, because we are not sure if normal tissue is an expected intermediate between blood and tumor. To avoid uncertainties that this complicated situations might cause when estimating genotype-specific DNA methylation, we employed a recently proposed PCA-based unsupervised feature selection method [11]. This procedure does not require the user to select the criterion that is used to estimate genotype-specific DNA methylation. It is necessary simply to select the suitable PC by which the SNPs with genotype-specific DNA methylation are selected.

### Genotype-specific DNA methylation estimated using the Nsp microarray data

**Figure 1:**
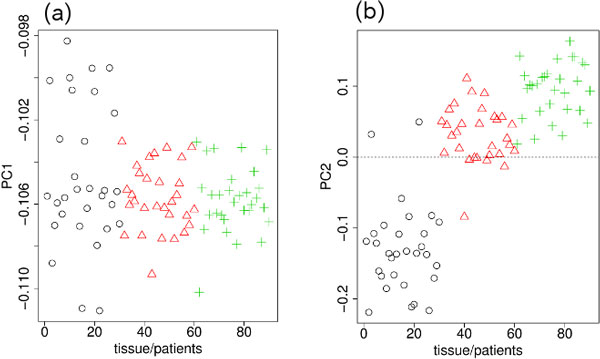
**PCs for genotypes measured by Nsp microarray**. (a) PC1 (81%). (b) PC2 (3%). Black circle, blood; red triangle, normal tissue; green cross, tumor tissue. The horizontal axes indicate the subjects and their samples. The order of the 30 subjects in the 1―30, 31 ―60, and 61―90 sections are the same; i.e., 1, 31, and 61 are samples from the same patient.

The PCs obtained when PCA was applied to the Nsp microarray measurements of genotype are shown in Figure 1. Although the first PC (PC1; Figure 1a) had the dominant contribution (80%), no significant differences between blood, and the normal and tumor tissues were seen. On the other hand, the second PC (PC2; Figure 1b) clearly distinguished between blood, and normal and tumor tissues. Therefore, we used PC2 to select probes (SNPs) that exhibited significant differences between the blood, and normal and tumor tissues. Because PC3 (not shown here) exhibited no significant differences between the blood, normal and tumor samples and had very little contribution, we did not use the third PC (PC3) to select SNPs.

**Figure 2:**
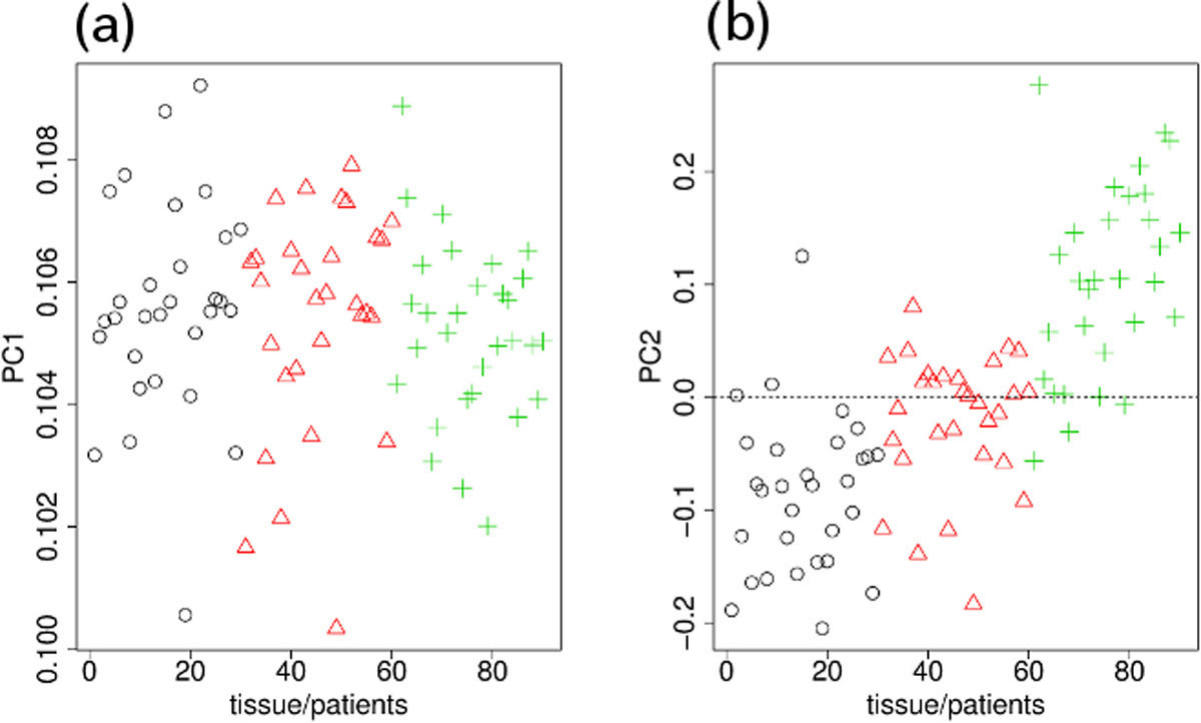
**PCs for DNA methylation measured by Nsp microarray**. (a) PC1 (80%). (b) PC2 (3%). Other notations are the same as those in Figure 1.

The PCs obtained when PCA was applied to the Nsp microarray measurements of DNA methylation are shown in Figure 2. PC2 (Figure 2b) was again the PC that clearly distinguished between blood, and normal and tumor tissues. PC2 was, therefore, used to select the SNPs that exhibited significant differences between the three samples.

**Figure 3:**
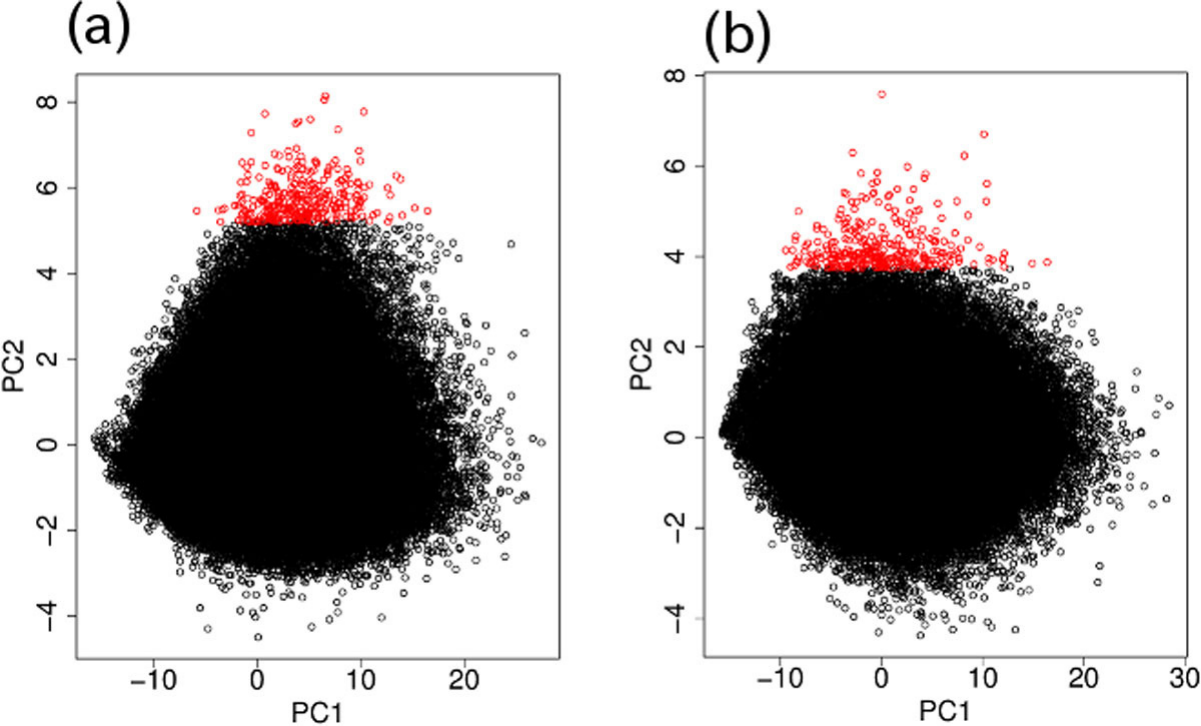
**Two dimensional embedding of SNPs with PC1 and PC2 for the Nsp microarray measurements**. (a) Genotype (Figure 1). (b) DNA methylation (Figure 2). The top 300 outliers are shown in red.

The two dimensional (PC1 and PC2) embedding of SNPs (probes) for DNA methylation and genotype are shown in Figure 3. Because PC2 showed significant differences between the blood, and normal tissues and tumor tissues, we selected the 300 topmost outliers along the PC2 axis for both DNA methylation and genotype. To see if genotype-specific methylated SNPs were selected correctly, we filtered the selected SNPs based on the following criteria:

1. Intersection between top *N *outliers between DNA methylation and genotype.

2. All three associated *P-*values adjusted by the BH criterion [26] are less than 0.05, when three pairwise one-sided *t*-tests (tumor tissue vs normal tissue, normal tissue vs blood, tumor tissue vs blood) are applied.

A total of 68 SNPs were selected in common from the top 300 outliers between genotype and DNA methylation after applying the first criterion. Because there were more than 250,000 SNPs on the Nsp microarray, the *P*-value for 68 SNPs being selected in common from 300 is less than 1 × 10^−16^. The topmost 5, 10, 20, 27, 42 and 59 selected SNPs from within the top *N *(=50, 100, 150, 200, 250, and 300) outliers, respectively, after applying the *P*-value filtering (the second criterion) are listed in Table 1. More detailed annotations for selected SNPs and their associated genes are available in Additional file 1.

**Table 1:** SNPs selected for DNA methylation and genotype measured by the Nsp microarray.

Rank	SNPs			
50	SNP_A-1825620	SNP_A-2213037	SNP_A-2145008	SNP_A-2309865
	SNP_A-4233167			

100	SNP_A-2172952	SNP_A-2234716	SNP_A-1984943	SNP_A-2121000
	SNP_A-2085071			

150	SNP_A-2040111	SNP_A-4195285	SNP_A-4199352	SNP_A-2089983
	SNP_A-1944699	SNP_A-1988914	SNP_A-1834529	SNP_A-1950742
	SNP_A-2105346	SNP_A-4235277		

200	SNP_A-4229534	SNP_A-4226834	SNP_A-4196078	SNP_A-2199615
	SNP_A-1919825	SNP_A-2276203	SNP_A-1961374	

250	SNP_A-1989613	SNP_A-1845324	SNP_A-1880907	SNP_A-2142865
	SNP_A-2124767	SNP_A-1810962	SNP_A-4193660	SNP_A-1852621
	SNP_A-1961109	SNP_A-4212314	SNP_A-2042678	SNP_A-1886593
	SNP_A-1980533	SNP_A-2143521	SNP_A-2088571	

300	SNP_A-2043441	SNP_A-2287632	SNP_A-2056366	SNP_A-2185001
	SNP_A-1910539	SNP_A-4213049	SNP_A-2007288	SNP_A-4228665
	SNP_A-4236336	SNP_A-2063926	SNP_A-1911642	SNP_A-1950919
	SNP_A-2053247	SNP_A-4197286	SNP_A-4204073	SNP_A-2221049
	SNP_A-2065785			

The 59 selected SNPs (probes) that exhibited significant differences between blood, and normal and tumor tissues, within the top N (= 50, 100, 150, 200, 250, and 300) outliers for both DNA methylation and genotype. Top 300 outliers are shown in red in Fig. 3. For detailed annotations, including associated genes, of the selected SNPs, see Additional file 1 (sheet name Nsp).

### Genotype-specific DNA methylation estimated using the Sty microarray data

**Figure 4:**
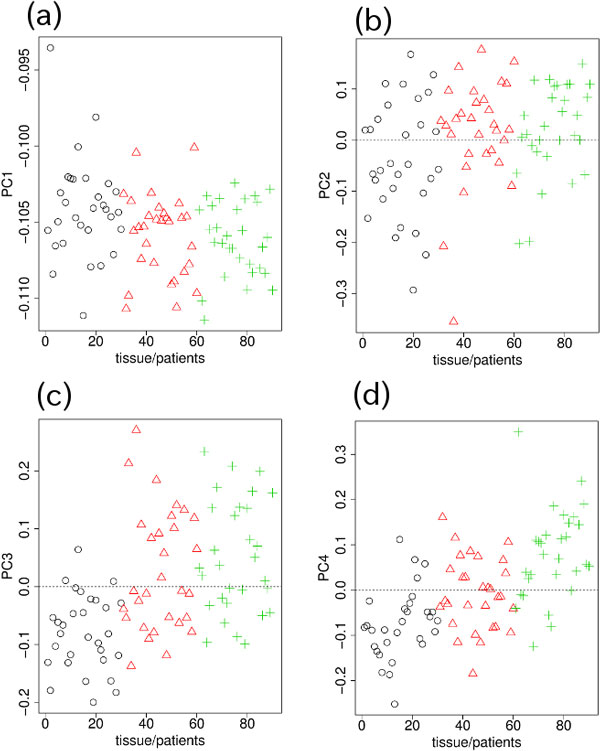
**PCs for genotype measured by Sty microarray**.(a) PC1 (81%). (b) PC2 (3%). (c) PC3 (2%). (d) PC4 (1%). Other notations are the same as those in Fig. 1.

The PCs obtained when PCA was applied to the Sty microarray measurements of genotype are shown in Figure 4. Although PC1 (Figure 4a) had the dominant contribution of 81%, no significant differences between blood, normal and tumor tissues were observed. PC2 (Figure 4b) had very little contribution and also exhibited no significant differences between the three samples. On the other hand, both PC3 (Figure 4c) and the fourth PC (PC4; Figure 4d) clearly distinguished between blood, normal and tumor tissues. Because the PC3 and PC4 results were similar, at this stage we did not decide which of them was the more suitable PC to use to select SNPs that exhibited significant differences between blood, normal and tumor tissues.

**Figure 5:**
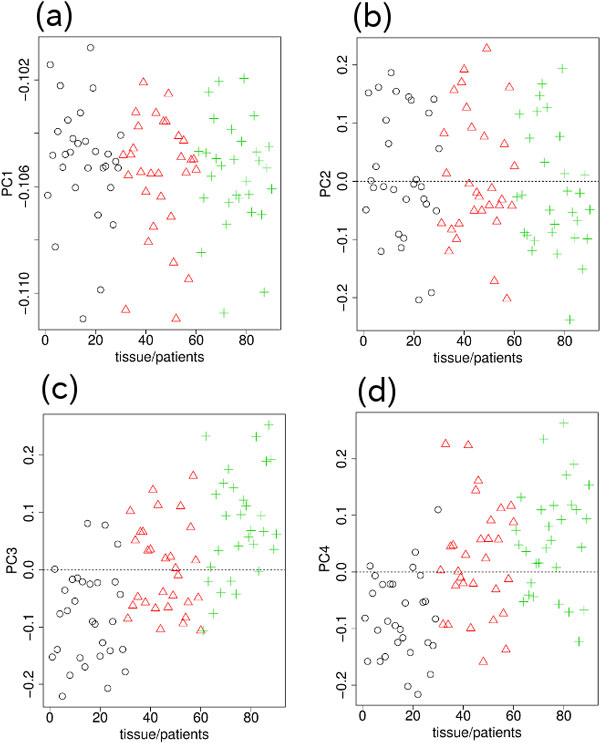
**PCs for DNA methylation measured by Sty microarray**. (a) PC1 (83%). (b) PC2 (2%). (c) PC3 (1%). (d) PC4 (1%). Other notations are the same as those in Fig. 1.

The PCs obtained when PCA was applied to the Sty microarray measurements of DNA methylation are shown in Figure 5. PC3 (Figure 5c) and PC4 (Figure 5d) were again the PCs that clearly distinguished between blood, normal and tumor tissues. PC1 (Figure 5a) and PC2 (Figure 5b) did not exhibit strong significant differences.

Because, unlike in the case using the Nsp microarray data, we could not uniquely select a pair of PCs to use to select the SNPs that exhibited the most significant differences between the blood, normal and tumor tissues, we tried various PC combinations for the genotype and DNA methylation measurements. We found that the best combinations were

1. PC4 for genotype (Figure 4d) and PC3 for DNA methylation (Figure 5c).

2. PC3 for genotype (Figure 4c) and PC4 for DNA methylation (Figure 5d).

**Figure 6:**
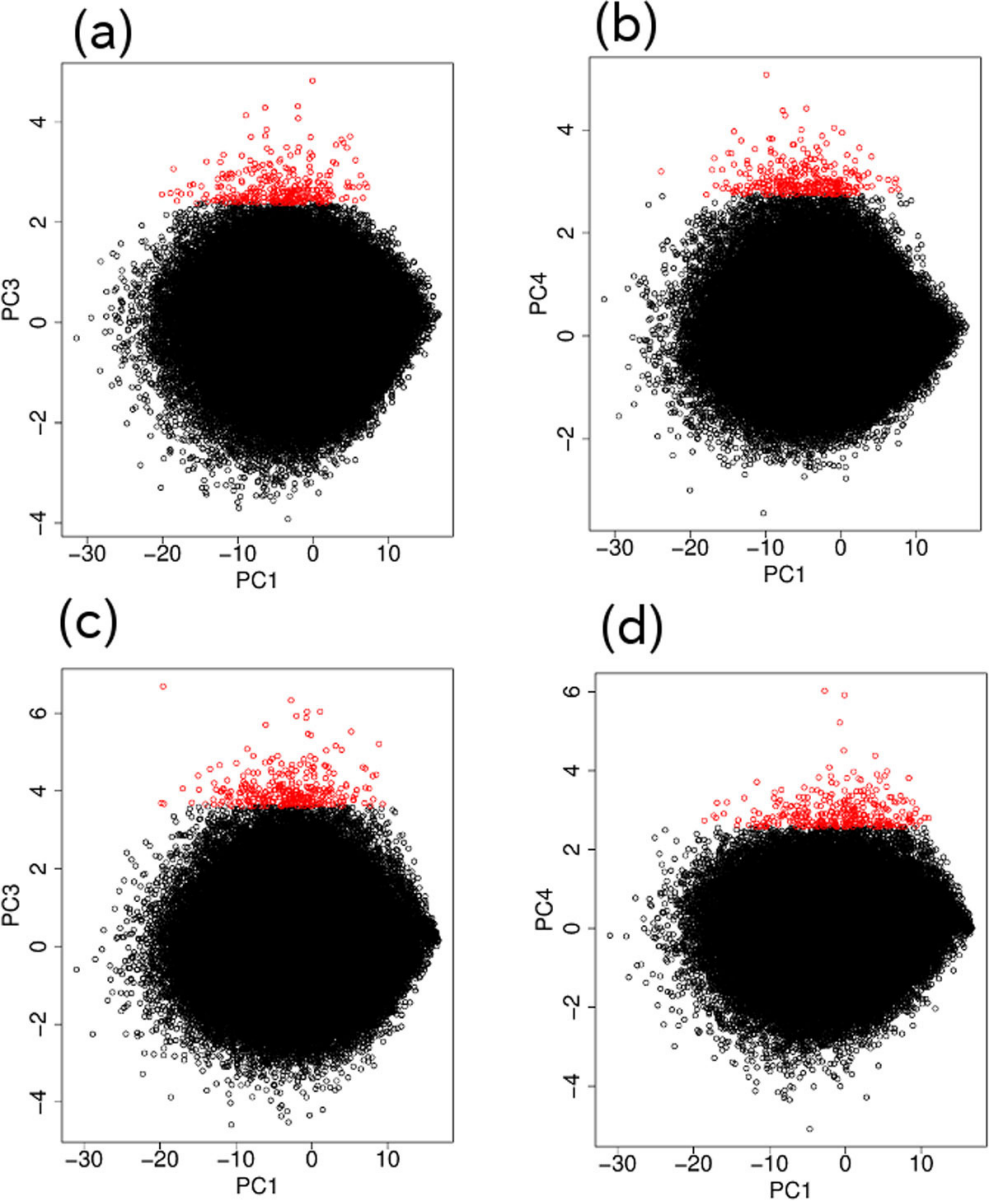
**Two dimensional embedding of SNPs for the Sty microarray measurements**. For genotype (a) with PC1 (Figure 4a) and PC3 (Figure 4c) and (b) with PC1 (Figure 4a) and PC4 (Figure 4d). For DNA methylation (c) with PC1 (Figure 5a) and PC3 (Figure 5c) and (d) with PC1 (Figure 5a) and PC4 (Figure 5d). Top 300 outliers are shown in red.

The two dimensional embedding of SNPs (probes) for DNA methylation and genotypes for these two combinations of genotype and DNA methylation PCs are shown in Figure 6. SNPs (probes) that exhibited differences between the three samples, in common for both DNA methylation and genotype, were selected using the criteria described in the previous section. For the combination of PC4 for genotype (Figure 6b) and the PC3 for DNA methylation (Figure 6c), a total of 81 SNPs were selected in common within the 300 topmost outliers between DNA methylation and genotype after applying the first criterion. Similarly, for the combination of PC3 for genotype (Figure 6a) and PC4 for DNA methylation (Figure 6d), a total of 50 SNPs were selected in common within the 300 topmost outliers between DNA methylation and genotype after applying the first criterion. Because there were more than 230,000 SNPs on the Sty microarray, the *P-*values for 81 or 50 SNPs being selected in common from 300 are less than 1 × 10^−16^. The topmost 6 (4), 10 (15), 13 (21), 14 (28), 19 (34), and 22 (37) SNPs from the top *N *(=50, 100, 150, 200, 250, and 300) outliers, respectively, were selected after applying the *P*-value filtering for the first (second) combination of PCs are listed in Table 2 (3). More detailed annotations for selected SNPs and their associated genes are available in Additional file 1.

**Table 2:** SNPs measured by the Sty microarray using PC4 for genotype and PC3 for DNA methylation.

Rank	SNPs			
50	SNP_A-2176803 SNP_A-2114077	SNP_A-4286712 SNP_A-4277414	SNP_A-4276813	SNP_A-2134351

100	SNP_A-4252327	SNP_A-4271493	SNP_A-4261117	SNP_A-1955805

150	SNP_A-2278684	SNP_A-2159288	SNP_A-1798268	

200	SNP_A-1975466			

250	SNP_A-2221439 SNP_A-2198500	SNP_A-2175811	SNP_A-4259136	SNP_A-2186260

300	SNP_A-4247667	SNP_A-4296608	SNP_A-4302067	

The 22 selected SNPs (probes) that exhibited significant difference between blood, and normal and tumor tissues, within the top *N *(= 50, 100, 150, 200, 250, and 300) outliers for both DNA methylation and genotype. Top 300 outliers are shown in red in Figures 6b and 6c. For detailed annotations, including associated genes, of the selected SNPs, see Additional file 1 (sheet name Sty1).

### Estimation of optimal *N*

We did not know what is the optimal *N*, the number of selected SNPs with aberrant DNA methylation, to use in this study. The results in Tables 1, 2, and 3 indicate that increasing *N *helps in selecting a large enough number of SNPs that pass the selection criteria. Larger *N*s possibly give more plausible SNPs with genotype-specific DNA methylation. We used *N *= 300 mainly because, when a large number of SNPs are selected at this stage, in the following stages, it is difficult to screen the SNPs and to predict the tertiary structure of the proteins associated with the SNPs. A rigorous estimation of the optimal *N *is a problem for future studies.

**Table 3:** SNPs measured by the Sty microarray using PC3 for genotype and PC4 for DNA methylation.

Rank	SNPs			
50	SNP_A-1879798	SNP_A-4261939	SNP_A-4288122	SNP_A-1906431

100	SNP_A-1781703 SNP_A-4254588 SNP_A-2291931	SNP_A-4257840 SNP_A-1827527 SNP_A-1893004	SNP_A-1990707 SNP_A-2272342 SNP_A-1859078	SNP_A-2092003 SNP_A-2264321

150	SNP_A-4292799 SNP_A-2188211	SNP_A-4285002 SNP_A-4263960	SNP_A-2295075	SNP_A-2176803

200	SNP_A-4275514 SNP_A-4279597	SNP_A-4242077 SNP_A-4288260	SNP_A-2073412 SNP_A-4300538	SNP_A-1793920

250	SNP_A-4258351 SNP_A-4269967	SNP_A-2207678 SNP_A-1811356	SNP_A-4277760	SNP_A-1834280

300	SNP_A-4258451	SNP_A-4302014	SNP_A-4293935	

The 37 selected SNPs (probes) that exhibited significant difference between blood, and normal and tumor tissues, within the top *N *(= 50, 100, 150, 200, 250, and 300) outliers for both DNA methylation and genotype. Top 300 outliers are shown in red in Figures 6a and 6d. For detailed annotations, including associated genes, of the selected SNPs, see Additional file 1 (sheet name Sty2).

### Comparison with other methods

To our knowledge, no feature selection methods that are applicable to three classes of data set without the need for preknowledge about the internal ranking between the classes are currently available. Although our method requires the manual selection of the PCs used for feature selection, no pre-knowledge about the ranking between classes is needed and how the classes should be ranked is quite clear from the PCs (Figures 1, 2, 4, and 5). Thus, there are no other methods that can be compared with our methods.

However, because we now know that the rank between the classes is blood *<*normal tissue *<*tumor tissue, we have applied other methods that require this pre-knowledge.

Table 4 shows the selection results obtained using our method and several other methods (see Methods). LARS with the type="lasso" setting option could not be executed because the memory requirements were too large and stepclass did not converge within the executable time period. These problems were because the more than 200,000 probes (SNPs) in each of the the two microarrays were too many for the available memory or timeframe.

**Table 4:** Comparison of our method with other feature selection methods.

Method	Nsp	Sty		
		(Sty1		Sty2)
Present	68	81		50
Pearson	49		14	
Spearman	39		18	
PLS	7		13	
Stepclass	--		--	
lasso	--		--	

The number of SNPs selected in common between the top-ranked 300 SNPs in genotype and DNA methylationusing various methods. Present, this study; Pearson, Pearson correlation coefficients based method; Spearman, Spearman correlation coefficients based method; PLS, partial least squares based method; Stepclass, stepclass (R function that executes iterative feature selection) based method; and lasso: Lasso based method. Nsp and Sty are the microarray data sets used in the study. Sty1 and Sty2 correspond to the PC4 for genotype (Figures 4d and 6b)/PC3 for DNA methylation (Figures 5c and 6c) and the PC3 for genotype (Figures 4c and 6a)/PC4 for DNA methylation (Figures 5d and 6d) combinations of PCs.

The Pearson correlation-based, Spearman correlation-based, and PLS-based feature selection methods successfully selected the 300 topmost SNPs for genotype and DNA methylation. However, the number of SNPs selected in common between genotype and DNA methylation was smaller than the numbers selected the present study (Table 4). Thus, our method clearly outperforms the other methods in selecting the genes in common between genotype and DNA methylation.

## Discussion

### Properties of the selected SNPs

Almost all selected SNPs were located outside protein cording regions of the genes (see Additional file 1). The only exceptions were SNP_A-4242077 (associated with PIWIL1), SNP_A-4288260 (associated with PIGO), and SNP_A-1988914(associated with TARBP1). Thus, the majority of the SNPs are presumably related to the regulation of gene expression. The SNPs that were not located in protein coding regions were located in the promoters (identified as "upstream" in additional file 1), and also in introns and in the downstream regions of genes. Thus, the effect of genotype-specific DNA methylation on gene expression is not straightforward.

In addition, some of the selected SNPs have not been reported in Chinese populations, although all patients in the microarray data sets that we used in this study were Chinese. This finding indicates that we have correctly selected mutation that may cause cancer formation.

### Screening of cancer-related genes

To determine if the selected SNPs are biologically related to cancers, the genes containing the SNPs were annotated using Gendoo [13,14]. The RefSeq mRNA IDs of the genes were extracted from GEO and mapped to gene symbols (Additional file 2). The gene symbols were uploaded to the Gendoo server and the diseases that were reported to be associated with each of the gene symbols were listed (see Additional file 3). We found that 86 of the 155 genes listed in Additional file 2 were associated with at least one cancer-related disease. In addition, we performed a literature search to find papers that reported the relationship between any of the 86 selected genes and cancers, because the Gendoo server annotation is based on automated text-mining and may include some misinterpretations. We found that most of 86 genes were mentioned in at least one published paper that described their relationship with cancer (see Additional file 4). Thus, we confirmed that more than half (86) the 155 genes screened by our method were cancer-related genes. In particular, twelve genes (CCND1, CCNL1, CKAP4, CRABP1, FGF3, GRHL2, MYEOV, PKP4, RAP2B, RPL14, SMAD3, ZNF639) were associated with "Carcinoma, Squamous Cell" and eleven genes (CCND1, CKAP4, CRABP1, EVI1, FGF3, MYEOV, PKP4, RPL14, SMAD3,TMEM16A,ZNf639) were associated with "Esophageal Neoplasms". Among them, nine genes are associated with both. Because this study used data sets for ESCC (esophageal squamous cell carcinoma), this association is reasonable and demonstrates the reliability of our method.

### Genes with genotype-specific DNA methylation are less methylated than expected

**Figure 7:**
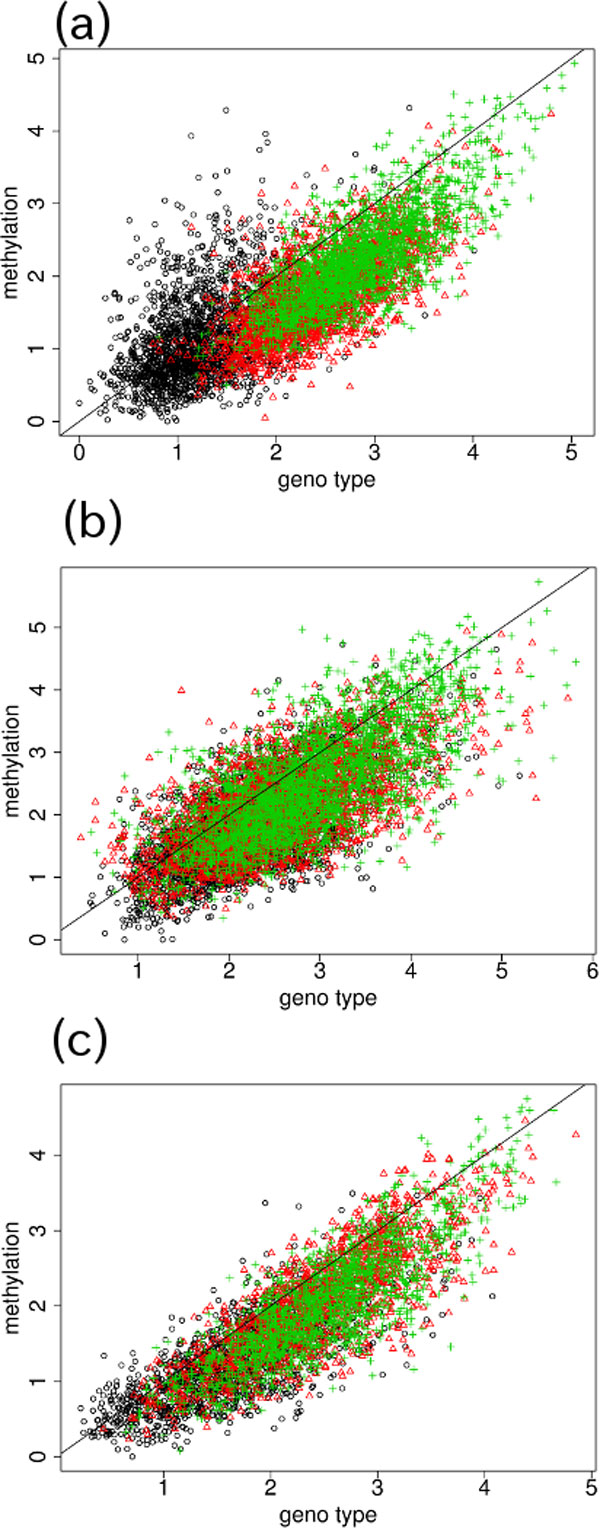
**Comparison of microarray measurements between genotype and DNA methylation.** Comparison of (a) 68 SNPs selected with the Nsp microarray data set and (b) 81 SNPs selected with the Sty microarray data set using PC4 for genotype (Figures 4d and 6b) and PC3 for DNA methylation (Figures 5c and 6c). Comparison of (c) 50 SNPs selected with the Sty microarray using PC3 for genotype (Figures 4c and 6a) and PC4 for DNA methylation (Figures 5d and 6d). Black circle, blood; red triangle, normal tissue; green cross, tumor tissue. Solid lines indicate the boundary where the microarray measurements are equal between genotype and DNA methylation.

We compared the microarray measurements between genotype and DNA methylation of the probes selected in common (Figure 7) and found that the microarray DNA methylation measurements were always less than the genotype measurements. Table 5 shows the results of the *t*-test applied to microarray measurements between genotype and DNA methylation. This observation is interesting, because a less methylated promoter usually indicates a more expressive genes, although not all the selected SNPs with DNA methylation were in the promoter region of the genes (identified as "upstream" in Additional file 1). To check that the demethylation was not because of inaccurate microarray measurement normalization, we randomly sampled the same number of SNPs as those in Tables 1, 2, and 3 1,000 times, and computed *P-*values adjusted by the BH criterion [26]. We found that typically less than 1 % of the trials had adjusted *P-*vales *<*0.05 (Table 6). Thus, we determined that there were no normalization biases in the data sets and the low observed *P-*values shown in Table 5 were not obtained because of fluctuations.

**Table 5:** *t*-tests of microarray measurements between genotype and DNA methylation for blood, normal and tumor tissues.

Nsp			
	Genotype	DNA methylation	*P-*value
	
blood	1.32	1.19	3.1 × 10^*−*12^
normal tissue	2.45	1.75	∗
tumor	2.84	2.23	∗

Sty1			

	Genotype	DNA methylation	*P-*value
	
blood	2.21	1.77	∗
normal tissue	2.58	2.14	∗
tumor	2.87	2.48	∗

Sty2			
	
	Genotype	DNA methylation	*P-*value

blood	1.69	1.29	∗
normal tissue	2.42	2.02	∗
tumor	2.51	2.04	∗

*Nsp and Sty are the microarray data sets used in the study*. Sty1 and Sty2 correspond to the PC4 for genotype (Figures 4d and 6b)/PC3 for DNA methylation (Figures 5c and 6c) and PC3 for genotype (Figures 4c and 6a)/PC4 for DNA methylation (Figures 5d and 6d) combinations of PCs. ∗ indicates P-values **<***2*.*2* x *10^−1^^6^*.

**Table 6:** *t*-tests of randomly sampled SNPs between genotype and DNA methylation.

Nsp			
	Lower bound	Upper bound	Number of significant *P-*values
	
blood	6.9 × 10^*−*4^	1.00	19
normal tissue	0.32	1.00	0
tumor	0.96	1.00	0

Sty1			

	Lower bound	Upper bound	Number of significant *P-*values
	
blood	0.05	1.00	5
normal tissue	0.01	1.00	4
tumor	0.04	1.00	6

Sty2			
	
	Lower bound	Upper bound	Number of significant *P-*values

blood	6.12 × 10^*−*4^	1.00	2
normal tissue	0.06	1.00	0
tumor	9.56 × 10^*−*3^	1.00	1

*P-values were computed using t-tests for the microarray measurements between genotype and DNA methylation for 1000 independent sets of randomly sampled SNPs. Each of the sets contained at least as many *SNPs as are included in Table 4. Nsp and Sty are the microarray data sets used in the study. Sty1 and Sty2 correspond to the PC4 for genotype (Figures 4d and 6b)/ PC3 for DNA methylation (Figures 5c and 6c) and PC3 for genotype (Figures 4c and 6a)/ PC4 for DNA methylation (Figures 5d and 6d) combinations of PCs. P-values, adjusted by the B*H criterion, of < 0.05 were regarded as significant*.

### Structure prediction of the proteins associated with selected genes

Although we selected genes with genotype-specific DNA methylation, for therapeutic purposes, we need to design drugs for the proteins that are encoded by these genes. To identify candidate drugs computationally, the tertiary structures of the target proteins are required as templates. However, the structures of many of the encoded proteins have not been reported.

To obtain the tertiary structure of these proteins, we used two protein structure prediction servers FAMS [18,19] and phyre2 [20,21] to predict the structure using only the amino acid sequence of the protein (see Additional file 5 for the amino acid sequences (in fasta format) that were used to predict the tertiary structures of the proteins).

The results of the protein structure predictions are summarized in Additional file 4. Some protein structures were already in the protein data bank (PDB) [27], if not, they were modeled using the structure of a suitable reference protein. These structures were then used as templates to predict drug candidates *in silico*.

For the proteins that were not in the PDB, for the reference proteins that were used for the structure prediction, we sought cancer-related papers that cited the reference proteins. The references to these papers are listed in Additional file 4. Most of reference proteins used for structure prediction were cancer-related. This finding also suggests that our gene selection process and protein structure prediction are plausible.

**Figure 8:**
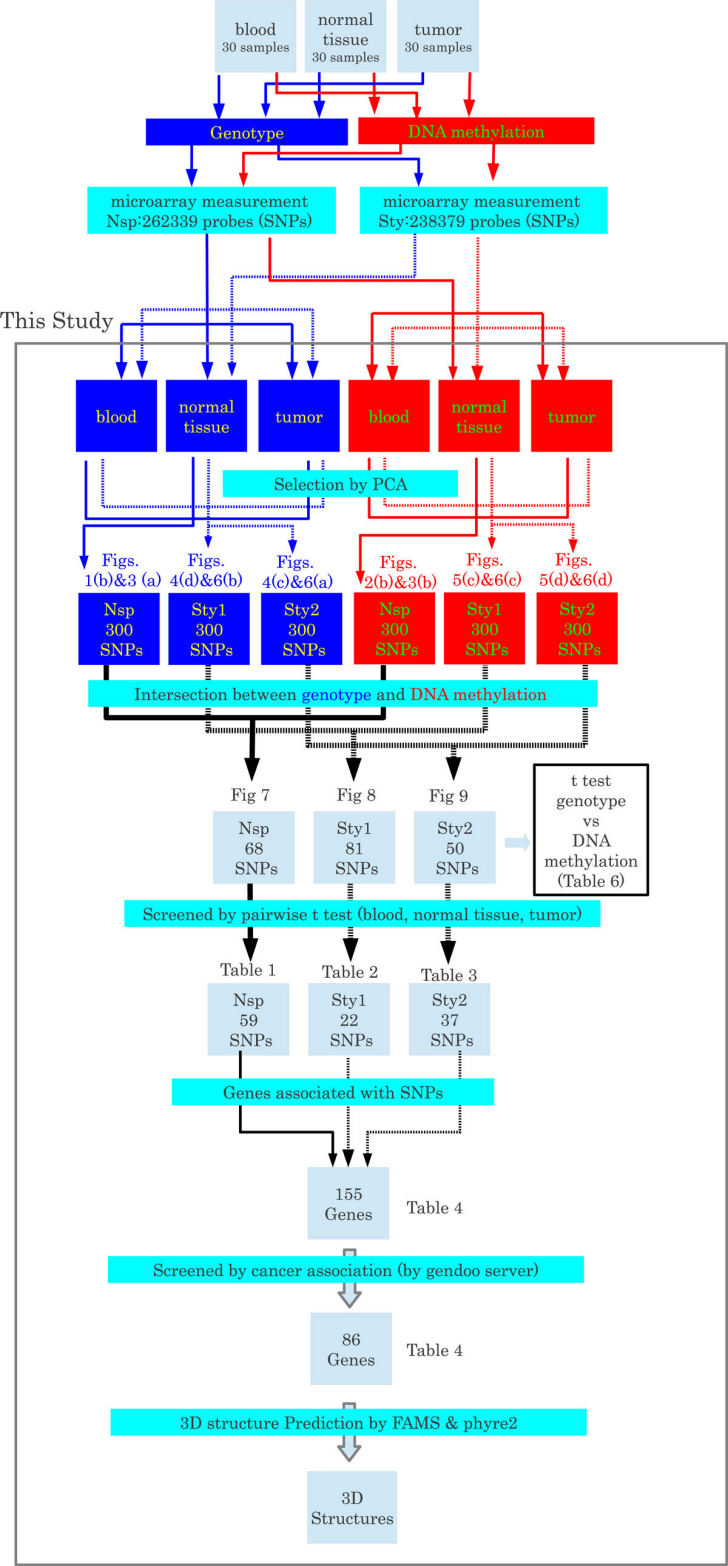
**Schematic illustration of the gene screening process**. The grey rectangle indicates the processes performed in this study. The red (blue) boxes indicate the data processing flow for the genotype (DNA methylation) data. The solid (dotted) lines indicate data processing flow for the Nsp (Sty) measurements. Sty1 and Sty2 indicate the two combinations of PCs that were used; PC4 for genotype (Figures 4d and 6b)/PC3 for DNA methylation (Figures 5c and 6c), and PC3 for genotype (Figures 4c and 6a)/PC4 for DNA methylation (Figures 5d and 6d).

A summary of the entire of gene selection processes is illustrated in Figure 8.

### *In silico *drug discovery

**Figure 9:**
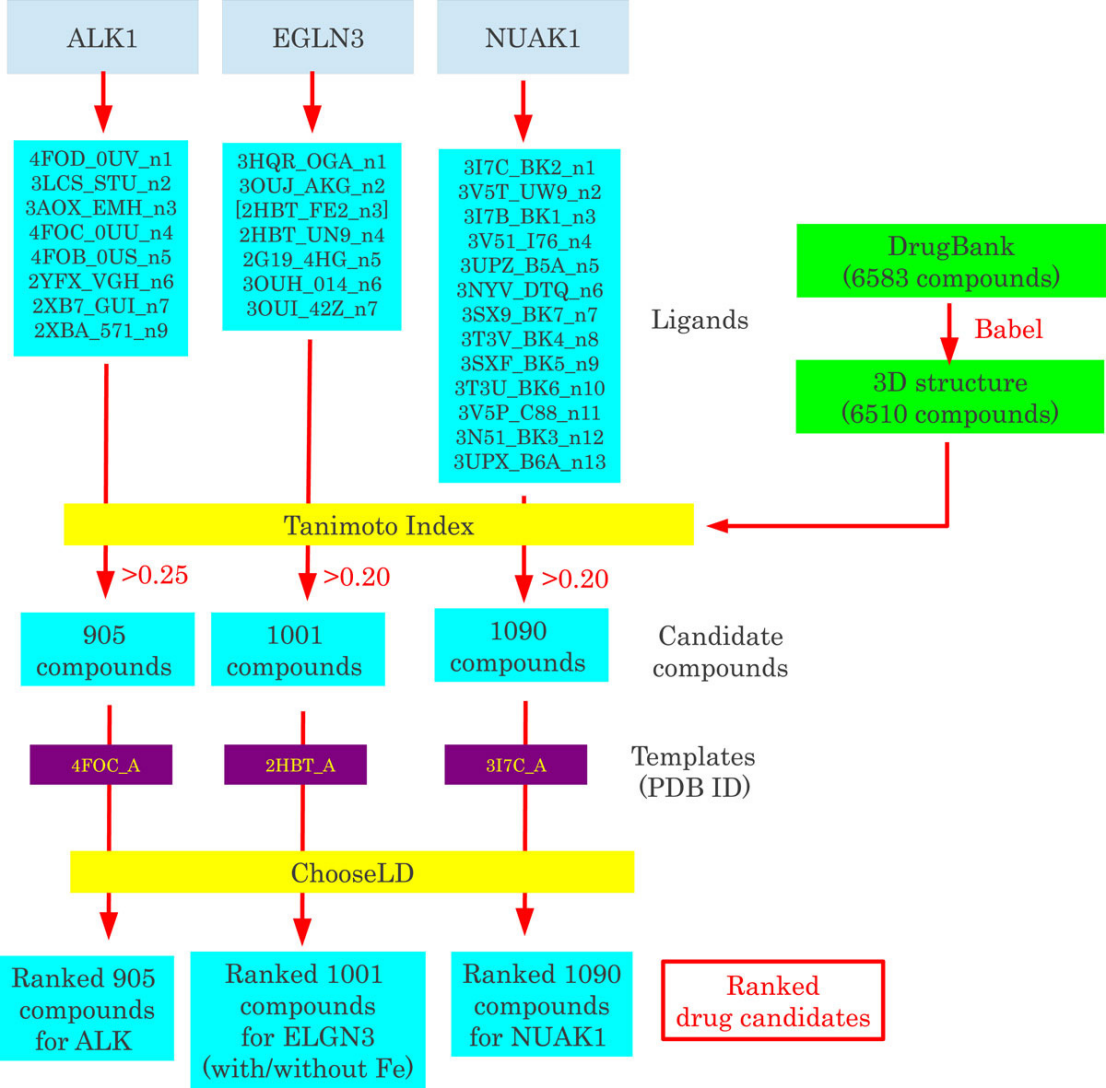
**Schematic illustration of the drug discovery process**. For the proteins encoded by the selected genes (ALK, EGLN3 and NUAK1), about 1,000 compounds, selected based on the Tanimoto index from DrugBank, were tested by ChooseLD using template protein structures from PDB. The templates are specified by their PDB IDs. The ligands are specified by the PDB ID, ligand name and a sequential number. For example, 3I7C_BK2_n1 indicates ligand BK2 (1-tert-butyl-3-naphthalen-2-yl-1H-pyrazolo[3,4-d]pyrimidin-4-amine) included in PDB entry 3I7C [PDB: 3I7C], and n1 means no.1. The drug discovery process for EGLN3 was performed twice, with and without Fe as a ligand. When Fe was excluded as a ligand, it was regarded as a mediator. That is, Fe bounds to the protein during docking simulation, but was excluded from the Tanimoto index computation.

We tried to design drugs that could bind to some of the protein templates using an *in silico *drug discovery method in which chemical compounds that potentially bind to proteins and suppress protein functions were sought computationally. For this purpose, we selected the three proteins encoded by ALK, EGLN3, and NUAK1 as drug targets, based upon a literature search and the gene annotations that indicated that these genes were expressed in cancer and had potentially functional binding pockets (e.g., protein kinase) for ligands. Details of the annotations are listed in Additional file 4. The drug discovery process that we used is illustrated in Figure 9 (see Methods for details).

After the FPAScores were estimated (see Methods and Figure 9), to check if three independent trials were feasible, we tested coincidence between three trials in two ways. First, we computed the correlation coefficients between three independent trials. For all pairwise computations for ALK, EGLN3, and NUAK1, the correlation coefficients were greater than 0.9. This suggests that the FRAScores computed by ChooseLD were highly reproducible. (For actual values of the correlation coefficients and scatter plots, see Additional file 6). However, the correlation coefficients represent the overall reproducibilities of FPAScores for the candidate drug compounds. It is more important that the compounds with higher FPAScores, i.e., those regarded as being highly reliable, were reproducible. Therefore, we checked how often the highly ranked compounds were selected between the three trials and found that the selection of the highly ranked compounds was also highly reproducible (see Additional file 7).

The ranking of the tested compounds based on their FPAScores are available as Additional file 8. The results are summarized in Table 7. Among the 10 top-ranked compounds for ALK, eight compounds targeted cancer genes, and two out of the eight targeted ALK. Among the 10 top-ranked compounds for ELGN3, including Fe as a ligand, eight compounds targeted cancer genes and two out of the eight targeted EGLN1, which is paralog of EGLN3. Among the 10 top-ranked compounds for ELGN3, without including Fe as a ligand but as a mediator, six were in common with the top-ranked compounds for EGLN3 when Fe was included as a ligand. Among the other four compounds, one targeted EGLN1. Of the 10 of the top-ranked compounds for NUAK1, most target more than 100 other genes and thus lack specificity. For a more detailed discussion about top 10 ranked compounds for ALK, EGLN3, and NUAK1, see Additional file 9. All of these findings suggested that the top-ranked compounds for each of the proteins were feasible candidate drugs.

**Table 7:** The 10 top-ranked compounds as drug targets for ALK, EGLN3, and NUAK1.

DrugBank ID	Compound name	Representative target cancer genes
	ALK	

DB01933	7-Hydroxystaurosporine	PDK1
DB08700	3-[(1R)-1-(2,6-dichloro-3-fluorophenyl)ethoxy]	**ALK**, c-MET, LCK,
	-5-(1-piperidin-4-yl-1H-pyrazol-4-yl)pyridin-2-amine	TRKA, TRKB, TIE2, ABL
DB04651	BIOTINOL-5-AMP	--
DB02491	4-[4-(1-Amino-1-Methylethyl)Phenyl]-5-Chloro-N	FGFR2
	-[4-(2-Morpholin-4-Ylethyl)Phenyl]Pyrimidin-2-Amine	
DB07006	9-HYDROXY-6-(3-HYDROXYPROPYL)-4	WEE1
	-(2-METHOXYPHENYL)PYRROLO[3,4-C]	
	CARBAZOLE-1,3(2H,6H)-DIONE	
DB02010	Staurosporine	ITK, SYK, MAPKAPK2, GSK3,
		CSK, CDK, PIK3CG, ZAP-70
DB02654	6-Hydroxy-Flavin-Adenine Dinucleotide	--
DB07460	2-({5-CHLORO-2-[(2-METHOXY-4-MORPHOLIN	**ALK**, PTK2
	-4-YLPHENYL)AMINO]PYRIMIDIN-4	
	-YL}AMINO)-N-METHYLBENZAMIDE	
DB07186	4-(4-METHYLPIPERAZIN-1-YL)-N-[5	AURKA, PLK1
	-(2-THIENYLACETYL)-1,5-DIHYDROPYRROLO	
	[3,4-C]PYRAZOL-3-YL]BENZAMIDE	
DB03247	Riboflavin Monophosphate	RPS6KA4, POR(P450), SGK1,
		NOS1, DPYD, DHODH

	EGLN3 (with Fe)	

DB03702	2-[4-[[(S)-1-[[(S)-2-[[(Rs)-3,3,3-Trifluoro-1-Isopropyl-2	CELA1
	-Oxopropyl]Aminocarbonyl]Pyrrolidin-1-Yl-]Carbonyl]-2	
	-Methylpropyl]Aminocarbonyl]Benzoylamino]Acetic Acid	
DB04761	PYRIMIDINE-4,6-DICARBOXYLIC ACID	MMP13
	BIS-[(PYRIDIN-3-YLMETHYL)-AMIDE]	
DB08687	N-[(1-CHLORO-4-HYDROXYISOQUINOLIN-3-YL)	**EGLN1**, PHD2
	CARBONYL]GLYCINE	
DB08131	2-{4-[2-(2-AMINO-4-OXO-4,7-DIHYDRO-3H	thyA
	-PYRROLO[2,3-D]PYRIMIDIN-5-YL)-ETHYL]	
	-BENZOYLAMINO}-3-METHYL-BUTYRIC ACID	
DB02718	5-Formyl-6-Hydrofolic Acid	--
DB02015	Dihydrofolic Acid	--
DB02031	(6s)-5,6,7,8-Tetrahydrofolate	NOS1, thyA
DB04760	PYRIMIDINE-4,6-DICARBOXYLIC ACID	MMP13
	BIS-(4-FLUORO-3-METHYL-BENZYLAMIDE)	
DB04759	PYRIMIDINE-4,6-DICARBOXYLIC ACID	MMP13
	BIS-(3-METHYL-BENZYLAMIDE)	
DB07112	N-[(4-HYDROXY-8-IODOISOQUINOLIN-3-YL)	**EGLN1**, PHD2
	CARBONYL]GLYCINE	
		

	EGLN3 (without Fe)	

DB08687	N-[(1-CHLORO-4-HYDROXYISOQUINOLIN	**EGLN1**, PHD2, HIF1A
	-3-YL)CARBONYL]GLYCINE	
DB03702	already listed in EGLN3 (with Fe)	
DB04759	already listed in EGLN3 (with Fe)	
DB03625	5,10-Dideazatetrahydrofolic Acid	GARFTase
DB04760	already listed in EGLN3 (with Fe)	
DB07112	already listed in EGLN3 (with Fe)	
DB02015	already listed in EGLN3 (with Fe)	
DB03541	10-Propargyl-5,8-Dideazafolic Acid	TYMS, DHFR
DB00158	Folic Acid	--
DB04761	already listed in EGLN3 (with Fe)	

	NUAK1	

DB08053	1-cyclobutyl-3-(3,4-dimethoxyphenyl)-1H	CSF1R and others
	-pyrazolo[3,4-d]pyrimidin-4-amine	
DB08052	1-cyclopentyl-3-(1H-pyrrolo[2,3-b]pyridin-5-yl)	CSF1R and others
	-1H-pyrazolo[3,4-d]pyrimidin-4-amine	
DB08054	1-(1-methylethyl)-3-quinolin-6-yl-1H	CSF1R and others
	-pyrazolo[3,4-d]pyrimidin-4-amine	
DB07563	1-{7-cyclohexyl-6-[4-(4-methylpiperazin-1-yl)	CTSK
	benzyl]-7H-pyrrolo[2,3-d]pyrimidin-2-yl}methanamine	
DB08035	1-TERT-BUTYL-3-(2,5-DIMETHYLBENZYL)	AR
	-1H-PYRAZOLO[3,4-D]PYRIMIDIN-4-AMINE	
DB04463	3-(4-Amino-1-Tert-Butyl-1h-Pyrazolo[3,4-D]	CBR1
	Pyrimidin-3-Yl)Phenol	
DB08300	1-methyl-3-naphthalen-2-yl-1H-pyrazolo	CSF1R and others
	[3,4-d]pyrimidin-4-amine	
DB01809	1-Ter-Butyl-3-P-Tolyl-1h-Pyrazolo	PKD1 and others
	[3,4-D]Pyrimidin-4-Ylamine	
DB08461	3-[(4-AMINO-1-TERT-BUTYL-1H-PYRAZOLO[3,4-D]	AR
	PYRIMIDIN-3-YL)METHYL]PHENOL	
DB08699	1-tert-butyl-3-(3-methylbenzyl)-1H-pyrazolo	CAMK2G
	[3,4-d]pyrimidin-4-amine	

The compounds were ranked based on FPAScores averaged over three independent trials and their representative target cancer genes. For the full lists of ranked compounds and a detailed discussion of the target cancer genes listed here, see Additional files 7 and 8, respectively. ALK and EGLN1, a paralog of EGLN3, are in bold letters. "―" indicates that no known cancer-associated genes are targeted by these compounds.

## Conclusion

In this paper, we investigated genotype-specific DNA methylation in esophageal squamous cell carcinoma, using principal component analysis. We identified more than 100 genotype-specific DNA methylation SNPs associated with the disease. Among 155 genotype-specific DNA methylation associated genes, 86 were associated with cancers using the Gendoo server. The structures of proteins encoded by selected genotype-specific DNA methylation associated genes were predicted successfully using two profile based methods, FAMS and Phyre2. Candidate drug compounds were screened using the Tanimoto index from DrugBank and were evaluated by ChooseLD for three selected proteins, ALK, EGLN3 and NUAK1. The selected drug candidates were promising starting points for future studies.

## Authors' contributions

MI, HU, and YHT planned the research study. RK and YHT performed the gene screening. MI and HU performed the protein structure prediction using FAMS and *in silico *drug discovery using ChooseLD. MI, HU, and YHT wrote the paper.

## Supplementary Material

Additional file 1 **Annotation of selected SNPs**. Annotation information of selected SNPs (from Tables 1, 2 and 3) including a list of genes associated with each SNP. Click here for file

Additional file 2 **Genes with significant genotype-specific DNA methylation**. Nsp and Sty are the microarray data sets used in the study. Sty1 and Sty2 correspond to the PC4 for genotype (Figures 4a and 6b)/PC3 for DNA methylation (Figures 5c and 6c) and the PC3 for genotype (Figures 4c and 6a)/PC4 for DNA methylation (Figures 5d and 6d) combinations of PCs, respectively. The genes indicated in bold letters were associated with at least one cancer-related disease due to Gendoo [13,14]. Click here for file

Additional file 3 **Association of cancer related diseases with genes based on the Gendoo server**. List of cancer-related diseases associated with the genes indicated in bold letters in Additional file 2 based on the Gendoo server [13,14]. Associations with "Esophageal Neoplasms" and "Carcinoma, Squamous Cell" are highlighted. Click here for file

Additional file 4 **List of references that report the association of the selected genes with cancer-related diseases**. The selected genes are those listed in Additional file 2. The list also includes the performance of the protein structure prediction and the references that associate the proteins that were used as reference proteins to predict protein structure with cancer-related diseases. Click here for file

Additional file 5 **Amino acid sequences for the proteins encoded by the selected genes**. The amino acid sequences were used for protein structure predictions and are listed in fasta format. Click here for file

Additional file 6 **Pearson correlation coefficients and scatter plots between independent trials for FPAScore computation**. (a) ALK; (b) EGLN3 with Fe. (c) EGLN3 without Fe. (d) NUAK1. Scatter plots are shown for reference. Red diagonal lines indicate that FPAScores were identical between two trials. Click here for file

Additional file 7 **Number of compounds selected in common in three trials as highly feasible drug candidate compounds**. The number of common selections from among the top k ranked compounds in three trials (red circles). Black solid line indicates the expected number of compounds to be selected in common between three trials when the three trials are not correlated at all (n0(k), see Methods for details). Blue straight line indicates a complete match between the three trials. (a) ALK. (b) EGLN3 with Fe. (c) EGLN3 without Fe. (d) NUAK1. It is clear that the number of selections in common is much less than would be expected for random selections, n0(k), and is very close to a complete match (blue line). Click here for file

Additional file 8 **Full list of ranked compounds**. List of compounds ranked based on the FPAScores averaged over three independent trials, for ALK, EGLN3 (with and without Fe), and NUAK1. Click here for file

Additional file 9 **Detailed discussion of the top 10 compounds listed in Table **7. Target protein information and protein inhibition information were taken from DrugBank [22,23] and ChEMBL [28,29]. Click here for file

## Declaration

This research was funded by KAKENHI, 23300357 and Chuo University Joint Research Grant.

This article has been published as part of *BMC Systems Biology *Volume 8 Supplement 1, 2014: Selected articles from the Twelfth Asia Pacific Bioinformatics Conference (APBC 2014): Systems Biology. The full contents of the supplement are available online at http://www.biomedcentral.com/bmcsystbiol/supplements/8/S1.
