# Supplementary material for: Genes associated with genotype-specific DNA methylation in squamous cell carcinoma as candidate drug targets
Source: BMC Syst Biol. 2014 Jan 24;8(Suppl 1):S4. doi: 10.1186/1752-0509-8-S1-S4 (PMC4080267; doi:10.1186/1752-0509-8-S1-S4)
Supplement: Additional file 2 — Genes with significant genotype-specific DNA methylation. Nsp and Sty are the microarray data sets used in the study. Sty1 and Sty2 correspond to the PC4 for genotype (Figures 4a and 6b)/PC3 for DNA methylation (Figures 5c and 6c) and the PC3 for genotype (Figures 4c and 6a)/PC4 for DNA methylation (Figures 5d and 6d) combinations of PCs, respectively. The genes indicated in bold letters were associated with at least one cancer-related disease due to Gendoo [13,14]. [file 1752-0509-8-S1-S4-S2.pdf]

	Gene symbol	Nsp	Sty 1	Sty 2
1	A2BP1	○		
2	ADK			○
3	AFF3			○
4	**ALK**			○
5	ALS2CR13	○		
6	**ASB3**			○
7	**AVO3**	○		
8	**AVPR1A**	○		
9	**B3GNT5**	○		
10	**BCL6**	○		○
11	BICD1	○	○	
12	BMP2	○		
13	**BRF1**	○		
14	C10orf26			○
15	C18orf18	○	○	
16	**C1GALT1**		○	
17	C20orf114			○
18	C20orf129	○		○
19	C22orf13			○
20	C3orf21			○
21	**CA10**	○		
22	**CAMK1D**	○		
23	**CBLB**	○		
24	**CCND1**		○	
25	**CCNL1**			○
26	**CD36**	○		
27	**CD96**	○		
28	**CKAP4**			○
29	**CLYBL**			○
30	COL22A1			○
31	CPAMD8	○		
32	**CRABP1**			○
33	DHX35	○		○
34	DKFZp564N2472	○		
35	DLGAP1	○	○	
36	DNAH11	○		
37	**DNCI1**			○
38	**EDN2**	○		
39	**EGLN3**		○	
40	**EVI1**		○	
41	**EXT1**	○		
42	**F2RL3**	○		
43	**FADD**		○	
44	FAM84B			○
45	**FGF12**		○	
46	**FGF19**	○		
47	**FGF3**		○	○
48	**FH**			○
49	FLJ10652	○	○	
50	FLJ16641			○
51	FLJ20323		○	
52	FLJ32110	○		
53	FLJ32955	○		
54	FLJ33360		○	
55	FLJ42393	○	○	○
56	FLNB			○
57	FLRT2	○		
58	FSIP2	○		
59	GLIS3			○
60	GMEB2	○		
61	GOLPH4		○	
62	**GRHL2**		○	
63	**GZMB**			○
64	HAO1	○		
65	**HEBP1**	○		
66	**HEY1**		○	
67	**ICA1**	○		
68	**IL1RAP**			○
69	**IMP-2**		○	
70	IMPA1			○
71	**IREB2**			○
72	**K6HF**	○		
73	KCNB2		○	
74	KCNMB3		○	
75	**KCNN3**			○
76	KIAA1467	○		
77	KIF2B	○		
78	**KLHL6**	○		
79	KRTHB2	○		
80	LOC131368	○		
81	LOC387882	○		
82	LOC494143			○
83	LOC92196		○	
84	LONRF2			○
85	**LPP**		○	
86	LYPLAL1	○		
87	**MAFB**	○		○
88	**MCF2L2**	○		
89	MGC2654	○		
90	**MGC26647**	○		
91	MGC33530	○		
92	**MYEOV**		○	
93	MYO1B	○		
94	**MYST4**			○
95	NFKBIZ	○		
96	**NOP5/NOP58**	○		
97	NPAS3		○	
98	**NRXN1**			○
99	**NUAK1**	○		○
100	**NXPH1**	○		

	Gene symbol	Nsp	Sty 1	Sty 2
101	OSTN			○
102	**PACS1**			○
103	PDE4DIP	○		
104	**PDK4**			○
105	**PIGO**			○
106	**PIWIL1**			○
107	PKIA	○		
108	**PKP4**		○	
109	**PLA2G4A**	○		
110	**PLUNC**			○
111	**PPFIA1**		○	
112	PSARL		○	
113	**PTPRT**			○
114	**PVRL3**	○		
115	PXMP3	○		
116	**RAI17**	○		
117	**RAP2B**	○		
118	**RAPGEF5**	○		
119	RASL12			○
120	RGS7			○
121	**RND3**	○		
122	**RPL14**	○		
123	**SAMD12**	○		
124	SCHIP1	○		
125	SCMH1	○		
126	SDK1	○		
127	**SEMA3C**	○		
128	**SEMA3E**	○		
129	**SENP2**	○		
130	**SFRS10**		○	
131	SFXN2			○
132	SLC7A14	○		
133	**SMAD3**			○
134	**STAT4**	○		
135	STAU2	○		
136	**STMN2**		○	
137	**STXBP6**			○
138	TARBP1	○		
139	**TGFB2**	○		
140	TMC1			○
141	**TMEM16A**		○	○
142	TNIK			○
143	**TOMM7**	○		
144	TPCN2		○	
145	UNQ739			○
146	**UPB1**			○
147	VPS41	○		
148	**ZA20D2**			○
149	**ZBED2**	○		
150	**ZBTB20**			○
151	**ZFPM2**	○		
152	**ZNF138**	○		
153	**ZNF588**	○		
154	**ZNF639**		○	
155	**raptor**	○		
