# Supplementary material for: Genes associated with genotype-specific DNA methylation in squamous cell carcinoma as candidate drug targets
Source: BMC Syst Biol. 2014 Jan 24;8(Suppl 1):S4. doi: 10.1186/1752-0509-8-S1-S4 (PMC4080267; doi:10.1186/1752-0509-8-S1-S4)
Supplement: Additional file 4 — List of references that report the association of the selected genes with cancer-related diseases. The selected genes are those listed in Additional file 2. The list also includes the performance of the protein structure prediction and the references that associate the proteins that were used as reference proteins to predict protein structure with cancer-related diseases. [file 1752-0509-8-S1-S4-S4.pdf]

# Supporting Information: Previous studies that reported the relationship between cancers/tumors and genes bold faced in Table 4 or reference proteins used for protein structure prediction

Ryoichi Kinoshita[1] and Mitsuo Iwadate[2] and Hideaki Umeyama[2] and Y-h. Taguchi*[1]

[1]Department of Physics, Chuo University, 1-13-27 Kasuga, Bunkyo-ku, Tokyo 112-8551, Japan
[2]Department of Biological Science, Chuo University, 1-13-27 Kasuga, Bunkyo-ku, Tokyo 112-8551, Japan

Email: Mitsuo Iwadate - iwadate@bio.chuo-u.ac.jp; Hideaki Umeyama - umeyama@bio.chuo-u.ac.jp; Y-h. Taguchi - tag@granular.com;

*Corresponding author

## Citations related to cancer and reference proteins

In the following, we cited references related to each gene one by one. After **Reference Protein**, we denote information about reference proteins.

First comes the name(s) of reference protein(s). Then modelling information comes as follows.

FAMS: $P$-values computed by blast, aligned region of target protein (total length of target protein), aligned region of reference protein (total length of reference protein), sequence identity.

phyre2: Confidence (max 100), aligned region of target protein (total length of target protein), aligned region of reference protein (total length of reference protein), sequence identity.

### ALK

The anaplastic lymphoma kinase (ALK) has been found to be altered in several solid and hematologic tumors [1].

**Reference Proteins**

ALK was modeled using ABL1 by;
FAMS

[$p = 1 \times 10^{-122}$,969-1394(1620),66-458(465),25%]

and
phyre2

[Conf.100,1107-1394(1620),187-458(465),40%].

Since ABL1 is abbreviation of "Abelson murine leukemia viral oncogene homolog 1", it is oncogenic as its name says.

### ASB3

Ankyrin repeat and SOCS box 3 (ASB3) mediates ubiquitination and degradation of tumor necrosis factor receptor II [2].

**Reference Proteins**

ASB3 was modeled using Ankyrin by;
FAMS

[ $p = 1 \times 10^{-104}$, 12-398(518), 2-382(404), 24%]

and
phyre2

[Conf. 100,12-380(518), 2-353(404), 21%].

### AVPR1A

AVPR1A is an interesting gene and was investigated from the various points of views. For example, musical aptitude was reported to be associated with AVPR1A-haplotypes [3]. Alternatively, AVPR1A was examined as an autism susceptibility gene [4]. There are no studies that report relationship with cancers.

**Reference proteins**

AVPR1A was modeled using $\beta$2-adrenergic receptor (ADRB2) by;
FAMS

[ $p = 1\times10^{-102}$, 25-364(418), 137-443(443), 17%]

and
phyre2

[Conf. 100, 47-365(418), 1-441(442), 20%].

There were several studies that report the relationship between ADRB2 and cancers [5]. Preclinical studies have shown that ADRB signalling can inhibit multiple cellular processes involved in breast cancer progression and metastasis, including extracellular matrix invasion, expression of inflammatory and chemotactic cytokines, angiogenesis and tumour immune responses [6]. The ADRB2 and Her2 comprise a positive feedback loop in human breast cancer cells [7].

## B3GNT5

B3GNT5 is known to be Lactotriaosylceramide synthase that is related to glycosphingolipid (GPL) synthesis. GPLs were known to be important for embryonic differentiation and development [8,9]. B3GNT5 was involved in seventeen genes that were shown to be differentially expressed between the tumours and the paired normal [10].

**Reference proteins**

B3GNT5 that was modeled using MFNG by;
FAMS

[ $p = 3 \times 10^{-96}$, 89-373(378), 6-240(243), 14%]

and
phyre2

[Conf. 99.9, s 90-365(378), 7-242(243), 23%]

MFNG has also been shown to inhibit Jagged-Notch signalling in cervical cancer cells. Introduction of MFNG into the CaSki cervical cancer cell line leads to inhibition of its tumorigenicity [11].

## BCL6

BCL6 is a zinc-finger transcriptional repressor known for its oncogenic role in B cell lymphoma and was expected to be a novel target for therapy of Ph+ B cell acute lymphoblastic leukemia [12]. Inhibitor of BCL6 was reported to kill diffuse large B-cell lymphoma(DLBCL) cells in vitro and in vivo [13]. Other than leukemia, BCL6 was also reported to be expressed in breast cancer and to prevent mammary epithelial differentiation [14].

**Reference proteins**

BCL6 is in PDB. But, structure was known only partially [5-129 (706)].

## BRF1

BRF1, also known as TFIIIB, that was required for transcription initiation at RNA polymerase III promoters consists of subunits, TBP, Bdp1 and Brf1. RNA polymerase (pol) III transcription is specifically elevated in a variety of cancers and is a target of regulation by a variety of tumor suppressors and oncogenes. Deregulation of RNA polymerase III transcription was hypothesized to be mediated by altered TFIIIB expression [15]. In actual, the TFIIIB subunits Brf1 and Brf2 were reported to be differentially expressed in a variety of cancer cell lines [15]. Thus, it is not strange if BRF1 plays critical roles in cancer formation.

**Reference proteins**

BRF1 was modeled using TFIIB by;
FAMS

[ $p = 3 \times 10^{-77}$, 82-281(677), 1-197(207), 21%]

and
phyre2

[ Conf, 100, 83-267(677), 2-158(207), 24%]

Overexpression of TFIIB-related factor 2 is significantly correlated with tumor angiogenesis and poor survival in patients with esophageal squamous cell cancer [16].

## C1GALT1

Core 1 synthase, glycoprotein-N-acetylgalactosamine 3-beta-galactosyltransferase, 1 (C1GALT1) is known to synthesize O-Glycan (Mucin type O-Glycan biosynthesis). Mucin type O-Glycan biosynthesis is also known to be related to tumor formation as well as development [17,18].

**Reference proteins**

C1GALT1 was modeled using MFNG O-Fucosylpeptide 3-Beta-N-Acetylglucosaminyltransferase by;
FAMS

[ $p = 1 \times 10^{-113}$, 88-333(363), 6-243(243) 15%]

phyre2

[ Conf. 100, 87-329(363), 5-242(243), 18%]

MFNG was also reported to be structurally homologous to B3GNT5 (see above).

## CA10

Carbonic anhydrase 10 (CA10) was reported to be one of six genes that were employed as methylation markers for bladder cancer [19].

**Reference proteins**

CA10 was modeled using other carbonic anhydrase, CA7 by FAMS

[ $p = 1 \times 10^{-107}$, 30-303(328), 2-262(262), 29%]

CA1 by phyre2

[ Conf. 100, 31-301 (328), 2-258(258), 30%],

Among carbonic anhydrase, CA9 that is the only tumor-associated carbonic anhydrase isoenzyme known [20]. Other than CA9, some carbonic anhydrase were reported to be related to cancer. For example, CA1 was reported to be as a new plasma biomarker for prostate cancer [21]. Carbonic anhydrase inhibitors were recognized as promising antiobesity drugs, too [22].

## CAMK1D

Calcium/calmodulin-dependent protein kinase ID (CAMK1D) was found to have subtypes related to severe outcome of breast cancer [23]. CAMK1D SNP was also reported to be associated with BRCA1 and BRCA2 mutation related to breast cancer risk [24].

**Reference proteins**

CAMK1D is in PDB.

## CBLB

CBLB was reported to be one of five genes whose mutation constitute molecular events in chronic myelogenous leukemia [25]. Spontaneous tumor rejection by CBLB-deficient CD8+ T cells was also observed [26]. Thus, CBLB is clearly an important factor for tumor formation.

**Reference protein**

Although CBLB is in PDB, CBLB was modeled using CBL2 by

FAMS

[ $p = 0$, 35-426(982), 1-388(388), 85%];

and

phyre2

[Conf. 100, 39-426(918), 3-388(388), 85%]

CBL2 was suggested to be one of possible candidate genes for familial leukemia with anticipation [27].

## CCND1

Cyclin D1 (CCND1) downregulation was known to contributes to anti-cancer effect of isorhapontigenin (ISO) on human bladder cancer cells [28]. CCND1 protein overexpression and CCND1 amplification was observed in breast carcinomas [29]. Amplification of CCND1 was involved in the Wnt and fibroblast growth factor signaling pathways, suggests that these pathways may be activated in esophageal carcinoma cell lines [30]. CCND1 in laryngeal squamous cell carcinoma was reported to have higher expression levels in comparison to healthy controls [31]. MiR-138 suppressed nasopharyngeal carcinoma growth and tumorigenesis by targeting the CCND1 oncogene [32].

**Reference proteins**

Although CCND1 is in PDB, CCND1 is modeled using ;

CCND3 by FAMS

[ $p = 6 \times 10^{-65}$, 24-258(295), 2-229(229), 55%]

and

CDK6 by phyre2

[ Conf. 100, 22-257(295), 1-228(233), 20%]

CCND3 was validated as the direct target of miR-138 that was one of 11 down-regulated miRNAs in hepatitis B virus-associated hepatocellular carcinoma [33]. CDK6 was also the direct target of miR-138 that was down-regulated in glioblastoma multiforme [34].

## CCNL1

CCNL1 was known to be altered in human head and neck squamous cell carcinoma [35].

Reference protein:

CCNL1 was modeled using cyclin k by;

FAMS

[ $p = 9 \times 10^{-91}$, 53-300 (526),7-254 (254), 28%]

and

phyre2

[Conf 100, 48-277(526),2-233(254), 31%].

cyclin k was known to be oncogenic [36].

## CD36

CD36 repression was reported to activate a multicellular stromal program shared by high mammographic density and tumor tissues [37]. CD36-mediated activation of endothelial cell apoptosis by an N-terminal recombinant fragment of

thrombospondin-2 inhibits breast cancer growth and metastasis in vivo [38].

**Reference proteins**

Neither FAMS nor phyre2 could model CD36 with any reliable reference proteins.

## CD96

CD96 was known to be a leukemic stem cell-specific marker in human acute myeloid leukemia [39]. Wilms tumor 1 gene mutation-associated gene-expression signature included CD96 [40].

**Reference proteins**

CD96 was modeled using ;

TTN by FAMS

[ $p = 1 \times 10^{-69}$, 23-571(585), 4-493(569),11%]

PIGR by phyre2

[Conf. 99.9,26-486(585),1-441(585), 13%]

Mutations in the gene encoding the giant muscle proteins titin (TTN) have been associated with cardiac and skeletal myopathies in humans [41]. PIGR expression in cancer cells was shown in vitro [42]

## CKAP4

Antiproliferative factor decreases Akt phosphorylation and alters gene expression via CKAP4 in T24 bladder carcinoma cells [43].

**Reference proteins**

CKAP4 was modeled using colicin ia by;

FAMS

[ $p = 3 \times 10^{-62}$, 48-428(602), 64-457(602), 9%]

and

phyre2

[Conf. 98.9, 77-516(602), 9-458(602), 10%]

Tumor targeting antibody was generated by mimicing toxin colicin ia [44].

## CLYBL

CLYBL was reported to be expressed differently between bladder UCs and UCs and renal pelvis [45].

**Reference proteins**

CLYBL was modeled using malate synthase by;

FAMS

[ $p = 1 \times 10^{-92}$, 3-338 (340), 34-434(501), 13%]

and

phyre2

[Conf. 100, 36-339 (340), 1-324(349), 18%].

Malate synthase catalyse carboxymethyl-forming synthesis of acetyl-CoA while acetyl-CoA carboxylase is known to be a novel target for cancer therapy [46].

## CRABP1

CRABP1 was suggested to be included into tumorigenesis of some adenomas subtypes [47]. CRABP1-reduced expression is associated with poorer prognosis in serous and clear cell ovarian adenocarcinoma [48].

**Reference proteins**

CRABP1 is in PDB.

## DNCI1

DNCI1, also termed as DYNC1I1, was reported to be located in Hepatocellular carcinoma amplified region, 7q21.3 [49].

**Reference proteins**

DNCI1 was modeled using WD-40 repeat by;

FAMS

[ $p = 1 \times 10^{-145}$, 75-642(645), 6-569(577), 12%]

and phyre2

[Conf. 100, 200-606(645), 9-577(577), 29%].

Ciao 1, WD40 protein, was reported to interact with the tumor suppressor protein WT1 [50].

## EDN2

Forced expression of ET-2 and ET-3 was conducted in human colon cancer cells followed by real-time cell migration and invasion assays [51]. EDN2 were found to be commonly overexpressed in a broad range of human tumour entities [52–54].

**Reference proteins**

FAMS did not predict reliable structure for EDN2. The structure predicted by phyre2 is too short compared with total length [49-72(178)] and thus useless.

## EGLN3

Glioma progression was reported to be suppressed by EGLN3 [55]. EGLN3 was reported to be inactivated by methylation in plasma cell neoplasia [56]. VHL-mutated tumors showed an unexpected overexpression of EGLN3 mRNA [57].

**Reference proteins**

EGLN3 was modeled using EGLN1 by;

FAMS

[$p = 4 \times 10^{-86}$, 5-227(239),1-222(225),63%]

and

phyre2

[ Conf. 100, 9-225(239), 1-216(216), 66%]

EGLN1 was one of twelve newly identified proteins differentially expressed in cancer-associated fibroblasts [58].

## EVI1

Overexpression of EVI1 was reported to repress TGF-$\beta$ signaling in colorectal cancer [59]. Frequent EVI1 translocations in myeloid blast crisis CML that evolves through tyrosine kinase inhibitors [60].

**Reference proteins**

EVI1 was modeled using

GTF3A by FAMS

[ $p = 7 \times 10^{-31}$, 787-978(1051), 4-182(182), 10%]

and

PRDM9 by phyre2

[Conf. 100, 16-212(1051), 4-214(215), 14%]

But the predicted structure is too short compared with total length thus useless.

## EXT1

Fibroblast EXT1-levels influence tumor cell proliferation and migration in composite spheroids [61]. Mutation analysis and prenatal diagnosis of EXT1 gene mutations in Chinese patients with multiple osteochondromas [62].

**Reference proteins**

EXT1 was modeled using EXTL2 by;

FAMS

[ $p = 1 \times 10^{-128}$, 478-736(746), 3-265(265), 15%]

and;

phyre2

[ Conf. 100, 476-705(746), 1-221(251), 30%]

EXTL2 was a member of tumor suppressor EXT familty together with EXT1 [63].

## F2RL3

F2RL3 (PAR4) was often reported to be related to cancer [64–67].

**Reference proteins**

F2RL3 was modeled using CHRM2 by;

FAMS

[$p = 4 \times 10^{-69}$, 74-355(385), 1-277(278), 19%]

and

phyre2

[Conf. 100, 78-355(385), 5-437(438), 27%]

CCRM2 was hypermethylated in gastric cancer [68].

## FADD

FADD protein release mirrors the development and aggressiveness of human non-small cell lung cancer [69]. High levels of phosphorylated FADD (p-FADD) in tumor cells correlate with increased activation of the antiapoptotic transcription factor NF-*kappa*B and is a biomarker for aggressive disease and poor clinical outcome [70].

**Reference proteins**

FADD was in PDB.

## FGF12

Increased FGF12 expression was observed in diffuse type of gastric cancer [71]. Tumour-endothelium interactions in co-culture was reported to elevate FGF12 expression [72]. FGF12 was one of 70 significantly hypermethylated genes in breast cancer tissues [73].

**Reference protein**

FGF12 was modeled using FGF13 by;

FAMS

[$p = 7 \times 10^{-61}$, 68-216(243), 1-149(149), 76%]

and

phyre2

[ Conf. 100, 68-216(243), 1-149(1-149), 77%]

There are no reports that suggest the relationship between FGF13 and cancer/tumor.

## FGF19

Endocrine fibroblast growth factor FGF19 promotes prostate cancer progression [74]. Latasa et al suggested that combined targeting of FGF19 and AR/EGFR may enhance therapeutic efficacy [75]. Amplification of FGF19 was involved in the Wnt and fibroblast growth factor signaling pathways, suggests that these pathways may be activated in esophageal carcinoma cell lines [30].

**Reference proteins**

FGF19 was in PDB. FGF19 was also modeled using other FGF proteins that are widely known to be related to cancer [76].

## FGF3

FGF3/FGF4 amplification and multiple lung metastases in responders to sorafenib in hepatocellular carcinoma [77] . Amplification of FGF3 was involved in the Wnt and fibroblast growth factor signaling pathways, suggests that these pathways may be activated in esophageal carcinoma cell lines [30].

**Reference proteins**

FGF3 was also modeled using various FGF proteins.

## FH

Hereditary leiomyomatosis and renal cell carcinoma was characterized by germline mutation of Fumarate Hydratase (FH) [78]. Downregulation of FH is related to tumorigenesis in sporadic renal cell cancer [79].

**Reference proteins**

FH was in PDB.

## GRHL2

Relative expression of a GRHL2-mediated gene-set pair was reported to predict breast cancer metastasis [80]. GRHL2 was also reported to determine the epithelial phenotype of breast cancers and promotes tumor progression [81]. GRHL2 was known to be concordantly deregulated by genomic aberration in gastric cancer [82].

**Reference Proteins**

FAMS did not predict reliable structure for GRHL2. The structure predicted by phyre2 is too short compared with total length [483-555(625)] and thus useless.

## GZMB

EGFR-targeted granzyme B (GZMB) expressed in NK cells enhances natural cytotoxicity and mediates specific killing of tumor cells [83].

**Reference Proteins**

GZMB is in PDB.

## HEBP1

Knockdown of HEBP1, also known as NFE2L2 or NRF2, inhibits the proliferation and growth of U251MG human glioma cells in a mouse xenograft model [84]. HEBP1 prevents initiation but accelerates progression through the Kras signaling pathway during lung carcinogenesis [85].

**Reference proteins**

FEBP1 was in PDB.

## HEY1

HEY1 was reported to be inhibited treatment of mice with EA that inhibits human pancreatic cancer growth in Balb c nude mice [86]. HEY1 was reported to be one of genes down-regulated in SCUBE3-knockdown tumors, while SCUBE3 is strongly expressed in extremely invasive lung carcinoma [87].

**Reference proteins**

FAMS did not predict reliable structure for HEY1. The structure predicted by phyre2 is too short compared with total length [50-110(304)] and thus useless.

## ICA1

ICA1, also known as ICA69, was reported to be a novel Rab2 effector regulating ER-Golgi trafficking in insulinoma cells [88]. ICA1 was mutated in 3 of 9 high-level microsatellite instability (MSI-H) inflammatory bowel disease-related neoplasms vs 2 of 54 sporadic MSI-H colorectal cancers (P = .028) [89].

**Reference proteins**

ICA1 was modeled using BIN2;
FAMS
[ $p = 1 \times 10^{-65}$, 24-259(483), 1-224(230), 13%]
phyre2
[Conf. 99.8, 26-257(483), 3-222(230), 13%]
Bridging integrator-2 (Bin2) is also called Breast cancer-associated protein 1 (BRAP1).

## IL1RAP

IL1RAP was reported to be overexpressed on the surface of hematopoietic stem cells of acute myeloid leukemia patients [90]. IL1RAP gene polymorphisms are associated with persistent hepatitis B virus infection [91].

**Reference proteins**

IL1RAP is in PDB.

## IMP-2

IMP2, also known as IGF2BP2, was reported to have tissue-specific expression in colon cancer [92]. IMP2 was also suggested to be a ost-transcriptional drivers of cancer progression [93].

**Reference proteins**
IMP2 was modeled using
RAVER1 by FAMS
[$p = 5 \times 10^{-38}$, 3-241(599), 23-258(280), 15%]
HNRPL by phyre2
[Conf. 100, 3-158(599), 5-206(209), 18%]
There are no reports that suggest relationship netween RAVER1, HNRPL, and cancer, yet.

## IREB2

The minor allele of a variant representing one of the two loci at 15q25 (rs2036534) was associated with increased IREB2 expression in two studies [94].
**Reference proteins**
IREB2 was modeled using IRP1 by;
FAMS
[$p = 0$, 7-963 (963), 1-887(888), 55%]
and
phyre2
[Conf. 100, 7-963(963),1-887(888), 62 %].
Dysregulation of IRP1-mediated iron metabolism causes gamma ray-specific radioresistance in leukemia cells [95]. IRP1 was reported to be under-expressed in prostate cancer [96].

## KCNN3

KCNN3 is also termed as SK3. New alkyl-lipid blockers of SK3 channels was reported to reduce cancer cell migration and occurrence of metastasis [97].
**Reference proteins**
KCNN3 was modeled using KCNA2 by ;
FAMS
[ $p = 1{\times}10^{-144}$,175-558 (736), 23-388 (389), 14%]
and
phyre2
[ Conf. 99.9, 316-558(736), 110-362(363), 20%]
KCNA2, also termed as kv1.2, has not yet any reports that suggest the direct interaction between KCNA2 and cancer. However, KCNA2 is suggested to be related to proliferation [98]. Thus, it is plausible that KCNA2 has potential roles in cancer.

## K6HF

Although there were no known reports that suggest relationship K6HF and cancers/tumors, K6HF was known to be related to pachyonychia congenita that is a rare hereditary disorder that can affect the nails, skin, mouth, larynx, hair, and eyes [99].
**Reference proteins**
K6HF was modeled using bacterial colicin ia by FAMS and phyre2. This may demonstrate that the selection of K6HF as a cancer-related-gene is erroneous.

## KLHL6

KLHL6 was known to be mutated in Chronic lymphocytic leukaemia [100]. KLHL6 was reported to be a putative oncogene in Diffuse Large B-cell Lymphoma [101].
**Reference proteins**
KLHL6 was modeled using KLHL2 by;
FAMS
[$p = 9 \times 10^{-86}$, 321-606(621), 2-286(286), 23%]
and
phyre2
[Conf.100, 318-606(621), 1-286(186), 26%]
KLHL2 (Mayven) was also reported to be related to cancer [102].

## LPP

LPP was reported to promote epithelial-to-mesenchymal transition in endometrial carcinomas cooperatively with ETV5 [103]. A complex containing LPP and $\alpha$-actinin mediates TGF$\beta$-induced migration and invasion of ErbB2-expressing breast cancer cells [104].
**Reference proteins**
LPP was modeled by FAMS using bacterial protein as reference protein, thus discarded. Phyre2 modeled LPP using LDB1 [Conf. 100, 412-557(612),1-153(157), 28 %]. LDB1 was reported to be essential cofactor to cancer oncogene LMO4 [105].

## MAFB

A novel molecular mechanism involved in multiple myeloma development revealed by targeting MAFB to haematopoietic progenitors was proposed [106]. Reexpression of oncoprotein MAFB in proliferative β-cells and Men1 insulinomas in mouse was also reported [107].
**Reference proteins**
MAFB was in PDB, although only less than half of length [ 214-303(323)] has predicted structure. Many other MAF family proteins were used as reference proteins.

## MCF2L2

MCF2L2 was reported to have higher CNV in adenocarcinoma [108]. CNV in MCF2L2 is even higher in squamous cell carcinoma patients. than in adenocarcinoma patients [109].

**Reference proteins**

MCF2L2 was modeled using VAV1 by;
FAMS
[ $p = 1 \times 10^{-129}$, 431-1005(1114), 2-533(541), 13%]
and
phyre2
[ Conf. 100, 612-917(1114), 166-443(541), 21%]
VAV1 was known to fine tune p53 control of apoptosis versus proliferation in breast cancer [110].

## MGC26647

There were no known reports that suggest relationship MGC26647, also known as C7orf62, and cancers/tumors,

**Reference proteins**

MGC26647 was model with appa by;
FAMS
[$p = 6 \times 10^{-26}$, 52-170(253), 5-113(114), 15%]
and
phyre2
[ Conf. 98.3, 49-147(253), 2-89(114), 18%]
Although appa is bactrial protein, appa was also known to be homologues to APP [111], amyloid protein precursor, which was known to correlates with early onset of Alzheimer's disease in humans. APP is known to be related to cancer. For example, suppression of APP expression could promote ovarian cancer cell proliferation and invasion [112].

## MYEOV

Amplification of MYEOV was involved in the Wnt and fibroblast growth factor signaling pathways, suggests that these pathways may be activated in esophageal carcinoma cell lines [30]. SNP in MYEOV was reported to be relates to prostate cancer risk [113]. Aberrations of MYEOV was reported in neuroblastoma [114].

**Reference proteins**

Phyre2 and FAMS failed to predict MYEOV protein structure.

## NOP5/NOP58

NOP58, also known as nucleolar protein 5, was were down regulated in tumor [115].

**Reference proteins**

NOP58 was modeled using many of NOP proteins as reference proteins. For example, NOP58 was modeled using NOP56 by ;
FAMS
[ $p = 1 \times 10^{-148}$, 28-402(529), 2-366(366), 29%]
and
phyre2
[ Conf. 100, 3-399(529),1-350(350), 31%]

## NRXN1

Genomic structural variation of NRXN1 was suggested to be related to cancer [116]. MRXN1 was also reported to be one of hepatitis B virus targeted genes [117].

**Reference proteins**

NRXN1 was in PDB.

## NUAK1

High NUAK1, also known as ARK5, expression correlates with poor prognosis and involved in nonsmall cell lung cancer cells migration and invasion [118]. NUAK1 promotes glioma cell invasion, and its elevated expression is correlated with poor clinical outcome [119].

**Reference proteins**

NUAK1 was modeled using ;
MARK3 by FAMS
[ $p = 1 \times 10^{-138}$, 43-370(661), 2-322(322), 40%]
and
PRKCB by phyre2
[ Conf. 100, 53-322(661),194-469(520), 28%]
MARKL1, homologous to MARK3, was reported to be elevated in hepatocellular carcinogenesis [120]. PRKCB was reported to be a disease-specific druggable target for treatment of Ewing sarcoma [121].

## NXPH1

Median methylation levels of NXPH1 in tumors was $\geq 30\%$ higher than in normal samples [122]. NXPH1 was also significantly more likely to be methylated in were significantly more likely to be methylated in IPMNs with high-grade than with low-grade dysplasias with high-grade than with low-grade dysplasia [123].

**Reference proteins**
NXPH1 was model using;
FLNB by FAMS
[ $p = 3 \times 10^{-22}$, 65-192(271), 1-101(108), 12%]
TRIM45 by phyre2
[ Conf. 95.5, 120-167(271), 46-95(113), 17%]
FLNB was reported to be a biomarker of hepatocellular carcinoma [124]. TRIM proteins are generally related to cancer [125].

## PACS1

PACS1 was reported to be dysregulated in cancer [126].
**Reference proteins**
FAMS could not find feasible struvutres of PACS1. PACS1 was modeled by phyre2 using yegS [ Conf. 96.1, 563-674(963),1-100(265), 17%], but yegS belongs to E. coli, thus there are no reports that suggest the relationship between yegS and cancer.

## PDK4

PDK4 was reported to play critical roles in tumor metastasis [127].
**Reference proteins**
PDK4 was in PDB.

## PIGO

PIGO was not reported to be related to cancer.
**Reference proteins**
PIGO was modeled using ENPP2 by;
FAMS
[ $p = 7 \times 10^{-77}$ ,46-408 (1089), 95-434(805),14%]
and
phyre2
[ Conf. 99.9, 52-350(1089), 90-467(783), 23%]
Meta-analysis of 8q24 for seven cancers reveals a locus between NOV and ENPP2 associated with cancer development [128].

## PIWIL1

PIWIL1-4 was reported to be significantly higher in tumorous tissue than in adjacent tissue [129].
**Reference proteins**
PIWIL1 was modeled using AGO2 by;
FAMS (
[$p = 0$, 97-860(861), 2-792(793), 21%]
and
phyre2
[ Conf. 100, 99-860(861), 4-792(795), 24%]
There were no studies that report the relationship between AGO2 and cancer. However, PIWIL1 belongs to AGO protein, thus it is natural that AGO2 was used for reference protein.

## PKP4

PKP4, also known as p0071, was associated with tumor growth, demonstrating an inverse correlation with tumor size [130]. PKP4 was suggested to have potentials for diagnosis of renal tumours [131].
**Reference proteins**
PKP4 was modeled using
PG by FAMS
[ $p = 1 \times 10^{-109}$, 510-1054(1192), 1-542(546),16%]
PKP1 by phyre2
[ Conf. 100, 529-1001(1192), 3-420(420), 37%]
Mutations in the PG and/or changes in its protein levels have been observed in various disease states, including cancer progression or cardiovascular defects [132]. The high specificity of membrane staining for PKP1 was observed in non-small-cell lung cancer [133].

## PLA2G4A

Zhang et al [134] found that PLA2G4A were upregulated in gastric cancer. PLA2G4A mutants were reported to modify protective effect of tea consumption against colorectal cancer [135]. Thus, even if PLA2G4A exhibit genotype-specific promoter methylation, it is not strange.
**Reference proteins**
Structure was reported in PDB.

## PLUNC

PLUNC was reported to be an auxiliary tool for the diagnosis of high-grade mucoepidermoid carcinoma of the salivary gland [136].
**Reference proteins**
PLUNC was modeled using BPI by;
FAMS
[ $p = 1 \times 10^{-58}$, 94-245(256), 42-196(217), 13%]
and
phyre2
[ Conf. 99.8, 103-238(256), 51-190(217), 16%]

The relation between BPI and inflammatory mediator tumor necrosis factor was reported [137].

## PPFIA1

PPFIA1 in laryngeal squamous cell carcinoma was reported to have higher expression levels in comparison to healthy controls [31]. PPFIA1 and CCND1, which was also identified by us, are frequently coamplified in breast cancer [138].

**Reference proteins**

PPFIA1 was modeled using Liprin-alpha2 (PPFIA2) by ;
FAMS
[$p = 1 \times 10^{-138}$,850-1120 (1202),1-265(265),85%]
and
phyre2
[ Conf. 100, 851-1120(1202), 1-261(261), 88%]
PPFIA2 gene was reported to be downregulated by androgens in the human prostate cancer cell line [139].

## PTPRT

PTPRT was reported to be tumour suppressor [140].

**Reference proteins**

PTPRT was modeled using R-PTP-S by;
FAMS
[$p = 0$, 863-1440 (1441), 1-568 (568), 41%]
and
phyre2
[Conf. 100, 863-1440(1441), 1-568(568), 43%]
RPTPS was suggested to play critical roles in human disease including cancer [141].

## PVRL3

PVRL3 was identified as Myc-bound loci in medulloblastoma cells [142]. Slight CNV of PVRL3 was observed in renal cell carcinomas [143].

**Reference proteins**

PVRL3 was in PDB.

## RAI17(ZMIZ1)

There are huge number of reports that ZMIZ1 regulate cancers [144, 145]. There are also reports that SNP of ZMIZ1 is associated with cancer [146]. Thus, it is likely that ZMIZ1 exhibits genotype-specific promoter methylaiton in cancer.

**Reference proteins**

ZMIZ1 was modeled using PIAS2 by;
FAMS
[$p = 8 \times 10^{-86}$, 603-824(1067), 58-261(261), 26%]
and
phyre2
[Conf. 100, 563-824(1067), 6-261(261), 29 %]
Similarity of ZMIZ1 with PIAS protein was already pointed out [147, 148].

## RAP2B

RAP2B is overexpressed in many types of tumors [149, 150].

**Reference proteins**

RAP2B was modeled using RAP2A by;
FAMS
[ $p = 2 \times 10^{-53}$,1-164(183), 1-164(167), 93%]
and
phyre2
[Conf. 100, 1-167(183), 1-167(167), 93 %]
RAP2A was one of twelve genes associated with both HNF1b and prostate cancer risk [151].

## RAPGEF5

RAPGEF5 was identified as andidate genes involved in tumorigenesis, displaying a robust correlation between expression and genomic alterations [152]. RAPGEF5 was expressed at higher levels in the responding group to erlotinib therapy [153].

**Reference proteins**

RAPGEF5 was modeled using RAPGEF4 by
FAMS
[ $p = 0$ ,15-579(580), 392-922(922),40%]
and
phyre2
[Conf. 100, 47-579(580),421-922(922), 47 %]
RAPGEF4 is also known as EPAC2. Anthrax edema toxin inhibits endothelial cell chemotaxis via Epac and Rap1 [154].

## RND3

Suppression of RND3 promotes cancer cell proliferation and invasion [155]. RND3 [156, 157] is a multifunctional protein and has several interaction with effectors [158]. It also plays critical roles in cancer formations, too [159, 160]. Since mutation of RND3 effector region was reported to attenuate RhoE-mediated disruption of the actin cytoskeleton,

indicating that RhoE exerts its inhibitory effects on ROCK-I through protein(s) binding to its effector region, it is likely reasonable that genotype-specific promoter methylation exists in RND3. Structure was reported in PDB.
**Reference proteins**
RND3 was in PDB.

## RPL14

RPL14 was reported to be increased in the presence of hepatocellular carcinoma (HCC) [161]. Alteration of RPL14 as reported in in squamous cell carcinomas and preneoplastic lesions of the esophagus [162].
**Reference proteins**
RPL14 was modeled with bacterial proteins, thus useless.

## SMAD3

SMAD3 was reported to be expressive in prostate cancer [163].
**Reference proteins**
SMAD3 was modeled by SMAD2 by;
FAMS
[$p = 1 \times 10^{-121}$, 223-422(425), 1-200(201), 97%]
and
phyre2
[ Conf. 100, 223-422(425), 1-200(201), 97%]
SMAD2 was reported to be tumor suppressor [164].

## SAMD12

Genomic rearrangement including SAMD12 was found in breast cancer cell line HCC1954 [165, 166].
**Reference proteins**
SAMD12 was modeled using
PPFIA2 by FAMS
[$p = 1 \times 10^{-28}$, 46-196(201), 51-207(253), 15%]
and
FLI1 by FAMS
[$p = 5 \times 10^{-24}$, 50-162 (201), 14-126(128), 13%]
For the relationship between PPIFA2 and cancer, see PPIFA1. EWS-FLI-1 expression was reported to trigger a Ewing's Sarcoma initiation program in primary Human mesenchymal stem cells [167]. phyre2 modeled SAMD12 using many sam domains. It is natural since SAMD12 means "SAM Domain-Containing Protein 12".

## SEMA3C

SEMA3C is involved in the progression of gastric cancer [168]. The influence of neuropilin-1 silencing on semaphorin 3A and 3C activity was reported in B16(F10) murine melanoma cells [169].
**Reference proteins**
SEMA3C was modeled using SEMA4D by;
FAMS
[ $p = 0$, 25-657(751), 3-611(621),32%]
and
phyre2
[Conf. 100, 24-661(751),2-619(621), 36%]
SEMA4D is tumor suppressor [170].

## SEMA3E

SEMA3E inhibits the development of tumors from glioblastoma cells implanted in the cortex of the brain [171].
**Reference proteins**
SEMA3E was modeled using SEMA4D as reference by
FAMS
[ $p = 1 \times 10^{-167}$, 28-668(775), 2-611(621), 31%]
and
phyre2
[Conf. 100, 27-678 (775), 1-621(621), 35%]
SEMA3E and SEMA4D were often investigated together and turned out to have similar outcomes [172, 173]. These two were investigated from the point of tumor progression/suppression. Generally, they play a potential roles in tumor formations.

## SENP2

SENP2 regulates hepatocellular carcinoma cell growth by modulating the stability of β-catenin [174]. SENP2 deSUMOylating enzyme reverses SUMO-modification of CHFR tumor suppressor, while SUMOylation negatively regulates the stability of CHFR tumor suppressor [175].
**Reference proteins**
SENP2 was in PDB.

## SFRS10

SFRS10, also known as TRA2B, has protein nuclear levels were elevated in poorly differentiated ($p = 0.044$) and lymph node metastases ($p = 0.003$) cancers [176]. Nuclear SFRS10 was reported to be significant in cervical cancer [177].

**Reference proteins**

SFRS10 was modeled using PSPC1 by;
FAMS as reference protein

[ $p = 2 \times 10^{-30}$, 29-261(288), 27-230(255), 17%]
and
phyre2

[Conf. 99.8, 117-198 (288), 8-83(239) 33%]
Knockdown of lncRNA LSINCT5 over expressed in breast and ovarian cancer reduces PSPC1 [178].

## STAT4

Genetic variants in STAT4 confers risk of hepatitis B virus-related hepatocellular carcinoma [179]. STAT proteins are reported to be novel cancer drug targets [180].

**Reference proteins**

STAT4 was modeled using STAT1 by;
FAMS

[$p = 0$, 2-676(748), 1-652(653), 54%]
and
phyre2

[Conf. 100, 2-677(748),1-653(653), 56%]
STAT1 was known to be tumor promoter [181].

## STMN2

STMN2 is a novel target of beta-catenin/TCF-mediated transcription in human hepatoma cells [182]. Kidins220-deficient SH-SY5Y cells and native SH-EP1 cells demonstrate down-regulation of the genes DCX and STMN2 [183]

**Reference proteins**

STMN2 was modeled using STMN4 by;
FAMS

[ $p = 2 \times 10^{-29}$, 39-178(179), 2-135(136),67%]
and
phyre2

[Conf. 99.4, 34-174(179), 2-123(124), 78%]
Importance of STMN4 during neuroblastoma differentiation was reported [184].

## STXBP6

STXBP6, also termed as amisyn, was known to be a syntaxin-binding protein that may regulate SNARE complex assembly [185] that play critical roles in membrane fusion. Membrane fusion is known to be related to metastasis [186].

**Reference proteins**

STXBP6 was modeled using VAMP8 by FAMS;

[$p = 1 \times 10^{-28}$, 81-178(210), 174-280(280), 23%].
and
sec3 by phyre2;

[Conf. 20-167(210), 37-181(185), 16%]
Since VAMP8 is a member of SNARE complex, it is reasonable that STXBP6 was modeled using VAMP8. EXOC1, human homolog of sec3, was reported to be one of six genes mapped in chromosome 4 that showed the greatest number of positive and negative regulation between samples [187].

## TGFB2

TGFB2 is famous as potential tumor regulator [188].

**Reference proteins**

TGFB2 was modeled using TGFB1 by;
FAMS

[ $p = 2 \times 10^{-77}$, 21-414(414), 3-343(343), 45%]
and phyre2

[ Conf. 100, 20-414(414), 1-342(342), 47 %]
TGFB1 was reported to be clinically important for cancer [189].

## TMEM16A

TMEM16A induces MAPK and contributes directly to tumorigenesis and cancer progression [190]. TMEM16A was one of 11 genes in 11q13 region exclusively in patients with cervical Lymph node metastasis [191].

**Reference proteins**

Neither FAMS nor phyre2 predicted reliable protein structures for TMEM16A.

## TOMM7

TOMM7 was upregulated in the cell line of L9981 after transfected with NM23-H1 gene [192].

**Reference proteins**

Neither FAMS nor phyre2 predicted reliable protein structures for TOMM7.

# UBP1

There are no studies that report the relationship with cancer.

**Reference protein**

UBP1 was modeled using CG3027-PA by;
FAMS

[ $p = 1 \times 10^{-143}$, 5-384(384),1-347(348),56%]

and
phyre2
[Conf. 100, 6-384(384),1-380(381), 64%]
Since CG3027 is fly protein, there are no studies that report the relation with cancer.

### ZA20D2

There are no studies that report the relationship with cancer.
**Reference proteins**
ZA20D2 was in PDB.

### ZBED2

ZBED2 has no reports that suggest relationship to cancers/tumors.
**Reference proteins**
ZBED2 was in PDB.

### ZBTB20

ZBTB20 was reported to be located in one of two two new susceptibility loci for non-cardia gastric cancer [193].
**Reference proteins**
There are two many short regions modeled by distinct reference proteins (e.e., POZ domain and Zinc finger proteins) with smaller sequence identity (less than 50%) using either FAMS or phyre2, we cannot identify representative reference proteins that model ZBTB20.

### ZFPM2

ZFPM2, also known as FOG2, expression in pediatric ovarian sex cord-stromal tumors replicates embryonal gonadal phenotype [194]. Microdeletions were present in ZFPM2 of adult Wilms' tumor [195].
**Reference proteins**
ZFPM2 was modeled using;
<u>GTF3A</u> by FAMS
[$p = 2 \times 10^{-29}$, 546-729(1151), 5-172(182) 11%]
<u>ZNF297B</u> by phyre2
[ Conf. 99.8, 242-380(1151), 3-109(110), 21%]
The predicted structure is too short compared with total length thus useless.

### ZNF138

ZNF138 was reported as a putative candidate genes for developmental and malignant disorders [196].
**Reference proteins**
ZNF138 was modeled using;
<u>AAAT</u> by FAMS
[ $p = 2 \times 10^{-62}$, 91-244(262), 1-151(151), 44%]
and
<u>PRDM9</u> by phyre2
[Conf. 100, 58-262(262), 4-201(215), 6%]
AAAT was proposed to be a inhibitor for pancreatic carcinoma [197]. PRDM9 was proposed to be a highly specific cancer biomarker genes [198].

### ZNF588

ZNF588, also known as ZNF107, was reported to have CNV alternation in complex diseases [199].
**Reference proteins**
ZNF138 was modeled using
<u>AAAT</u> by FAMS
[ $p = 2 \times 10^{-52}$, 85-238(783), 1-151(151) 48%]
and
<u>PRDM9</u> by phyre2
[ Conf. 100, 600-770(783), 1-212(215), 6%]
For the relationship between AAAT, PRDM9 and cancers, see ZNF138.

### ZNF639

ZNF639, also known as ZASC1, was reported to increase in recurrent oral carcinoma [200]. ZASC1 regulates murine leukemia virus transcription [201].
**Reference proteins**
ZNF138 was modeled using
<u>AAAT</u> by FAMS
[ $p = 1 \times 10^{-55}$, 242-396(485), 1-151(151), 21%]
and
<u>PRDM9</u> by phyre2
[ Conf. 100, 196-485(485), 1-215(215), 7%]
For the relationship between AAAT, PRDM9 and cancers, see ZNF138.

### raptor

RNA interference targeting raptor inhibits proliferation of gastric cancer cells [202]. Raptor was suggested to be related to tumor suppressors [203].
**Reference proteins**
raptor was modeled using

APAF1 by FAMS

[ $p = 9 \times 10^{-97}$,410-1318(1335), 243-1119 (1133), 10%]

DDB1 by phyre2

[Conf. 100, 1018-1331(1335), 87-402(402), 15%]

APAF1 was reported to be a target of miR-221 that is overexpressed in neoplasms [204]. DDB1 was reported to be downregulted in pancreatic carcinoma cells [205].

## References


1. La Madrid AM, Campbell N, Smith S, Cohn SL, Salgia R: **Targeting ALK: a promising strategy for the treatment of non-small cell lung cancer, non-Hodgkin's lymphoma, and neuroblastoma**. *Target Oncol* 2012, **7**(3):199–210.
2. Chung AS, Guan YJ, Yuan ZL, Albina JE, Chin YE: **Ankyrin repeat and SOCS box 3 (ASB3) mediates ubiquitination and degradation of tumor necrosis factor receptor II**. *Mol. Cell. Biol.* 2005, **25**(11):4716–4726.
3. Ukkola LT, Onkamo P, Raijas P, Karma K, Jarvela I: **Musical aptitude is associated with AVPR1A-haplotypes**. *PLoS ONE* 2009, **4**(5):e5534.
4. Wassink TH, Piven J, Vieland VJ, Pietila J, Goedken RJ, Folstein SE, Sheffield VC: **Examination of AVPR1a as an autism susceptibility gene**. *Mol. Psychiatry* 2004, **9**(10):968–972.
5. Qu?c Lu'o'ng KV, Nguy?n LT: **The roles of beta-adrenergic receptors in tumorigenesis and the possible use of beta-adrenergic blockers for cancer treatment: possible genetic and cell-signaling mechanisms**. *Cancer Manag Res* 2012, **4**:431–445.
6. Barron TI, Sharp L, Visvanathan K: **Beta-adrenergic blocking drugs in breast cancer: a perspective review**. *Ther Adv Med Oncol* 2012, **4**(3):113–125.
7. Shi M, Liu D, Duan H, Qian L, Wang L, Niu L, Zhang H, Yong Z, Gong Z, Song L, Yu M, Hu M, Xia Q, Shen B, Guo N: **The $\beta$2-adrenergic receptor and Her2 comprise a positive feedback loop in human breast cancer cells**. *Breast Cancer Res. Treat.* 2011, **125**(2):351–362.
8. Feng F, Yang J, Tong L, Yuan S, Tian Y, Hong L, Wang W, Zhang H: **Substance P immunoreactive nerve fibres are related to gastric cancer differentiation status and could promote proliferation and migration of gastric cancer cells**. *Cell Biol. Int.* 2011, **35**(6):623–629.
9. Igarashi Y, Kannagi R: **Glycosphingolipids as mediators of phenotypic changes associated with development and cancer progression**. *J. Biochem.* 2010, **147**:3–8.
10. Han Y, Tu WW, Wen YG, Li DP, Qiu GQ, Tang HM, Peng ZH, Zhou CZ: **Identification and validation that up-expression of HOXA13 is a novel independent prognostic marker of a worse outcome in gastric cancer based on immunohistochemistry**. *Med. Oncol.* 2013, **30**(2):564.
11. Park JY, Park KH, Bang S, Kim MH, Lee JE, Gang J, Koh SS, Song SY: **CXCL5 overexpression is associated with late stage gastric cancer**. *J. Cancer Res. Clin. Oncol.* 2007, **133**(11):835–840.
12. Van Etten RA: **BCL6: a novel target for therapy of Ph+ B cell acute lymphoblastic leukemia**. *Cancer Cell* 2011, **20**:3–5.
13. Cerchietti LC, Ghetu AF, Zhu X, Da Silva GF, Zhong S, Matthews M, Bunting KL, Polo JM, Fares C, Arrowsmith CH, Yang SN, Garcia M, Coop A, Mackerell AD, Prive GG, Melnick A: **A small-molecule inhibitor of BCL6 kills DLBCL cells in vitro and in vivo**. *Cancer Cell* 2010, **17**(4):400–411.
14. Logarajah S, Hunter P, Kraman M, Steele D, Lakhani S, Bobrow L, Venkitaraman A, Wagner S: **BCL-6 is expressed in breast cancer and prevents mammary epithelial differentiation**. *Oncogene* 2003, **22**(36):5572–5578.
15. Cabarcas S, Jacob J, Veras I, Schramm L: **Differential expression of the TFIIIB subunits Brf1 and Brf2 in cancer cells**. *BMC Mol. Biol.* 2008, **9**:74.
16. Lu M, Tian H, Yue W, Li L, Li S, Qi L, Hu W, Gao C, Si L: **Overexpression of TFIIB-related factor 2 is significantly correlated with tumor angiogenesis and poor survival in patients with esophageal squamous cell cancer**. *Med. Oncol.* 2013, **30**(2):553.
17. Tian E, Ten Hagen KG: **Recent insights into the biological roles of mucin-type O-glycosylation**. *Glycoconj. J.* 2009, **26**(3):325–334.
18. Brockhausen I: **Mucin-type O-glycans in human colon and breast cancer: glycodynamics and functions**. *EMBO Rep.* 2006, **7**(6):599–604.
19. Chung W, Bondaruk J, Jelinek J, Lotan Y, Liang S, Czerniak B, Issa JP: **Detection of bladder cancer using novel DNA methylation biomarkers in urine sediments**. *Cancer Epidemiol. Biomarkers Prev.* 2011, **20**(7):1483–1491.
20. McDonald PC, Winum JY, Supuran CT, Dedhar S: **Recent developments in targeting carbonic anhydrase IX for cancer therapeutics**. *Oncotarget* 2012, **3**:84–97.
21. Takakura M, Yokomizo A, Tanaka Y, Kobayashi M, Jung G, Banno M, Sakuma T, Imada K, Oda Y, Kamita M, Honda K, Yamada T, Naito S, Ono M: **Carbonic anhydrase I as a new plasma biomarker for prostate cancer**. *ISRN Oncol* 2012, **2012**:768190.
22. Scozzafava A, Supuran CT, Carta F: **Antiobesity carbonic anhydrase inhibitors: a literature and patent review**. *Expert Opin Ther Pat* 2013, **23**(6):725–735.
23. Bergamaschi A, Kim YH, Kwei KA, La Choi Y, Bocanegra M, Langer?d A, Han W, Noh DY, Huntsman DG, Jeffrey SS, B?rresen-Dale AL, Pollack JR: **CAMK1D amplification implicated in epithelial-mesenchymal transition in basal-like breast cancer**. *Mol Oncol* 2008, **2**(4):327–339.
24. Gaudet MM, Kuchenbaecker KB, Vijai J, Klein RJ, Kirchhoff T, McGuffog L, Barrowdale D, Dunning AM, Lee A, Dennis J, Healey S, Dicks E, Soucy P, Sinilnikova OM, Pankratz VS, Wang X, Eldridge RC, Tessier DC, Vincent D, Bacot F, Hogervorst FB, Peock S, Stoppa-Lyonnet D, Peterlongo P, Schmutzler RK, Nathanson KL, Piedmonte M, Singer CF, Thomassen M, Hansen Tv, Neuhausen SL, Blanco I, Greene MH, Garber J, Weitzel JN, Andrulis IL, Goldgar DE, D'Andrea E, Caldes T, Nevanlinna H, Osorio A, van Rensburg EJ, Arason A, Rennert G, van den Ouweland AM, van der Hout AH, Kets CM, Aalfs CM, Wijnen JT, Ausems MG, Frost D, Ellis S, Fineberg E, Platte R, Evans DG, Jacobs C, Adlard J, Tischkowitz M, Porteous ME, Damiola F, Golmard L, Barjhoux L, Longy M, Belotti M, Ferrer SF, Mazoyer S, Spurdle AB, Manoukian S, Barile M, Genuardi M, Arnold N, Meindl A, Sutter C, Wappenschmidt B, Domchek SM, Pfeiler G, Friedman E,

Jensen UB, Robson M, Shah S, Lazaro C, Mai PL, Benitez J, Southey MC, Schmidt MK, Fasching PA, Peto J, Humphreys MK, Wang Q, Michailidou K, Sawyer EJ, Burwinkel B, Guenel P, Bojesen SE, Milne RL, Brenner H, Lochmann M, Aittomaki K, Dork T, Margolin S, Mannermaa A, Lambrechts D, Chang-Claude J, Radice P, Giles GG, Haiman CA, Winqvist R, Devillee P, Garcia-Closas M, Schoof N, Hooning MJ, Cox A, Pharoah PD, Jakubowska A, Orr N, Gonzalez-Neira A, Pita G, Alonso MR, Hall P, Couch FJ, Simard J, Altshuler D, Easton DF, Chenevix-Trench G, Antoniou AC, Offit K: **Identification of a BRCA2-specific modifier locus at 6p24 related to breast cancer risk**. *PLoS Genet.* 2013, **9**(3):e1003173.

25. Makishima H, Jankowska AM, McDevitt MA, O'Keefe C, Dujardin S, Cazzolli H, Przychodzen B, Prince C, Nicoll J, Siddaiah H, Shaik M, Szpurka H, Hsi E, Advani A, Paquette R, Maciejewski JP: **CBL, CBLB, TET2, ASXL1, and IDH1/2 mutations and additional chromosomal aberrations constitute molecular events in chronic myelogenous leukemia**. *Blood* 2011, **117**(21):198–206.

26. Loeser S, Loser K, Bijker MS, Rangachari M, van der Burg SH, Wada T, Beissert S, Melief CJ, Penninger JM: **Spontaneous tumor rejection by cbl-b-deficient CD8+ T cells**. *J. Exp. Med.* 2007, **204**(4):879–891.

27. Horwitz M, Goode EL, Jarvik GP: **Anticipation in familial leukemia**. *Am. J. Hum. Genet.* 1996, **59**(5):990–998.

28. Fang Y, Cao Z, Hou Q, Ma C, Yao C, Li J, Wu XR, Huang C: **Cyclin D1 Downregulation Contributes to Anti-Cancer Effect of Isorhapontigenin (ISO) on Human Bladder Cancer Cells**. *Mol. Cancer Ther.* 2013.

29. Reis-Filho JS, Savage K, Lambros MB, James M, Steele D, Jones RL, Dowsett M: **Cyclin D1 protein overexpression and CCND1 amplification in breast carcinomas: an immunohistochemical and chromogenic in situ hybridisation analysis**. *Mod. Pathol.* 2006, **19**(7):999–1009.

30. Brown J, Bothma H, Veale R, Willem P: **Genomic imbalances in esophageal carcinoma cell lines involve Wnt pathway genes**. *World J. Gastroenterol.* 2011, **17**(24):2909–2923.

31. Jarmuz-Szymczak M, Pelinska K, Kostrzewska-Poczekaj M, Bembnista E, Giefing M, Brauze D, Szaumkessel M, Marszalek A, Janiszewska J, Kiwerska K, Bartochowska A, Grenman R, Szyfter W, Szyfter K: **Heterogeneity of 11q13 region rearrangements in laryngeal squamous cell carcinoma analyzed by microarray platforms and fluorescence in situ hybridization**. *Mol. Biol. Rep.* 2013, **40**(7):4161–4171.

32. Liu X, Lv XB, Wang XP, Sang Y, Xu S, Hu K, Wu M, Liang Y, Liu P, Tang J, Lu WH, Feng QS, Chen LZ, Qian CN, Bei JX, Kang T, Zeng YX: **MiR-138 suppressed nasopharyngeal carcinoma growth and tumorigenesis by targeting the CCND1 oncogene**. *Cell Cycle* 2012, **11**(13):2495–2506.

33. Wang W, Zhao LJ, Tan YX, Ren H, Qi ZT: **Identification of deregulated miRNAs and their targets in hepatitis B virus-associated hepatocellular carcinoma**. *World J. Gastroenterol.* 2012, **18**(38):5442–5453.

34. Qiu S, Huang D, Li F, Yin D, Kung HF, Peng Y: **Suppression of tumorigenicity by MicroRNA-138 through inhibition of EZH2-CDK4/6-pRb-E2F1 signal loop in glioblastoma multiforme**. *Biochim. Biophys. Acta* 2013.

35. Muller D, Millon R, Theobald S, Hussenet T, Wasylyk B, du Manoir S, Abecassis J: **Cyclin L1 (CCNL1) gene alterations in human head and neck squamous cell carcinoma**. *Br. J. Cancer* 2006, **94**(7):1041–1044.

36. Marsaud V, Tchakarska G, Andrieux G, Liu JM, Dembele D, Jost B, Wdzieczak-Bakala J, Renoir JM, Sola B: **Cyclin K and cyclin D1b are oncogenic in myeloma cells**. *Mol. Cancer* 2010, **9**:103.

37. DeFilippis RA, Chang H, Dumont N, Rabban JT, Chen YY, Fontenay GV, Berman HK, Gauthier ML, Zhao J, Hu D, Marx JJ, Tjoe JA, Ziv E, Febbraio M, Kerlikowske K, Parvin B, Tlsty TD: **CD36 repression activates a multicellular stromal program shared by high mammographic density and tumor tissues**. *Cancer Discov* 2012, **2**(9):826–839.

38. Koch M, Hussein F, Woeste A, Grundker C, Frontzek K, Emons G, Hawighorst T: **CD36-mediated activation of endothelial cell apoptosis by an N-terminal recombinant fragment of thrombospondin-2 inhibits breast cancer growth and metastasis in vivo**. *Breast Cancer Res. Treat.* 2011, **128**(2):337–346.

39. Hosen N, Park CY, Tatsumi N, Oji Y, Sugiyama H, Gramatzki M, Krensky AM, Weissman IL: **CD96 is a leukemic stem cell-specific marker in human acute myeloid leukemia**. *Proc. Natl. Acad. Sci. U.S.A.* 2007, **104**(26):11008–11013.

40. Becker H, Marcucci G, Maharry K, Radmacher MD, Mrozek K, Margeson D, Whitman SP, Paschka P, Holland KB, Schwind S, Wu YZ, Powell BL, Carter TH, Kolitz JE, Wetzler M, Carroll AJ, Baer MR, Moore JO, Caligiuri MA, Larson RA, Bloomfield CD: **Mutations of the Wilms tumor 1 gene (WT1) in older patients with primary cytogenetically normal acute myeloid leukemia: a Cancer and Leukemia Group B study**. *Blood* 2010, **116**(5):788–792.

41. Balakrishnan A, Bleeker FE, Lamba S, Rodolfo M, Daniotti M, Scarpa A, van Tilborg AA, Leenstra S, Zanon C, Bardelli A: **Novel somatic and germline mutations in cancer candidate genes in glioblastoma, melanoma, and pancreatic carcinoma**. *Cancer Res.* 2007, **67**(8):3545–3550.

42. Kadaba R, Birke H, Wang J, Hooper S, Andl CD, Di Maggio F, Soylu E, Ghallab M, Bor D, Froeling FE, Bhattacharya S, Rustgi AK, Sahai E, Chelala C, Sasieni P, Kocher HM: **Imbalance of desmoplastic stromal cell numbers drives aggressive cancer processes**. *J. Pathol.* 2013, **230**:107–117.

43. Shahjee HM, Koch KR, Guo L, Zhang CO, Keay SK: **Antiproliferative factor decreases Akt phosphorylation and alters gene expression via CKAP4 in T24 bladder carcinoma cells**. *J. Exp. Clin. Cancer Res.* 2010, **29**:160.

44. Zhen ZP, Zhang J, Zhang SY: **Development of a novel small antibody that retains specificity for tumor targeting**. *J. Exp. Clin. Cancer Res.* 2009, **28**:59.

45. Zhang Z, Furge KA, Yang XJ, Teh BT, Hansel DE: **Comparative gene expression profiling analysis of urothelial carcinoma of the renal pelvis and bladder**. *BMC Med Genomics* 2010, **3**:58.

46. Wang C, Rajput S, Watabe K, Liao DF, Cao D: **Acetyl-CoA carboxylase-a as a novel target for cancer therapy**. *Front Biosci (Schol Ed)* 2010, **2**:515–526.

47. Chile T, Correa-Giannella ML, Fortes MA, Bronstein MD, Cunha-Neto MB, Giannella-Neto D, Giorgi RR: **Expression of CRABP1, GRP, and RERG mRNA in clinically non-functioning and functioning pituitary adenomas**. *J. Endocrinol. Invest.* 2011, **34**(8):e214–218.

48. Miyake T, Ueda Y, Matsuzaki S, Miyatake T, Yoshino K, Fujita M, Nomura T, Enomoto T, Kimura T: **CRABP1-reduced expression is associated with poorer prognosis in serous and clear cell ovarian adenocarcinoma**. *J. Cancer Res. Clin. Oncol.* 2011, **137**(4):715–722.

49. Dong H, Zhang H, Liang J, Yan H, Chen Y, Shen Y, Kong Y, Wang S, Zhao G, Jin W: **Digital karyotyping reveals probable target genes at 7q21.3 locus in hepatocellular carcinoma**. *BMC Med Genomics* 2011, **4**:60.

50. Johnstone RW, Wang J, Tommerup N, Vissing H, Roberts T, Shi Y: **Ciao 1 is a novel WD40 protein that interacts with the tumor suppressor protein WT1**. *J. Biol. Chem.* 1998, **273**(18):10880–10887.

51. Wang R, Lohr CV, Fischer K, Dashwood WM, Greenwood JA, Ho E, Williams DE, Ashktorab H, Dashwood MR, Dashwood RH: **Epigenetic inactivation of endothelin-2 and endothelin-3 in colon cancer**. *Int. J. Cancer* 2013, **132**(5):1004–1012.

52. Bagnato A, Spinella F, Rosano L: **Emerging role of the endothelin axis in ovarian tumor progression**. *Endocr. Relat. Cancer* 2005, **12**(4):761–772.

53. Berry P, Burchill S: **Endothelins may modulate invasion and proliferation of Ewing's sarcoma and neuroblastoma**. *Clin. Sci.* 2002, **103 Suppl 48**:322S–326S.

54. Grimshaw MJ: **Endothelins in breast tumour cell invasion**. *Cancer Lett.* 2005, **222**(2):129–138.

55. Sciorra VA, Sanchez MA, Kunibe A, Wurmser AE: **Suppression of glioma progression by Egln3**. *PLoS ONE* 2012, **7**(8):e40053.

56. Hatzimichael E, Dasoula A, Shah R, Syed N, Papoudou-Bai A, Coley HM, Dranitsaris G, Bourantas KL, Stebbing J, Crook T: **The prolyl-hydroxylase EGLN3 and not EGLN1 is inactivated by methylation in plasma cell neoplasia**. *Eur. J. Haematol.* 2010, **84**:47–51.

57. Lopez-Jimenez E, Gomez-Lopez G, Leandro-Garcia LJ, Munoz I, Schiavi F, Montero-Conde C, de Cubas AA, Ramires R, Landa I, Leskela S, Maliszewska A, Inglada-Perez L, de la Vega L, Rodriguez-Antona C, Leton R, Bernal C, de Campos JM, Diez-Tascon C, Fraga MF, Boullosa C, Pisano DG, Opocher G, Robledo M, Cascon A: **Research resource: Transcriptional profiling reveals different pseudohypoxic signatures in SDHB and VHL-related pheochromocytomas**. *Mol. Endocrinol.* 2010, **24**(12):2382–2391.

58. Bozoky B, Savchenko A, Csermely P, Korcsmaros T, Dul Z, Ponten F, Szekely L, Klein G: **Novel signatures of cancer-associated fibroblasts**. *Int. J. Cancer* 2013, **133**(2):286–293.

59. Deng X, Cao Y, Liu Y, Li F, Sambandam K, Rajaraman S, Perkins AS, Fields AP, Hellmich MR, Townsend CM, Thompson EA, Ko TC: **Overexpression of Evi-1 oncoprotein represses TGF-$\beta$ signaling in colorectal cancer**. *Mol. Carcinog.* 2013, **52**(4):255–264.

60. Paquette RL, Nicoll J, Chalukya M, Elashoff D, Shah NP, Sawyers C, Spiteri E, Nanjangud G, Rao PN: **Frequent EVI1 translocations in myeloid blast crisis CML that evolves through tyrosine kinase inhibitors**. *Cancer Genet* 2011, **204**(7):392–397.

61. Osterholm C, Lu N, Liden A, Karlsen TV, Gullberg D, Reed RK, Kusche-Gullberg M: **Fibroblast EXT1-levels influence tumor cell proliferation and migration in composite spheroids**. *PLoS ONE* 2012, **7**(7):e41334.

62. Zhu HY, Hu YL, Yang Y, Wu X, Zhu RF, Zhu XY, Duan HL, Zhang Y, Zhou JY: **Mutation analysis and prenatal diagnosis of EXT1 gene mutations in Chinese patients with multiple osteochondromas**. *Chin. Med. J.* 2011, **124**(19):3054–3057.

63. Nadanaka S, Zhou S, Kagiyama S, Shoji N, Sugahara K, Sugihara K, Asano M, Kitagawa H: **EXTL2, a member of the EXT family of tumor suppressors, controls glycosaminoglycan biosynthesis in a xylose kinase-dependent manner**. *J. Biol. Chem.* 2013, **288**(13):9321–9333.

64. Zhang Y, Yu G, Jiang P, Xiang Y, Li W, Lee W, Zhang Y: **Decreased expression of protease-activated receptor 4 in human gastric cancer**. *Int. J. Biochem. Cell Biol.* 2011, **43**(9):1277–1283.

65. Ghio P, Cappia S, Selvaggi G, Novello S, Lausi P, Zecchina G, Papotti M, Borasio P, Scagliotti GV: **Prognostic role of protease-activated receptors 1 and 4 in resected stage IB non-small-cell lung cancer**. *Clin Lung Cancer* 2006, **7**(6):395–400.

66. Hernandez NA, Correa E, Avila EP, Vela TA, Perez VM: **PAR1 is selectively over expressed in high grade breast cancer patients: a cohort study**. *J Transl Med* 2009, **7**:47.

67. Ando S, Otani H, Yagi Y, Kawai K, Araki H, Fukuhara S, Inagaki C: **Proteinase-activated receptor 4 stimulation-induced epithelial-mesenchymal transition in alveolar epithelial cells**. *Respir. Res.* 2007, **8**:31.

68. Chen L, Su L, Li J, Zheng Y, Yu B, Yu Y, Yan M, Gu Q, Zhu Z, Liu B: **Hypermethylated FAM5C and MYLK in serum as diagnosis and pre-warning markers for gastric cancer**. *Dis. Markers* 2012, **32**(3):195–202.

69. Cimino Y, Costes A, Damotte D, Validire P, Mistou S, Cagnard N, Alifano M, Regnard JF, Chiocchia G, Sautes-Fridman C, Tourneur L: **FADD protein release mirrors the development and aggressiveness of human non-small cell lung cancer**. *Br. J. Cancer* 2012, **106**(12):1989–1996.

70. Schinske KA, Nyati S, Khan AP, Williams TM, Johnson TD, Ross BD, Tomas RP, Rehemtulla A: **A novel kinase inhibitor of FADD phosphorylation chemosensitizes through the inhibition of NF-*kappa*B**. *Mol. Cancer Ther.* 2011, **10**(10):1807–1817.

71. Volkomorov V, Grigoryeva E, Krasnov G, Litviakov N, Tsyganov M, Karbyshev M, Zavyalova M, Afanas?ev S, Cherdyntseva N, Lisitsyn N, Beresten S: **Search for potential gastric cancer markers using miRNA databases and gene expression analysis**. *Exp. Oncol.* 2013, **35**:2–7.

72. Khodarev NN, Yu J, Labay E, Darga T, Brown CK, Mauceri HJ, Yassari R, Gupta N, Weichselbaum RR: **Tumour-endothelium interactions in co-culture: coordinated changes of gene expression profiles and phenotypic properties of endothelial cells**. *J. Cell. Sci.* 2003, **116**(Pt 6):1013–1022.

73. Lian ZQ, Wang Q, Li WP, Zhang AQ, Wu L: **Screening of significantly hypermethylated genes in breast cancer using microarray-based methylated-CpG island recovery assay and identification of their expression levels**. *Int. J. Oncol.* 2012, **41**(2):629–638.

74. Feng S, Dakhova O, Creighton CJ, Ittmann M: **Endocrine fibroblast growth factor FGF19 promotes prostate cancer progression**. *Cancer Res.* 2013, **73**(8):2551–2562.

75. Latasa MU, Salis F, Urtasun R, Garcia-Irigoyen O, Elizalde M, Uriarte I, Santamaria M, Feo F, Pascale RM, Prieto J, Berasain C, Avila MA: **Regulation of amphiregulin gene expression by $\beta$-catenin signaling in human hepatocellular carcinoma cells: a novel crosstalk between FGF19 and the EGFR system**. *PLoS ONE* 2012, **7**(12):e52711.

76. Turner N, Grose R: **Fibroblast growth factor signalling: from development to cancer**. *Nat. Rev. Cancer* 2010, **10**(2):116–129.

77. Arao T, Ueshima K, Matsumoto K, Nagai T, Kimura H, Hagiwara S, Sakurai T, Haji S, Kanazawa A, Hidaka H, Iso Y, Kubota K, Shimada M, Utsunomiya T, Hirooka M, Hiasa Y, Toyoki Y, Hakamada K, Yasui K, Kumada T, Toyoda H, Sato S, Hisai H, Kuzuya T, Tsuchiya K, Izumi N, Arii S, Nishio K, Kudo M: **FGF3/FGF4 amplification and multiple lung metastases in responders to sorafenib in hepatocellular carcinoma**. *Hepatology* 2013, **57**(4):1407–1415.

78. Linehan WM, Rouault TA: **Molecular Pathways: Fumarate Hydratase-Deficient Kidney Cancer: Targeting the Warburg Effect in Cancer**. *Clin. Cancer Res.* 2013.

79. Ha YS, Chihara Y, Yoon HY, Kim YJ, Kim TH, Woo SH, Yun SJ, Kim IY, Hirao Y, Kim WJ: **Downregulation of fumarate hydratase is related to tumorigenesis in sporadic renal cell cancer**. *Urol. Int.* 2013, **90**(2):233–239.

80. Yang X, Vasudevan P, Parekh V, Penev A, Cunningham JM: **Bridging cancer biology with the clinic: relative expression of a GRHL2-mediated gene-set pair predicts breast cancer metastasis**. *PLoS ONE* 2013, **8**(2):e56195.

81. Xiang X, Deng Z, Zhuang X, Ju S, Mu J, Jiang H, Zhang L, Yan J, Miller D, Zhang HG: **Grhl2 determines the epithelial phenotype of breast cancers and promotes tumor progression**. *PLoS ONE* 2012, **7**(12):e50781.

82. Cheng L, Wang P, Yang S, Yang Y, Zhang Q, Zhang W, Xiao H, Gao H, Zhang Q: **Identification of genes with a correlation between copy number and expression in gastric cancer**. *BMC Med Genomics* 2012, **5**:14.

83. Oberoi P, Jabulowsky RA, Bahr-Mahmud H, Wels WS: **EGFR-targeted granzyme B expressed in NK cells enhances natural cytotoxicity and mediates specific killing of tumor cells**. *PLoS ONE* 2013, **8**(4):e61267.

84. Ji XJ, Chen SH, Zhu L, Pan H, Zhou Y, Li W, You WC, Gao CC, Zhu JH, Jiang K, Wang HD: **Knockdown of NF-E2-related factor 2 inhibits the proliferation and growth of U251MG human glioma cells in a mouse xenograft model**. *Oncol. Rep.* 2013, **30**:157–164.

85. Satoh H, Moriguchi T, Takai J, Ebina M, Yamamoto M: **Nrf2 prevents initiation but accelerates progression through the Kras signaling pathway during lung carcinogenesis**. *Cancer Res.* 2013.

86. Zhao M, Tang SN, Marsh JL, Shankar S, Srivastava RK: **Ellagic acid inhibits human pancreatic cancer growth in Balb c nude mice**. *Cancer Lett.* 2013.

87. Chou CH, Cheng YF, Siow TY, Kumar A, Peck K, Chang C: **SCUBE3 regulation of early lung cancer angiogenesis and metastatic progression**. *Clin. Exp. Metastasis* 2013.

88. Buffa L, Fuchs E, Pietropaolo M, Barr F, Solimena M: **ICA69 is a novel Rab2 effector regulating ER-Golgi trafficking in insulinoma cells**. *Eur. J. Cell Biol.* 2008, **87**(4):197–209.

89. Schulmann K, Mori Y, Croog V, Yin J, Olaru A, Sterian A, Sato F, Wang S, Xu Y, Deacu E, Berki AT, Hamilton JP, Kan T, Abraham JM, Schmiegel W, Harpaz N, Meltzer SJ: **Molecular phenotype of inflammatory bowel disease-associated neoplasms with microsatellite instability**. *Gastroenterology* 2005, **129**:74–85.

90. Barreyro L, Will B, Bartholdy B, Zhou L, Todorova TI, Stanley RF, Ben-Neriah S, Montagna C, Parekh S, Pellagatti A, Boultwood J, Paietta E, Ketterling RP, Cripe L, Fernandez HF, Greenberg PL, Tallman MS, Steidl C, Mitsiades CS, Verma A, Steidl U: **Overexpression of IL-1 receptor accessory protein in stem and progenitor cells and outcome correlation in AML and MDS**. *Blood* 2012, **120**(6):1290–1298.

91. Kim SS, Cheong JY, Lee D, Lee SK, Kim MH, Kwack K, Yang SJ, Lee HY, Cho SW: **Interleukin-1$\beta$and interleukin-1 receptor accessory protein gene polymorphisms are associated with persistent hepatitis B virus infection**. *Hepatogastroenterology* 2012, **59**(113):190–197.

92. Liu W, Li Z, Xu W, Wang Q, Yang S: **Humoral autoimmune response to IGF2 mRNA-binding protein (IMP2/p62) and its tissue-specific expression in colon cancer**. *Scand. J. Immunol.* 2013, **77**(4):255–260.

93. Bell JL, Wachter K, Muhleck B, Pazaitis N, Kohn M, Lederer M, Huttelmaier S: **Insulin-like growth factor 2 mRNA-binding proteins (IGF2BPs): post-transcriptional drivers of cancer progression?** *Cell. Mol. Life Sci.* 2012.

94. Fehringer G, Liu G, Pintilie M, Sykes J, Cheng D, Liu N, Chen Z, Seymour L, Der SD, Shepherd FA, Tsao MS, Hung RJ: **Association of the 15q25 and 5p15 lung cancer susceptibility regions with gene expression in lung tumor tissue**. *Cancer Epidemiol. Biomarkers Prev.* 2012, **21**(7):1097–1104.

95. Haro KJ, Sheth A, Scheinberg DA: **Dysregulation of IRP1-mediated iron metabolism causes gamma ray-specific radioresistance in leukemia cells**. *PLoS ONE* 2012, **7**(11):e48841.

96. Fu X, Xue C, Huang Y, Xie Y, Li Y: **The activity and expression of microRNAs in prostate cancers**. *Mol Biosyst* 2010, **6**(12):2561–2572.

97. Girault A, Haelters JP, Potier-Cartereau M, Chantome A, Pinault M, Marionneau-Lambot S, Oullier T, Simon G, Couthon-Gourves H, Jaffres PA, Corbel B, Bougnoux P, Joulin V, Vandier C: **New alkyl-lipid blockers of SK3 channels reduce cancer cell migration and occurrence of metastasis**. *Curr Cancer Drug Targets* 2011, **11**(9):1111–1125.

98. Deng XL, Lau CP, Lai K, Cheung KF, Lau GK, Li GR: **Cell cycle-dependent expression of potassium channels and cell proliferation in rat mesenchymal stem cells from bone marrow**. *Cell Prolif.* 2007, **40**(5):656–670.

99. Chen J, Jaeger K, Den Z, Koch PJ, Sundberg JP, Roop DR: **Mice expressing a mutant Krt75 (K6hf) allele develop hair and nail defects resembling pachyonychia congenita**. *J. Invest. Dermatol.* 2008, **128**(2):270–279.

100. Puente XS, Pinyol M, Quesada V, Conde L, Ordonez GR, Villamor N, Escaramis G, Jares P, Bea S, Gonzalez-Diaz M, Bassaganyas L, Baumann T, Juan M, Lopez-Guerra M, Colomer D, Tubio JM, Lopez C, Navarro A, Tornador C, Aymerich M, Rozman M, Hernandez JM, Puente DA, Freije JM, Velasco G, Gutierrez-Fernandez A, Costa D, Carrio A, Guijarro S, Enjuanes A, Hernandez L, Yague J, Nicolas P, Romeo-Casabona CM, Himmelbauer H, Castillo E, Dohm JC, de Sanjose S, Piris MA, de Alava E, San Miguel J, Royo R, Gelpi JL, Torrents D, Orozco M, Pisano DG, Valencia A, Guigo R, Bayes M, Heath S, Gut M, Klatt P, Marshall J, Raine K, Stebbings LA, Futreal PA, Stratton MR, Campbell PJ, Gut I, Lopez-Guillermo A, Estivill X, Montserrat E, Lopez-Otin C, Campo E: **Whole-genome sequencing identifies recurrent mutations in chronic lymphocytic leukaemia**. *Nature* 2011, **475**(7354):101–105.

101. Trifonov V, Pasqualucci L, Dalla Favera R, Rabadan R: **MutComFocal: an integrative approach to identifying recurrent and focal genomic alterations in tumor samples**. *BMC Syst Biol* 2013, **7**:25.

102. Bu X, Avraham HK, Li X, Lim B, Jiang S, Fu Y, Pestell RG, Avraham S: **Mayven induces c-Jun expression and cyclin D1 activation in breast cancer cells**. *Oncogene* 2005, **24**(14):2398–2409.

103. Colas E, Muinelo-Romay L, Alonso-Alconada L, Llaurado M, Monge M, Barbazan J, Gonzalez M, Schoumacher M, Pedrola N, Ertekin T, Devis L, Ruiz A, Castellvi J, Doll A, Gil-Moreno A, Vazquez-Levin M, Lapyckyj L, Lopez-Lopez R, Robine S, Friederich E, Castro M, Reventos J, Vignjevic D, Abal M: **ETV5 cooperates with LPP as a sensor of extracellular signals and promotes EMT in endometrial carcinomas**. *Oncogene* 2012, **31**(45):4778–4788.

104. Ngan E, Northey JJ, Brown CM, Ursini-Siegel J, Siegel PM: **A complex containing LPP and $\alpha$-actinin mediates TGF$\beta$-induced migration and invasion of ErbB2-expressing breast cancer cells**. *J. Cell. Sci.* 2013, **126**(Pt 9):1981–1991.

105. Stokes PH, Liew CW, Kwan AH, Foo P, Barker HE, Djamirze A, O'Reilly V, Visvader JE, Mackay JP, Matthews JM: **Structural basis of the interaction of the breast cancer oncogene LMO4 with the tumour suppressor CtIP/RBBP8**. *J. Mol. Biol.* 2013, **425**(7):1101–1110.

106. Vicente-Duenas C, Romero-Camarero I, Gonzalez-Herrero I, Alonso-Escudero E, Abollo-Jimenez F, Jiang X, Gutierrez NC, Orfao A, Marin N, Villar LM, Criado MC, Pintado B, Flores T, Alonso-Lopez D, De Las Rivas J, Jimenez R, Criado FJ, Cenador MB, Lossos IS, Cobaleda C, Sanchez-Garcia I: **A novel molecular mechanism involved in multiple myeloma development revealed by targeting MafB to haematopoietic progenitors**. *EMBO J.* 2012, **31**(18):3704–3717.

107. Lu J, Hamze Z, Bonnavion R, Herath N, Pouponnot C, Assade F, Fontaniere S, Bertolino P, Cordier-Bussat M, Zhang CX: **Reexpression of oncoprotein MafB in proliferative $\beta$-cells and Men1 insulinomas in mouse**. *Oncogene* 2012, **31**(31):3647–3654.

108. Kang JU, Koo SH, Kwon KC, Park JW, Kim JM: **Identification of novel candidate target genes, including EPHB3, MASP1 and SST at 3q26.2-q29 in squamous cell carcinoma of the lung**. *BMC Cancer* 2009, **9**:237.

109. Son JW, Jeong KJ, Jean WS, Park SY, Jheon S, Cho HM, Park CG, Lee HY, Kang J: **Genome-wide combination profiling of DNA copy number and methylation for deciphering biomarkers in non-small cell lung cancer patients**. *Cancer Lett.* 2011, **311**:29–37.

110. Sebban S, Farago M, Gashai D, Ilan L, Pikarsky E, Ben-Porath I, Katzav S: **Vav1 fine tunes p53 control of apoptosis versus proliferation in breast cancer**. *PLoS ONE* 2013, **8**:e54321.

111. Musa A, Lehrach H, Russo VA: **Distinct expression patterns of two zebrafish homologues of the human APP gene during embryonic development**. *Dev. Genes Evol.* 2001, **211**(11):563–567.

112. Fan X, Liu Y, Jiang J, Ma Z, Wu H, Liu T, Liu M, Li X, Tang H: **miR-20a promotes proliferation and invasion by targeting APP in human ovarian cancer cells**. *Acta Biochim. Biophys. Sin. (Shanghai)* 2010, **42**(5):318–324.

113. Tao S, Wang Z, Feng J, Hsu FC, Jin G, Kim ST, Zhang Z, Gronberg H, Zheng LS, Isaacs WB, Xu J, Sun J: **A genome-wide search for loci interacting with known prostate cancer risk-associated genetic variants**. *Carcinogenesis* 2012, **33**(3):598–603.

114. Takita J, Chen Y, Okubo J, Sanada M, Adachi M, Ohki K, Nishimura R, Hanada R, Igarashi T, Hayashi Y, Ogawa S: **Aberrations of NEGR1 on 1p31 and MYEOV on 11q13 in neuroblastoma**. *Cancer Sci.* 2011, **102**(9):1645–1650.

115. Vasseur S, Malicet C, Calvo EL, Dagorn JC, Iovanna JL: **Gene expression profiling of tumours derived from rasV12/E1A-transformed mouse embryonic fibroblasts to identify genes required for tumour development**. *Mol. Cancer* 2005, **4**:4.

116. Zhou W, Zhang F, Chen X, Shen Y, Lupski JR, Jin L: **Increased genome instability in human DNA segments with self-chains: homology-induced structural variations via replicative mechanisms**. *Hum. Mol. Genet.* 2013, **22**(13):2642–2651.

117. Ding D, Lou X, Hua D, Yu W, Li L, Wang J, Gao F, Zhao N, Ren G, Li L, Lin B: **Recurrent targeted genes of hepatitis B virus in the liver cancer genomes identified by a next-generation sequencing-based approach**. *PLoS Genet.* 2012, **8**(12):e1003065.

118. Chen P, Li K, Liang Y, Li L, Zhu X: **High NUAK1 expression correlates with poor prognosis and involved in NSCLC cells migration and invasion**. *Exp. Lung Res.* 2013, **39**:9–17.

119. Lu S, Niu N, Guo H, Tang J, Guo W, Liu Z, Shi L, Sun T, Zhou F, Li H, Zhang J, Zhang B: **ARK5 promotes glioma cell invasion, and its elevated expression is correlated with poor clinical outcome**. *Eur. J. Cancer* 2013, **49**(3):752–763.

120. Kato T, Satoh S, Okabe H, Kitahara O, Ono K, Kihara C, Tanaka T, Tsunoda T, Yamaoka Y, Nakamura Y, Furukawa Y: **Isolation of a novel human gene, MARKL1, homologous to MARK3 and its involvement in hepatocellular carcinogenesis**. *Neoplasia* 2001, **3**:4–9.

121. Surdez D, Benetkiewicz M, Perrin V, Han ZY, Pierron G, Ballet S, Lamoureux F, Redini F, Decouvelaere AV, Daudigeos-Dubus E, Geoerger B, de Pinieux G, Delattre O, Tirode F: **Targeting the EWSR1-FLI1 oncogene-induced protein kinase PKC-$\beta$ abolishes ewing sarcoma growth**. *Cancer Res.* 2012, **72**(17):4494–4503.

122. Faryna M, Konermann C, Aulmann S, Bermejo JL, Brugger M, Diederichs S, Rom J, Weichenhan D, Claus R, Rehli M, Schirmacher P, Sinn HP, Plass C, Gerhauser C: **Genome-wide methylation screen in low-grade breast cancer identifies novel epigenetically altered genes as potential biomarkers for tumor diagnosis**. *FASEB J.* 2012, **26**(12):4937–4950.

123. Hong SM, Omura N, Vincent A, Li A, Knight S, Yu J, Hruban RH, Goggins M: **Genome-wide CpG island profiling of intraductal papillary mucinous neoplasms of the pancreas**. *Clin. Cancer Res.* 2012, **18**(3):700–712.

124. Kim H, Kim K, Yu SJ, Jang ES, Yu J, Cho G, Yoon JH, Kim Y: **Development of biomarkers for screening hepatocellular carcinoma using global data mining and multiple reaction monitoring**. *PLoS ONE* 2013, **8**(5):e63468.

125. Hatakeyama S: **TRIM proteins and cancer**. *Nat. Rev. Cancer* 2011, **11**(11):792–804.

126. Youker RT, Shinde U, Day R, Thomas G: **At the crossroads of homoeostasis and disease: roles of the PACS proteins in membrane traffic and apoptosis**. *Biochem. J.* 2009, **421**:1–15.

127. Kamarajugadda S, Stemboroski L, Cai Q, Simpson NE, Nayak S, Tan M, Lu J: **Glucose oxidation modulates anoikis and tumor metastasis**. *Mol. Cell. Biol.* 2012, **32**(10):1893–1907.

128. Brisbin AG, Asmann YW, Song H, Tsai YY, Aakre JA, Yang P, Jenkins RB, Pharoah P, Schumacher F, Conti DV, Duggan DJ, Jenkins M, Hopper J, Gallinger S, Newcomb P, Casey G, Sellers TA, Fridley BL: **Meta-analysis of 8q24 for seven cancers reveals a locus between NOV and ENPP2 associated with cancer development**. *BMC Med. Genet.* 2011, **12**:156.

129. Li L, Yu C, Gao H, Li Y: **Argonaute proteins: potential biomarkers for human colon cancer**. *BMC Cancer* 2010, **10**:38.

130. Papagerakis S, Shabana AH, Depondt J, Gehanno P, Forest N: **Immunohistochemical localization of plakophilins (PKP1, PKP2, PKP3, and p0071) in primary oropharyngeal tumors: correlation with clinical parameters**. *Hum. Pathol.* 2003, **34**(6):565–572.

131. Walter B, Berger I, Hofmann I: **The proteins ARVCF and p0071 in renal cell carcinomas and their potential use in the diagnosis of renal tumours**. *Histopathology* 2009, **55**(6):761–764.

132. Prochazkova J, Kabatkova M, Smerdova L, Pachernik J, Sykorova D, Kohoutek J, Simeckova P, Hruba E, Kozubik A, Machala M, Vondracek J: **Aryl Hydrocarbon Receptor Negatively Regulates Expression of the Plakoglobin Gene (Jup)**. *Toxicol. Sci.* 2013.

133. Gomez-Morales M, Camara-Pulido M, Miranda-Leon MT, Sanchez-Palencia A, Boyero L, Gomez-Capilla JA, Farez-Vidal ME: **Differential immunohistochemical localization of desmosomal plaque-related proteins in non-small-cell lung cancer**. *Histopathology* 2013, **63**:103–113.

134. Zhang X, Wu Q, Gan L, Yu GZ, Wang R, Wang ZS, Wang JJ, Wang X: **Reduced group IVA phospholipase A2 expression is associated with unfavorable outcome for patients with gastric cancer**. *Med. Oncol.* 2013, **30**:454.

135. Yu Y, Zhang M, Pan Y, Jin M, Jiang X, Zhang S, Wu Y, Ni Q, Li Q, Chen K: **PLA2G4A mutants modified protective effect of tea consumption against colorectal cancer**. *Int J Colorectal Dis* 2012, **27**(8):1005–1013.

136. Gonzalez-Arriagada WA, Santos-Silva AR, Ito FA, Vargas PA, Speight PM, Bingle L, Lopes MA: **Expression pattern of PLUNC proteins as an auxiliary tool**

**for the diagnosis of high-grade mucoepidermoid carcinoma of the salivary gland**. *J. Oral Pathol. Med.* 2012, **41**(8):589–597.

137. Dentener MA, Francot GJ, Hiemstra PS, Tool AT, Verhoeven AJ, Vandenabeele P, Buurman WA: **Bactericidal/permeability-increasing protein release in whole blood ex vivo: strong induction by lipopolysaccharide and tumor necrosis factor-alpha**. *J. Infect. Dis.* 1997, **175**:108–117.

138. Dancau AM, Wuth L, Waschow M, Holst F, Krohn A, Choschzick M, Terracciano L, Politis S, Kurtz S, Lebeau A, Friedrichs K, Wencke K, Monni O, Simon R: **PPFIA1 and CCND1 are frequently coamplified in breast cancer**. *Genes Chromosomes Cancer* 2010, **49**:1–8.

139. Fujinami K, Uemura H, Ishiguro H, Kubota Y: **Liprin-alpha2 gene, protein tyrosine phosphatase LAR interacting protein related gene, is downregulated by androgens in the human prostate cancer cell line LNCaP**. *Int. J. Mol. Med.* 2002, **10**(2):173–176.

140. Scott A, Wang Z: **Tumour suppressor function of protein tyrosine phosphatase receptor-T**. *Biosci. Rep.* 2011, **31**(5):303–307.

141. Xu Y, Fisher GJ: **Receptor type protein tyrosine phosphatases (RPTPs) - roles in signal transduction and human disease**. *J Cell Commun Signal* 2012, **6**(3):125–138.

142. Zhou L, Picard D, Ra YS, Li M, Northcott PA, Hu Y, Stearns D, Hawkins C, Taylor MD, Rutka J, Der SD, Huang A: **Silencing of thrombospondin-1 is critical for myc-induced metastatic phenotypes in medulloblastoma**. *Cancer Res.* 2010, **70**(20):8199–8210.

143. Yusenko MV, Nagy A, Kovacs G: **Molecular analysis of germline t(3;6) and t(3;12) associated with conventional renal cell carcinomas indicates their rate-limiting role and supports the three-hit model of carcinogenesis**. *Cancer Genet. Cytogenet.* 2010, **201**:15–23.

144. Rogers LM, Riordan JD, Swick BL, Meyerholz DK, Dupuy AJ: **Ectopic expression of zmiz1 induces cutaneous squamous cell malignancies in a mouse model of cancer**. *J. Invest. Dermatol.* 2013, **133**(7):1863–1869.

145. Li X, Zhu C, Tu WH, Yang N, Qin H, Sun Z: **ZMIZ1 preferably enhances the transcriptional activity of androgen receptor with short polyglutamine tract**. *PLoS ONE* 2011, **6**(9):e25040.

146. Antoniou AC, Kuchenbaecker KB, Soucy P, Beesley J, Chen X, McGuffog L, Lee A, Barrowdale D, Healey S, Sinilnikova OM, Caligo MA, Loman N, Harbst K, Lindblom A, Arver B, Rosenquist R, Karlsson P, Nathanson K, Domchek S, Rebbeck T, Jakubowska A, Lubinski J, Jaworska K, Durda K, Z?owowcka-Per?owska E, Osorio A, Duran M, Andres R, Benitez J, Hamann U, Hogervorst FB, van Os TA, Verhoef S, Meijers-Heijboer HE, Wijnen J, Gomez Garcia EB, Ligtenberg MJ, Kriege M, Collee JM, Ausems MG, Oosterwijk JC, Peock S, Frost D, Ellis SD, Platte R, Fineberg E, Evans DG, Lalloo F, Jacobs C, Eeles R, Adlard J, Davidson R, Cole T, Cook J, Paterson J, Douglas F, Brewer C, Hodgson S, Morrison PJ, Walker L, Rogers MT, Donaldson A, Dorkins H, Godwin AK, Bove B, Stoppa-Lyonnet D, Houdayer C, Buecher B, de Pauw A, Mazoyer S, Calender A, Leone M, Bressac-de Paillerets B, Caron O, Sobol H, Frenay M, Prieur F, Ferrer SU, Mortemousque I, Buys S, Daly M, Miron A, Terry MU, Hopper JL, John EM, Southey M, Goldgar D, Singer CF, Fink-Retter A, Tea MK, Kaulich DU, Hansen TV, Nielsen FC, Barkardottir RB, Gaudet M, Kirchhoff T, Joseph V, Dutra-Clarke A, Offit K, Piedmonte M, Kirk J, Cohn D, Hurteau J, Byron J, Fiorica J, Toland AE, Montagna M, Oliani C, Imyanitov E, Isaacs C, Tihomirova L, Blanco I, Lazaro C, Teule A, Valle JD, Gayther SA, Odunsi K, Gross J, Karlan BY, Olah E, Teo SH, Ganz PA, Beattie MS, Dorfling CM, van Rensburg EU, Diez O, Kwong A, Schmutzler RK, Wappenschmidt B, Engel C, Meindl A, Ditsch N, Arnold N, Heidemann S, Niederacher D, Preisler-Adams S, Gadzicki D, Varon-Mateeva R, Deissler H, Gehrig A, Sutter C, Kast K, Fiebig B, Schafer D, Caldes T, de la Hoya M, Nevanlinna H, Muranen TA, Lesperance B, Spurdle AB, Neuhausen SL, Ding YC, Wang X, Fredericksen Z, Pankratz VS, Lindor NM, Peterlongo P, Manoukian S, Peissel B, Zaffaroni D, Bonanni B, Bernard L, Dolcetti R, Papi L, Ottini L, Radice P, Greene MH, Loud JT, Andrulis IL, Ozcelik H, Mulligan AU, Glendon G, Thomassen M, Gerdes AM, Jensen UB, Skytte AB, Kruse TA, Chenevix-Trench G, Couch FJ, Simard J, Easton DF: **Common variants at 12p11, 12q24, 9p21, 9q31.2 and in ZNF365 are associated with breast cancer risk for BRCA1 and/or BRCA2 mutation carriers**. *Breast Cancer Res.* 2012, **14**:R33.

147. Lee J, Beliakoff J, Sun Z: **The novel PIAS-like protein hZimp10 is a transcriptional co-activator of the p53 tumor suppressor**. *Nucleic Acids Res.* 2007, **35**(13):4523–4534.

148. Beliakoff J, Sun Z: **Zimp7 and Zimp10, two novel PIAS-like proteins, function as androgen receptor coregulators**. *Nucl Recept Signal* 2006, **4**:e017.

149. Zhang X, He Y, Lee KH, Dubois W, Li Z, Wu X, Kovalchuk A, Zhang W, Huang J: **Rap2b, a novel p53 target, regulates p53-mediated pro-survival function**. *Cell Cycle* 2013, **12**(8):1279–1291.

150. Liu Y, Sun W, Zhang K, Zheng H, Ma Y, Lin D, Zhang X, Feng L, Lei W, Zhang Z, Guo S, Han N, Tong W, Feng X, Gao Y, Cheng S: **Identification of genes differentially expressed in human primary lung squamous cell carcinoma**. *Lung Cancer* 2007, **56**(3):307–317.

151. Hu YL, Zhong D, Pang F, Ning QY, Zhang YY, Li G, Wu JZ, Mo ZN: **HNF1b is involved in prostate cancer risk via modulating androgenic hormone effects and coordination with other genes**. *Genet. Mol. Res.* 2013, **12**(2):1327–1335.

152. Chen W, Yuan L, Cai Y, Chen X, Chi Y, Wei P, Zhou X, Shi D: **Identification of chromosomal copy number variations and novel candidate loci in hereditary nonpolyposis colorectal cancer with mismatch repair proficiency**. *Genomics* 2013, **102**:27–34.

153. Tan EH, Ramlau R, Pluzanska A, Kuo HP, Reck M, Milanowski J, Au JS, Felip E, Yang PC, Damyanov D, Orlov S, Akimov M, Delmar P, Essioux L, Hillenbach C, Klughammer B, McLoughlin P, Baselga J: **A multicentre phase II gene expression profiling study of putative relationships between tumour biomarkers and clinical response with erlotinib in non-small-cell lung cancer**. *Ann. Oncol.* 2010, **21**(2):217–222.

154. Hong J, Doebele RC, Lingen MW, Quilliam LA, Tang WJ, Rosner MR: **Anthrax edema toxin inhibits endothelial cell chemotaxis via Epac and Rap1**. *J. Biol. Chem.* 2007, **282**(27):19781–19787.

155. Xia H, Li M, Chen L, Leng W, Yuan D, Pang X, Chen L, Li R, Tang Q, Bi F: **Suppression of RND3 activity by AES downregulation promotes cancer cell proliferation and invasion**. *Int. J. Mol. Med.* 2013, **31**(5):1081–1086.

156. Riento K, Villalonga P, Garg R, Ridley A: **Function and regulation of RhoE**. *Biochem. Soc. Trans.* 2005, **33**(Pt 4):649–651.

157. Chardin P: **Function and regulation of Rnd proteins**. *Nat. Rev. Mol. Cell Biol.* 2006, **7**:54–62.

158. Goh LL, Manser E: **The GTPase-deficient Rnd proteins are stabilized by their effectors**. *J. Biol. Chem.* 2012, **287**(37):31311–31320.

159. Belgiovine C, Frapolli R, Bonezzi K, Chiodi I, Favero F, Mello-Grand M, Dei Tos AP, Giulotto E, Taraboletti G, D'Incalci M, Mondello C: **Reduced expression of the ROCK inhibitor Rnd3 is associated with increased invasiveness and metastatic potential in mesenchymal tumor cells**. *PLoS ONE* 2010, **5**(11):e14154.

160. Grise F, Sena S, Bidaud-Meynard A, Baud J, Hiriart JB, Makki K, Dugot-Senant N, Staedel C, Bioulac-Sage P, Zucman-Rossi J, Rosenbaum J, Moreau V: **Rnd3/RhoE Is down-regulated in hepatocellular carcinoma and controls cellular invasion**. *Hepatology* 2012, **55**(6):1766–1775.

161. Liu Y, Zhu X, Zhu J, Liao S, Tang Q, Liu K, Guan X, Zhang J, Feng Z: **Identification of differential expression of genes in hepatocellular carcinoma by suppression subtractive hybridization combined cDNA microarray**. *Oncol. Rep.* 2007, **18**(4):943–951.

162. Huang XP, Zhao CX, Li QJ, Cai Y, Liu FX, Hu H, Xu X, Han YL, Wu M, Zhan QM, Wang MR: **Alteration of RPL14 in squamous cell carcinomas and preneoplastic lesions of the esophagus**. *Gene* 2006, **366**:161–168.

163. Lu S, Lee J, Revelo M, Wang X, Lu S, Dong Z: **Smad3 is overexpressed in advanced human prostate cancer and necessary for progressive growth of prostate cancer cells in nude mice**. *Clin. Cancer Res.* 2007, **13**(19):5692–5702.

164. Yang J, Wahdan-Alaswad R, Danielpour D: **Critical role of Smad2 in tumor suppression and transforming growth factor-beta-induced apoptosis of prostate epithelial cells**. *Cancer Res.* 2009, **69**(6):2185–2190.

165. McPherson A, Wu C, Wyatt AW, Shah S, Collins C, Sahinalp SC: **nFuse: discovery of complex genomic rearrangements in cancer using high-throughput sequencing**. *Genome Res.* 2012, **22**(11):2250–2261.

166. Zhao Q, Caballero OL, Levy S, Stevenson BJ, Iseli C, de Souza SJ, Galante PA, Busam D, Leversha MA, Chadalavada K, Rogers YH, Venter JC, Simpson AJ, Strausberg RL: **Transcriptome-guided characterization of genomic rearrangements in a breast cancer cell line**. *Proc. Natl. Acad. Sci. U.S.A.* 2009, **106**(6):1886–1891.

167. Riggi N, Suva ML, Suva D, Cironi L, Provero P, Tercier S, Joseph JM, Stehle JC, Baumer K, Kindler V, Stamenkovic I: **EWS-FLI-1 expression triggers a Ewing's sarcoma initiation program in primary human mesenchymal stem cells**. *Cancer Res.* 2008, **68**(7):2176–2185.

168. Miyato H, Tsuno NH, Kitayama J: **Semaphorin 3C is involved in the progression of gastric cancer**. *Cancer Sci.* 2012, **103**(11):1961–1966.

169. Mazurek AM, Olbryt M: **The influence of neuropilin-1 silencing on semaphorin 3A and 3C activity in B16(F10) murine melanoma cells**. *Neoplasma* 2012, **59**:43–51.

170. Ch'ng ES, Kumanogoh A: **Roles of Sema4D and Plexin-B1 in tumor progression**. *Mol. Cancer* 2010, **9**:251.

171. Sabag AD, Bode J, Fink D, Kigel B, Kugler W, Neufeld G: **Semaphorin-3D and semaphorin-3E inhibit the development of tumors from glioblastoma cells implanted in the cortex of the brain**. *PLoS ONE* 2012, **7**(8):e42912.

172. Karayan-Tapon L, Wager M, Guilhot J, Levillain P, Marquant C, Clarhaut J, Potiron V, Roche J: **Semaphorin, neuropilin and VEGF expression in glial tumours: SEMA3G, a prognostic marker?** *Br. J. Cancer* 2008, **99**(7):1153–1160.

173. Toyofuku T, Yabuki M, Kamei J, Kamei M, Makino N, Kumanogoh A, Hori M: **Semaphorin-4A, an activator for T-cell-mediated immunity, suppresses angiogenesis via Plexin-D1**. *EMBO J.* 2007, **26**(5):1373–1384.

174. Shen HJ, Zhu HY, Yang C, Ji F: **SENP2 regulates hepatocellular carcinoma cell growth by modulating the stability of $\beta$-catenin**. *Asian Pac. J. Cancer Prev.* 2012, **13**(8):3583–3587.

175. Kwon YE, Bae SJ, Kim M, Seol JH: **SUMOylation negatively regulates the stability of CHFR tumor suppressor**. *Biochem. Biophys. Res. Commun.* 2013, **430**:213–217.

176. Ouyang YQ, zur Hausen A, Orlowska-Volk M, Jager M, Bettendorf H, Hirschfeld M, Tong XW, Stickeler E: **Expression levels of hnRNP G and hTra2-beta1 correlate with opposite outcomes in endometrial cancer biology**. *Int. J. Cancer* 2011, **128**(9):2010–2019.

177. Gabriel B, Zur Hausen A, Bouda J, Boudova L, Koprivova M, Hirschfeld M, Jager M, Stickeler E: **Significance of nuclear hTra2-beta1 expression in cervical cancer**. *Acta Obstet Gynecol Scand* 2009, **88**(2):216–221.

178. Silva JM, Boczek NJ, Berres MW, Ma X, Smith DI: **LSINCT5 is over expressed in breast and ovarian cancer and affects cellular proliferation**. *RNA Biol* 2011, **8**(3):496–505.

179. Jiang DK, Sun J, Cao G, Liu Y, Lin D, Gao YZ, Ren WH, Long XD, Zhang H, Ma XP, Wang Z, Jiang W, Chen TY, Gao Y, Sun LD, Long JR, Huang HX, Wang D, Yu H, Zhang P, Tang LS, Peng B, Cai H, Liu TT, Zhou P, Liu F, Lin X, Tao S, Wan B, Sai-Yin HX, Qin LX, Yin J, Liu L, Wu C, Pei Y, Zhou YF, Zhai Y, Lu PX, Tan A, Zuo XB, Fan J, Chang J, Gu X, Wang NJ, Li Y, Liu YK, Zhai K, Zhang H, Hu Z, Liu J, Yi Q, Xiang Y, Shi R, Ding Q, Zheng W, Shu XO, Mo Z, Shugart YY, Zhang XJ, Zhou G, Shen H, Zheng SL, Xu J, Yu L: **Genetic variants in STAT4 and HLA-DQ genes confer risk of hepatitis B virus-related hepatocellular carcinoma**. *Nat. Genet.* 2013, **45**:72–75.

180. Turkson J: **STAT proteins as novel targets for cancer drug discovery**. *Expert Opin. Ther. Targets* 2004, **8**(5):409–422.

181. Kovacic B, Stoiber D, Moriggl R, Weisz E, Ott RG, Kreibich R, Levy DE, Beug H, Freissmuth M, Sexl V: **STAT1 acts as a tumor promoter for leukemia development**. *Cancer Cell* 2006, **10**:77–87.

182. Lee HS, Lee DC, Park MH, Yang SJ, Lee JJ, Kim DM, Jang Y, Lee JH, Choi JY, Kang YK, Kim DI, Park KC, Kim SY, Yoo HS, Choi EJ, Yeom YI: **STMN2 is a novel target of beta-catenin/TCF-mediated transcription in human hepatoma cells**. *Biochem. Biophys. Res. Commun.* 2006, **345**(3):1059–1067.

183. Rogers DA, Schor NF: **Kidins220/ARMS depletion is associated with the neural-to Schwann-like transition in a human neuroblastoma cell line model**. *Exp. Cell Res.* 2013, **319**(5):660–669.

184. Sung PJ, Boulos N, Tilby MJ, Andrews WD, Newbold RF, Tweddle DA, Lunec J: **Identification and characterisation of STMN4 and ROBO2 gene involvement in neuroblastoma cell differentiation**. *Cancer Lett.* 2013, **328**:168–175.

185. Scales SJ, Hesser BA, Masuda ES, Scheller RH: **Amisyn, a novel syntaxin-binding protein that may regulate SNARE complex assembly**. *J. Biol. Chem.* 2002, **277**(31):28271–28279.

186. Poste G, Nicolson GL: **Arrest and metastasis of blood-borne tumor cells are modified by fusion of plasma membrane vesicles from highly metastatic cells**. *Proc. Natl. Acad. Sci. U.S.A.* 1980, **77**:399–403.

187. Zaravinos A, Lambrou GI, Boulalas I, Delakas D, Spandidos DA: **Identification of common differentially expressed genes in urinary bladder cancer**. *PLoS ONE* 2011, **6**(4):e18135.

188. Bierie B, Moses HL: **Tumour microenvironment: TGFbeta: the molecular Jekyll and Hyde of cancer**. *Nat. Rev. Cancer* 2006, **6**(7):506–520.

189. Hou YL, Chen H, Dong ZH, Xue CJ, Wu YF, Luo HX, Wu YC, Li CH: **Clinical significance of serum transforming growth factor-$\beta$1 in lung cancer**. *Cancer Epidemiol* 2013.

190. Duvvuri U, Shiwarski DJ, Xiao D, Bertrand C, Huang X, Edinger RS, Rock JR, Harfe BD, Henson BJ, Kunzelmann K, Schreiber R, Seethala RS, Egloff AM, Chen X, Lui VW, Grandis JR, Gollin SM: **TMEM16A induces MAPK and contributes directly to tumorigenesis and cancer progression**. *Cancer Res.* 2012, **72**(13):3270–3281.

191. Sugahara K, Michikawa Y, Ishikawa K, Shoji Y, Iwakawa M, Shibahara T, Imai T: **Combination effects of distinct cores in 11q13 amplification region on cervical lymph node metastasis of oral squamous cell carcinoma**. *Int. J. Oncol.* 2011, **39**(4):761–769.

192. Cai CJ, Ye SJ, Zhu W, Sun LY, Wan HS, Feng ZH, Ma L, Li L: **[Study on invasion and metastasis-associated genes of lung cancer related with NM23-H1 gene]**. *Sichuan Da Xue Xue Bao Yi Xue Ban* 2010, **41**(6):941–945.

193. Shi Y, Hu Z, Wu C, Dai J, Li H, Dong J, Wang M, Miao X, Zhou Y, Lu F, Zhang H, Hu L, Jiang Y, Li Z, Chu M, Ma H, Chen J, Jin G, Tan W, Wu T, Zhang Z, Lin D, Shen H: **A genome-wide association study identifies new susceptibility loci for non-cardia gastric cancer at 3q13.31 and 5p13.1**. *Nat. Genet.* 2011, **43**(12):1215–1218.

194. Virgone C, Cecchetto G, Ferrari A, Bisogno G, Donofrio V, Boldrini R, Collini P, Dall'Igna P, Alaggio R: **GATA-4 and FOG-2 expression in pediatric ovarian sex cord-stromal tumors replicates embryonal gonadal phenotype: results from the TREP project**. *PLoS ONE* 2012, **7**(9):e45914.

195. Karlsson J, Holmquist Mengelbier L, Elfving P, Gisselsson Nord D: **High-resolution genomic profiling of an adult Wilms' tumor: evidence for a pathogenesis distinct from corresponding pediatric tumors**. *Virchows Arch.* 2011, **459**(5):547–553.

196. Tommerup N, Vissing H: **Isolation and fine mapping of 16 novel human zinc finger-encoding cDNAs identify putative candidate genes for developmental and malignant disorders**. *Genomics* 1995, **27**(2):259–264.

197. Onizuka S, Kawakami S, Taniguchi K, Fujioka H, Miyashita K: **Pancreatic carcinogenesis: apoptosis and angiogenesis**. *Pancreas* 2004, **28**(3):317–319.

198. Feichtinger J, Aldeailej I, Anderson R, Almutairi M, Almatrafi A, Alsiwiehri N, Griffiths K, Stuart N, Wakeman JA, Larcombe L, McFarlane RJ: **Meta-analysis of clinical data using human meiotic genes identifies a novel cohort of highly restricted cancer-specific marker genes**. *Oncotarget* 2012, **3**(8):843–853.

199. Kim YA, Wuchty S, Przytycka TM: **Identifying causal genes and dysregulated pathways in complex diseases**. *PLoS Comput. Biol.* 2011, **7**(3):e1001095.

200. Chiang WF, Hung PS, Liu SY, Yuan TC, Chang KW, Chen YP, Liu YC, Lin SC: **Increase of ZASC1 gene copy number in recurrent oral carcinoma**. *Oral Dis* 2011, **17**:53–59.

201. Bruce JW, Hierl M, Young JA, Ahlquist P: **Cellular transcription factor ZASC1 regulates murine leukemia virus transcription**. *J. Virol.* 2010, **84**(15):7473–7483.

202. Wu WK, Lee CW, Cho CH, Chan FK, Yu J, Sung JJ: **RNA interference targeting raptor inhibits proliferation of gastric cancer cells**. *Exp. Cell Res.* 2011, **317**(10):1353–1358.

203. Yonezawa K, Tokunaga C, Oshiro N, Yoshino K: **Raptor, a binding partner of target of rapamycin**. *Biochem. Biophys. Res. Commun.* 2004, **313**(2):437–441.

204. Lupini L, Bassi C, Ferracin M, Bartonicek N, D'Abundo L, Zagatti B, Callegari E, Musa G, Moshiri F, Gramantieri L, Corrales FJ, Enright AJ, Sabbioni S, Negrini M: **miR-221 affects multiple cancer pathways by modulating the level of hundreds messenger RNAs**. *Front Genet* 2013, **4**:64.

205. Dai ZJ, Gao J, Kang HF, Ma YG, Ma XB, Lu WF, Lin S, Ma HB, Wang XJ, Wu WY: **Targeted inhibition of mammalian target of rapamycin (mTOR) enhances radiosensitivity in pancreatic carcinoma cells**. *Drug Des Devel Ther* 2013, **7**:149–159.
