# Supplementary figures and images for: Genes associated with genotype-specific DNA methylation in squamous cell carcinoma as candidate drug targets
Source: BMC Syst Biol. 2014 Jan 24;8(Suppl 1):S4. doi: 10.1186/1752-0509-8-S1-S4 (PMC4080267; doi:10.1186/1752-0509-8-S1-S4)

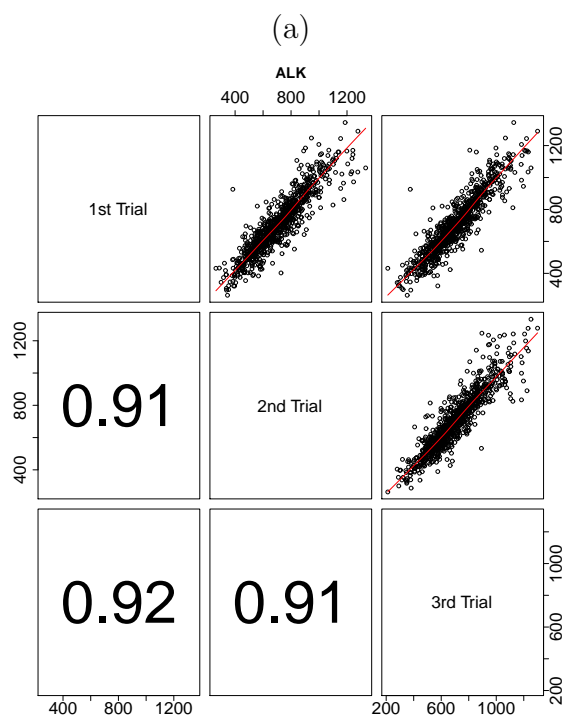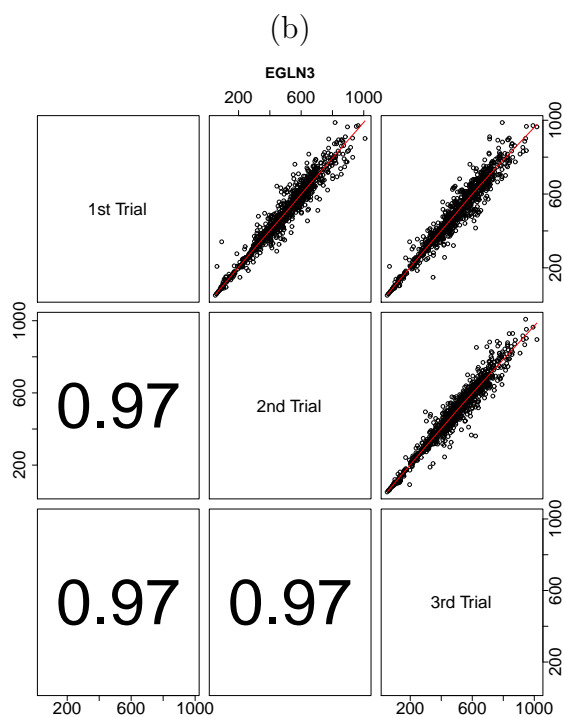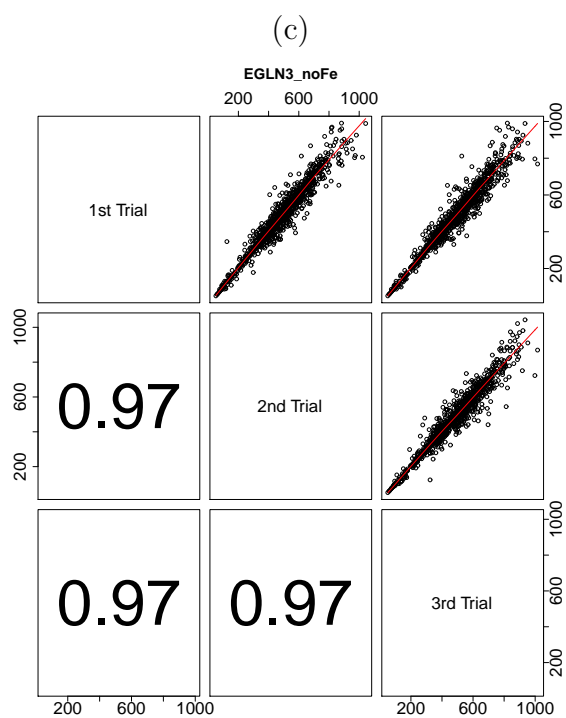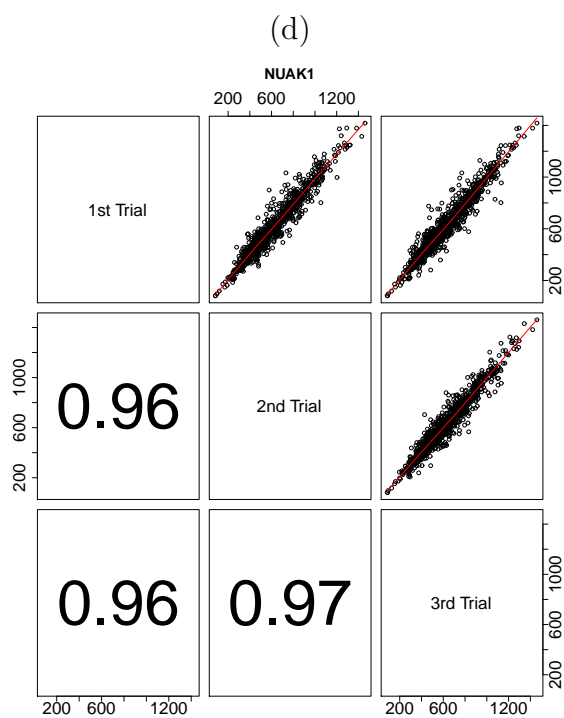

Supplement: Additional file 6 — Pearson correlation coefficients and scatter plots between independent trials for FPAScore computation. (a) ALK; (b) EGLN3 with Fe. (c) EGLN3 without Fe. (d) NUAK1. Scatter plots are shown for reference. Red diagonal lines indicate that FPAScores were identical between two trials. [file 1752-0509-8-S1-S4-S6.pdf]

(a)

**ALK**

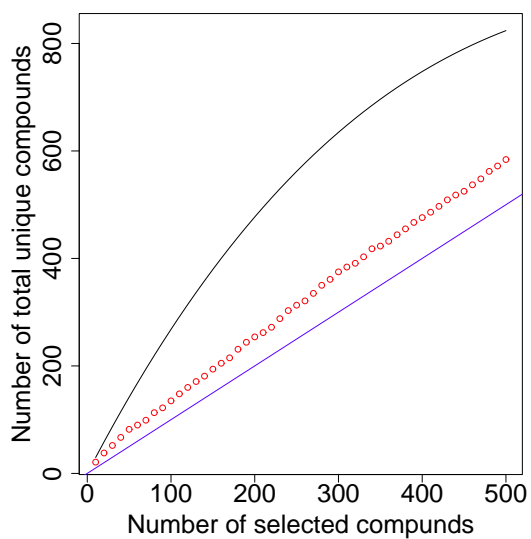

(b)

**EGLN3**

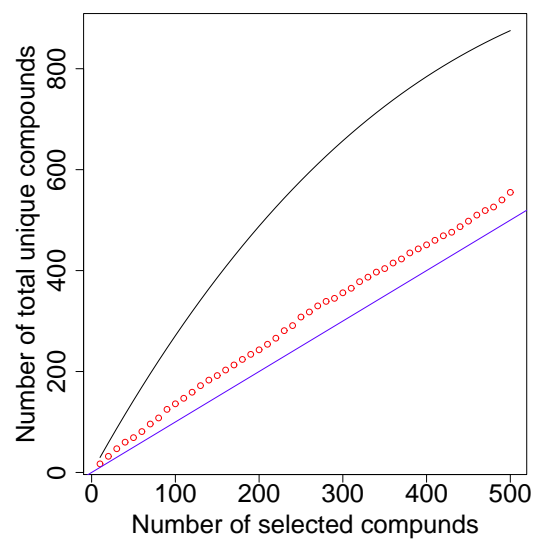

(c)

**EGLN3\_noFe**

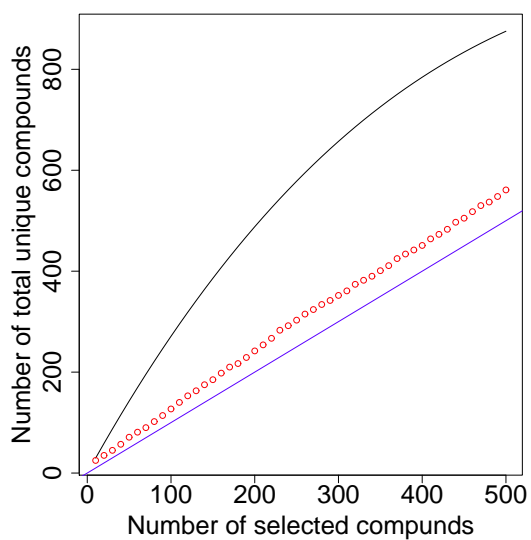

(d)

**NUAK1**

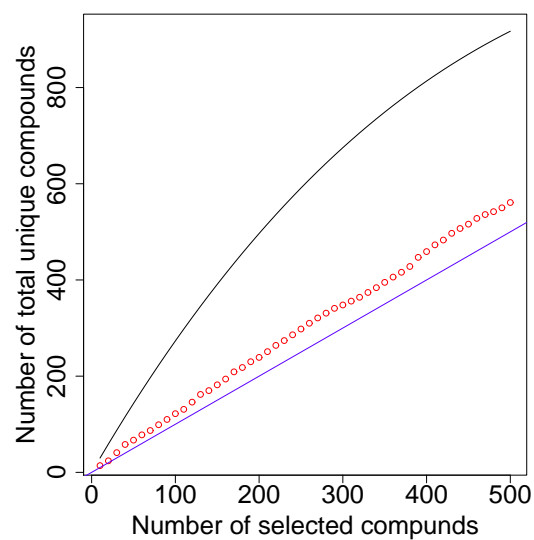

Supplement: Additional file 7 — Number of compounds selected in common in three trials as highly feasible drug candidate compounds. The number of common selections from among the top k ranked compounds in three trials (red circles). Black solid line indicates the expected number of compounds to be selected in common between three trials when the three trials are not correlated at all (n0(k), see Methods for details). Blue straight line indicates a complete match between the three trials. (a) ALK. (b) EGLN3 with Fe. (c) EGLN3 without Fe. (d) NUAK1. It is clear that the number of selections in common is much less than would be expected for random selections, n0(k), and is very close to a complete match (blue line). [file 1752-0509-8-S1-S4-S7.pdf]
