# Supplementary material for: Genes associated with genotype-specific DNA methylation in squamous cell carcinoma as candidate drug targets
Source: BMC Syst Biol. 2014 Jan 24;8(Suppl 1):S4. doi: 10.1186/1752-0509-8-S1-S4 (PMC4080267; doi:10.1186/1752-0509-8-S1-S4)
Supplement: Additional file 9 — Detailed discussion of the top 10 compounds listed in Table 7. Target protein information and protein inhibition information were taken from DrugBank [22,23] and ChEMBL [28,29]. [file 1752-0509-8-S1-S4-S9.pdf]

Supporting Information: Previous studies that reported the relationship between cancers/tumors and top most feasible drug canridate compounds selected for ALK, ELGN3 and NUAK1

Ryoichi Kinoshita[1] and Mitsuo Iwadate[2] and Hideaki Umeyama[2] and Y-h. Taguchi*[1]

[1]Department of Physics, Chuo University, 1-13-27 Kasuga, Bunkyo-ku, Tokyo 112-8551, Japan
[2]Department of Biological Science, Chuo University, 1-13-27 Kasuga, Bunkyo-ku, Tokyo 112-8551, Japan

Email: Mitsuo Iwadate - iwadate@bio.chuo-u.ac.jp; Hideaki Umeyama - umeyama@bio.chuo-u.ac.jp; Y-h. Taguchi - tag@granular.com;

*Corresponding author

Citations/reports that related drug candidate compounds to cancers

Each compound listed to target each gene was shown below in the descending-order of FPAScores averaged over three trials. Compound IDs were taken from DrugBank.

ALK

7-Hydroxystaurosporine (DB01933)

This compound was reported to target 3-phosphoinositide dependent protein kinase-1 (PDPK1 or PDK1) [1, 2]. PDK1 was reported to be cancer-related therapeutic target gene [3]. This compound was also included in National Cancer Institute Drug Dictonray (NCIDD) that says "A synthetic derivative of staurosporine with antineoplastic activity. 7-hydroxystaurosporine inhibits many phosphokinases, including the serine/threonine kinase AKT, calcium-dependent protein kinase C, and cyclin-dependent kinases. This agent arrests tumor cells in the G1/S of the cell cycle and prevents nucleotide excision repair by inhibiting the G2 checkpoint kinase chk1, resulting in apoptosis."

3-[(1R)-1-(2,6-dichloro-3-fluorophenyl)ethoxy]-5-(1-piperidin-4-yl-1H-pyrazol-4-yl)pyridin-2-amine (DB08700)

this compound was reported to target ALK, LCK, c-MET, TRKA, TRKB, TIE2 and ABL [4]. Hepatocyte growth factor receptor (c-MET) was also reported to be targeted by this compound in an addtional report [5]. c-MET was reported to be cancer-related therapeutic target gene [6]. LCK [4] that was reported to a tumor antigen [7]. TRKA and TRKB are receptors of Trk kinase whose inhibition was reported to be as new treatments for cancer and pain [8]. Targeting the TIE2 was reported to inhibit tumor growth and metastasis [9]. Activation of Abl Tyrosine Kinases was reported to promote invasion of aggressive breast cancer cells [10].

BIOTINOL-5-AMP (DB04651)

There were no reports that suggest tight relationship with cancers.

4-[4-(1-Amino-1-Methylethyl)Phenyl]-5-Chloro-N-[4-(2-Morpholin-4-Ylethyl)Phenyl] Pyrimidin-2-Amine (DB02491)

This compound was reported to target FGFR-2 [1,2] that was reported to be related to be cancers [11].

9-HYDROXY-6-(3-HYDROXYPROPYL)-4-(2-METHOXYPHENYL)PYRROLO[3,4-C] CARBAZOLE-1,3(2H,6H)-DIONE (DB07006)

This compound was reported to target WEE1 [5] that was reported to be targeted to treat cancer [12].

Staurosporine (DB02010)

This compound was reported to target LCK, PIM1, ITK, SYK, MAPKAPK2, GSK3B, CSK, CDK2, PIK3CG, PDPK1, ZAP70 [1, 2]. LCK and PDPK1 were reported to be related to cancers in the above. PIM1 was reported to be a therapeutic target gene for cancers [13]. ITK was reported to be a therapeutic target gene for cancers [14]. SYK was reported to suppress malignant growth of human breast cancer cells. [15]. A functional copy-number variation in MAPKAPK2 predicts risk and prognosis of lung cancer [16]. Inhibition of GSK3 activity was reported to trigger an apoptotic response in pancreatic cancer cells through JNK-dependent mechanisms [17]. Overexpression of the CSK gene was reported to suppress tumor metastasis in vivo [18]. CDK was well-known therapeutic target gene by inhibition [19]. PIK3CG was reported to be downregulated by CpG hypermethylation in human colorectal carcinoma [20]. ZAP-70 expression was reported to identify a chronic lymphocytic leukemia subtype [21].

6-Hydroxy-Flavin-Adenine Dinucleotide (DB02654)

There were no reports that suggest tight relationship with cancers.

2-({5-CHLORO-2-[(2-METHOXY-4-MORPHOLIN-4-YLPHENYL)AMINO] PYRIMIDIN-4-YL}AMINO)-N -METHYLBENZAMIDE (DB07460)

This compound was reported to target PTK2 [5] and ALK [22]. Molecular characterization of the role of PTK2 amplification in oral squamous cell carcinoma was investigated [23].

4-(4-METHYLPIPERAZIN-1-YL)-N-[5-(2-THIENYLACETYL)-1, 5-DIHYDROPYRROLO[3,4-C]PYRAZOL-3-YL]BENZAMIDE (DB07186)

This compound was reported to target AURKA and PLK1 [5]. AURKA was reported to be related to breast cancer [24]. Inhibition of PLK1 was reported to suppress cancer cell [25].

Riboflavin Monophosphate (DB03247)

This compound was reported to target 55 genes in DrugBank. However, targeted human genes were limited and were RPS6KA4, HAO2, POR, PPCDC, SGK1 [5], BLVRB [1, 2], HAO1, PNPO, RFK, NOS1, DPYD, and DHODH [1, 2, 5]. Not all, but some of them were reported to be related to cancers. NF-κB induction of RPS6KA4 was reported to block p53 degradation in colon cancer cell [26]. POR, also termed as P450, was suggested to related to cancers [27]. Elevated SGK1 was reported to predict resistance of breast cancer cells to Akt inhibitors [28]. Loss of NOS1 expression in high-grade renal cell carcinoma was reported to be associated with a shift of no signalling [29]. Absence of large intragenic rearrangements in the DPYD gene was reported in a large cohort of colorectal cancer patients treated with 5-FU-based chemotherapy [30]. DHODH inhibition was proposed as a candidate of cancer treatment [31].

EGLN3 (with Fe)

2-[4-[[(S)-1-[[(S)-2-[[(Rs)-3,3,3-Trifluoro-1-Isopropyl-2-Oxopropyl]Aminocarbonyl] Pyrrolidin-1-Yl-]Carbonyl]-2-Methylpropyl] Aminocarbonyl]Benzoylamino]Acetic Acid (DB03702)

This compound was reported to target CELA1 [1, 2, 5] that was reported to be upregulated in pancreatic tumor cell [32].

PYRIMIDINE-4,6-DICARBOXYLIC ACID BIS-[(PYRIDIN-3-YLMETHYL)-AMIDE] (DB04761)

This compound was reported to target MMP13 [5] that was reported to promote tumor angiogenesis directly and indirectly [33].

N-[(1-CHLORO-4-HYDROXYISOQUINOLIN-3-YL)CARBONYL]GLYCINE (DB08687)

This compound was reported to inhibit EGLN1 [34] that was reported to be differentially expressed in cancer-associated fibroblasts [35]. This compound was also reported to inhibit PHD2 [36] that was reported to improve tumor response to chemotherapy and to prevent side-toxicity when targeted [37].

2-{4-[2-(2-AMINO-4-OXO-4,7-DIHYDRO-3H-PYRROLO[2,3-D]PYRIMIDIN-5-YL)-ETHYL]-BENZOYLAMINO}-3-METHYL-BUTYRIC ACID (DB08131)

This compound was reported to target thyA [5] that was reported to be a critical target for cancer chemotherapy [38].

5-Formyl-6-Hydrofolic Acid (DB02718)

There were no reports that suggest tight relationship with cancers.

Dihydrofolic Acid (DB02015)

There were no reports that suggest tight relationship with cancers.

(6s)-5,6,7,8-Tetrahydrofolate (DB02031)

This compound was reported to target nos and thyA [1, 2]. Although these two are bacterial proteins, NOS1, which is human homolog to nos1, and thyA were reported to be related to cancers in the above.

PYRIMIDINE-4,6-DICARBOXYLIC ACID BIS-(4-FLUORO-3-METHYL-BENZYLAMIDE) (DB04760)

This compound was reported to target MMP13 [5] that was reported to be related to cancers in the above.

PYRIMIDINE-4,6-DICARBOXYLIC ACID BIS-(3-METHYL-BENZYLAMIDE) (DB04759)

This compound was reported to inhbit MMP13 [39] that was reported to be related to cancers in the above.

N-[(4-HYDROXY-8-IODOISOQUINOLIN-3-YL)CARBONYL]GLYCINE (DB07112)

This compound was reported to target EGLN1 [5] and to inhibit PHD2 [40]. EGNL1 and PHD2 were reported to be related to cancers in the above.

EGLN3 (without Fe)

N-[(1-CHLORO-4-HYDROXYISOQUINOLIN-3-YL)CARBONYL]GLYCINE (DB08687)

This compound was reported to inhibit EGLN1 [34], PHD2 [36] and HIF1A [5]. EGLN1 and PHD2 were reported to be related to cancers in the above. HIF1 was known to be target for cancer therapy [41].

2-[4-[[(S)-1-[[(S)-2-[[(Rs)-3,3,3-Trifluoro-1-Isopropyl-2-Oxopropyl]Aminocarbonyl] Pyrrolidin-1-Yl-]Carbonyl]-2-Methylpropyl] Aminocarbonyl]Benzoylamino]Acetic Acid (DB03702)

This compound was reported in the above.

PYRIMIDINE-4,6-DICARBOXYLIC ACID BIS-(3-METHYL-BENZYLAMIDE) (DB04759)

This compound was reported in the above.

5,10-Dideazatetrahydrofolic Acid (DB03625)

This compound was reported to inhibit mouse recombinant GARFTase [42]. Some compounds that inhibit GARFTase were proposed to be therapeutic targeting malignant mesothelioma [43].

PYRIMIDINE-4,6-DICARBOXYLIC ACID BIS-(4-FLUORO-3-METHYL-BENZYLAMIDE) (DB04760)

This compound was reported in the above.

N-[(4-HYDROXY-8-IODOISOQUINOLIN-3-YL)CARBONYL]GLYCINE (DB07112)

This compound was reported in the above.

Dihydrofolic Acid (DB02015)

This compound was reported in the above.

10-Propargyl-5,8-Dideazafolic Acid (DB03541)

This compound was reported to inhibit TYMS [44] and DHFR [45]. TYMS [46] and DHFR [47] were reported to be related to cancers, respectively.

Folic Acid (DB00158)

Folic acid were not proven to be related to cancers.

PYRIMIDINE-4,6-DICARBOXYLIC ACID BIS-[(PYRIDIN-3-YLMETHYL)-AMIDE] (DB04761)

This compound was reported in the above.

NUAK1

1-cyclobutyl-3-(3,4-dimethoxyphenyl)-1H-pyrazolo[3,4-d]pyrimidin-4-amine (DB08053)

This compound was reported to inhibit numerous target genes (more than a hundred reported in ChEMBL under the chemblid:CHEMBL1233881). For example, this compound was reported to inhibit CSF1R that was reported to improve the efficacy of radiotherapy in prostate Cancer if its signaling is blocked [48]. For more genes inhibited by this compound, see ChEMBL directly.

1-cyclopentyl-3-(1H-pyrrolo[2,3-b]pyridin-5-yl)-1H-pyrazolo[3,4-d]pyrimidin-4-amine (DB08052)

This compound was reported to inhibit numerous target genes (more than a hundred reported in ChEMBL under the chemblid:CHEMBL1081312). For example, this compound was also reported to inhibit CSF1R. For more genes inhibited by this compound, see ChEMBL directly.

1-(1-methylethyl)-3-quinolin-6-yl -1H-pyrazolo[3,4-d]pyrimidin-4-amine (DB08054)

This compound was reported to inhibit numerous target genes (more than a hundred reported in ChEMBL under the chemblid:CHEMBL1233882). For example, this compound was also reported to inhibit CSF1R. For more genes inhibited by this compound, see ChEMBL directly.

1-{7-cyclohexyl-6-[4-(4-methylpiperazin-1-yl)benzyl]-7H-pyrrolo[2,3-d]pyrimidin-2-yl}methanamine (DB07563)

This compound was reported to target Cathepsin K (CTSK) [5] that was reported to be expressed in prostate cancer progression [49].

1-TERT-BUTYL-3-(2,5-DIMETHYLBENZYL)-1H-PYRAZOLO[3,4-D]PYRIMIDIN-4-AMINE (DB08035)

This compound was reported to target AR [50] that was reported to promote prostate cancer epithelial cell growth and invasion in human prostate cancer-associated fibroblasts [51].

3-(4-Amino-1-Tert-Butyl-1h-Pyrazolo[3,4-D]Pyrimidin-3-Yl)Phenol (DB04463)

This compound was reported to target Carbonyl reductase 1 (CBR1) [5] that was reported to promote malignant behaviours when its expression decrease [52].

1-methyl-3-naphthalen-2-yl-1H-pyrazolo[3,4-d]pyrimidin-4-amine (DB08300)

This compound was reported to inhibit numerous target genes (more than a hundred reported in ChEMBL under the chemblid:CHEMBL1234815). For example, this compound was also reported to inhibit CSF1R. For more genes inhibited by this compound, see ChEMBL directly.

1-Ter-Butyl-3-P-Tolyl-1h-Pyrazolo[3,4-D]Pyrimidin-4-Ylamine (DB01809)

This compound was reported to inhibit numerous target genes (more than half a hundred reported in ChEMBL under the chemblid:CHEMBL306380). For example, this compound was reported to inhibit PKD1 that was reported to inhibit cancer cells migration and invasion via Wnt signaling pathway in vitro [53]. For more genes inhibited by this compound, see ChEMBL directly.

3-[(4-AMINO-1-TERT-BUTYL-1H-PYRAZOLO[3,4-D]PYRIMIDIN-3-YL)METHYL]PHENOL (DB08461)

This compound was reported to target AR [50] that was reported in the above.

1-tert-butyl-3-(3-methylbenzyl)-1H-pyrazolo[3,4-d]pyrimidin-4-amine (DB08699)

This compound was reported to target CAMK2G [5] that was reporter to be differentially expressed between colon and rectal cancer [54].

References

1. Overington JP, Al-Lazikani B, Hopkins AL: **How many drug targets are there?** *Nat Rev Drug Discov* 2006, **5**(12):993–996.
2. Imming P, Sinning C, Meyer A: **Drugs, their targets and the nature and number of drug targets**. *Nat Rev Drug Discov* 2006, **5**(10):821–834.
3. Raimondi C, Falasca M: **Targeting PDK1 in cancer**. *Curr. Med. Chem.* 2011, **18**(18):2763–2769.
4. Cui JJ, Tran-Dube M, Shen H, Nambu M, Kung PP, Pairish M, Jia L, Meng J, Funk L, Botrous I, McTigue M, Grodsky N, Ryan K, Padrique E, Alton G, Timofeevski S, Yamazaki S, Li Q, Zou H, Christensen J, Mroczkowski B, Bender S, Kania RS, Edwards MP: **Structure based drug design of crizotinib (PF-02341066), a potent and selective dual inhibitor of mesenchymal-epithelial transition factor (c-MET) kinase and anaplastic lymphoma kinase (ALK)**. *J. Med. Chem.* 2011, **54**(18):6342–6363.
5. Berman HM, Westbrook J, Feng Z, Gilliland G, Bhat TN, Weissig H, Shindyalov IN, Bourne PE: **The Protein Data Bank**. *Nucleic Acids Res.* 2000, **28**:235–242.
6. Sierra JR, Tsao MS: **c-MET as a potential therapeutic target and biomarker in cancer**. *Ther Adv Med Oncol* 2011, **3**(1 Suppl):21–35.
7. Harashima N, Tanaka K, Sasatomi T, Shimizu K, Miyagi Y, Yamada A, Tamura M, Yamana H, Itoh K, Shichijo S: **Recognition of the Lck tyrosine kinase as a tumor antigen by cytotoxic T lymphocytes of cancer patients with distant metastases**. *Eur. J. Immunol.* 2001, **31**(2):323–332.
8. Wang T, Yu D, Lamb ML: **Trk kinase inhibitors as new treatments for cancer and pain**. *Expert Opin Ther Pat* 2009, **19**(3):305–319.
9. Mazzieri R, Pucci F, Moi D, Zonari E, Ranghetti A, Berti A, Politi LS, Gentner B, Brown JL, Naldini L, De Palma M: **Targeting the ANG2/TIE2 axis inhibits tumor growth and metastasis by impairing angiogenesis and disabling rebounds of proangiogenic myeloid cells**. *Cancer Cell* 2011, **19**(4):512–526.
10. Srinivasan D, Plattner R: **Activation of Abl tyrosine kinases promotes invasion of aggressive breast cancer cells**. *Cancer Res.* 2006, **66**(11):5648–5655.
11. Katoh M: **Cancer genomics and genetics of FGFR2 (Review)**. *Int. J. Oncol.* 2008, **33**(2):233–237.
12. Stathis A, Oza A: **Targeting Wee1-like protein kinase to treat cancer**. *Drug News Perspect.* 2010, **23**(7):425–429.
13. Magnuson NS, Wang Z, Ding G, Reeves R: **Why target PIM1 for cancer diagnosis and treatment?** *Future Oncol* 2010, **6**(9):1461–1478.
14. Vargas L, Hamasy A, Nore BF, E Smith CI: **Inhibitors of BTK and ITK: State of the New Drugs for Cancer, Autoimmunity and Inflammatory Diseases**. *Scand. J. Immunol.* 2013, **78**(2):130–139.
15. Coopman PJ, Do MT, Barth M, Bowden ET, Hayes AJ, Basyuk E, Blancato JK, Vezza PR, McLeskey SW, Mangeat PH, Mueller SC: **The Syk tyrosine kinase suppresses malignant growth of human breast cancer cells**. *Nature* 2000, **406**(6797):742–747.
16. Liu B, Yang L, Huang B, Cheng M, Wang H, Li Y, Huang D, Zheng J, Li Q, Zhang X, Ji W, Zhou Y, Lu J: **A functional copy-number variation in MAPKAPK2 predicts risk and prognosis of lung cancer**. *Am. J. Hum. Genet.* 2012, **91**(2):384–390.
17. Marchand B, Tremblay I, Cagnol S, Boucher MJ: **Inhibition of glycogen synthase kinase-3 activity triggers an apoptotic response in pancreatic cancer cells through JNK-dependent mechanisms**. *Carcinogenesis* 2012, **33**(3):529–537.
18. Nakagawa T, Tanaka S, Suzuki H, Takayanagi H, Miyazaki T, Nakamura K, Tsuruo T: **Overexpression of the csk gene suppresses tumor metastasis in vivo**. *Int. J. Cancer* 2000, **88**(3):384–391.
19. Malumbres M, Pevarello P, Barbacid M, Bischoff JR: **CDK inhibitors in cancer therapy: what is next?** *Trends Pharmacol. Sci.* 2008, **29**:16–21.
20. Semba S, Itoh N, Ito M, Youssef EM, Harada M, Moriya T, Kimura W, Yamakawa M: **Down-regulation of PIK3CG, a catalytic subunit of phosphatidylinositol 3-OH kinase, by CpG hypermethylation in human colorectal carcinoma**. *Clin. Cancer Res.* 2002, **8**(12):3824–3831.
21. Wiestner A, Rosenwald A, Barry TS, Wright G, Davis RE, Henrickson SE, Zhao H, Ibbotson RE, Orchard JA, Davis Z, Stetler-Stevenson M, Raffeld M, Arthur DC, Marti GE, Wilson WH, Hamblin TJ, Oscier DG, Staudt LM: **ZAP-70 expression identifies a chronic lymphocytic leukemia subtype with unmutated immunoglobulin genes, inferior clinical outcome, and distinct gene expression profile**. *Blood* 2003, **101**(12):4944–4951.
22. Mesaros EF, Burke JP, Parrish JD, Dugan BJ, Anzalone AV, Angeles TS, Albom MS, Aimone LD, Quail MR, Wan W, Lu L, Huang Z, Ator MA, Ruggeri BA, Cheng M, Ott GR, Dorsey BD: **Novel 2,3,4,5-tetrahydro-benzo[d]azepine derivatives of 2,4-diaminopyrimidine, selective and orally bioavailable ALK inhibitors with antitumor efficacy in ALCL mouse models**. *Bioorg. Med. Chem. Lett.* 2011, **21**:463–466.
23. Quesnelle KM, Sparano AM, Wang Y, Weinstein GS, Brose MS: **Molecular characterization of the role of PTK2/FAK amplification in oral squamous cell carcinoma (OSCC)**. *AACR Meeting Abstracts* 2006, **2006**:798, [http://www.aacrmeetingabstracts.org/cgi/content/abstract/2006/1/798].
24. Bodvarsdottir SK, Vidarsdottir L, Eyfjord JE: **AURKA and breast cancer in BRCA1/2 mutation carriers**. *Cancer Epidemiol. Biomarkers Prev.* 2007, **16**(11):2517.
25. Liu X, Lei M, Erikson RL: **Normal cells, but not cancer cells, survive severe Plk1 depletion**. *Mol. Cell. Biol.* 2006, **26**(6):2093–2108.
26. Yoo TH, Lee JH, Chun HS, Chi SG: **Î ±-Lipoic acid prevents p53 degradation in colon cancer cells by blocking NF-Î°B induction of RPS6KA4**. *Anticancer Drugs* 2013, **24**(6):555–565.
27. Rodriguez-Antona C, Ingelman-Sundberg M: **Cytochrome P450 pharmacogenetics and cancer**. *Oncogene* 2006, **25**(11):1679–1691.

28. Sommer EM, Dry H, Cross D, Guichard S, Davies BR, Alessi DR: **Elevated SGK1 predicts resistance of breast cancer cells to Akt inhibitors**. *Biochem. J.* 2013, **452**(3):499–508.

29. Renaudin K, Denis MG, Karam G, Vallette G, Buzelin F, Laboisse CL, Jarry A: **Loss of NOS1 expression in high-grade renal cell carcinoma associated with a shift of NO signalling**. *Br. J. Cancer* 2004, **90**(12):2364–2369.

30. Pare L, Paez D, Salazar J, Del Rio E, Tizzano E, Marcuello E, Baiget M: **Absence of large intragenic rearrangements in the DPYD gene in a large cohort of colorectal cancer patients treated with 5-FU-based chemotherapy**. *Br J Clin Pharmacol* 2010, **70**(2):268–272.

31. Munier-Lehmann H, Vidalain PO, Tangy F, Janin YL: **On dihydroorotate dehydrogenases and their inhibitors and uses**. *J. Med. Chem.* 2013, **56**(8):3148–3167.

32. Zenzmaier C, Hermann M, Hengster P, Berger P: **Dickkopf-3 maintains the PANC-1 human pancreatic tumor cells in a dedifferentiated state**. *Int. J. Oncol.* 2012, **40**:40–46.

33. Kudo Y, Iizuka S, Yoshida M, Tsunematsu T, Kondo T, Subarnbhesaj A, Deraz EM, Siriwardena SB, Tahara H, Ishimaru N, Ogawa I, Takata T: **Matrix metalloproteinase-13 (MMP-13) directly and indirectly promotes tumor angiogenesis**. *J. Biol. Chem.* 2012, **287**(46):38716–38728.

34. Warshakoon NC, Wu S, Boyer A, Kawamoto R, Renock S, Xu K, Pokross M, Evdokimov AG, Zhou S, Winter C, Walter R, Mekel M: **Design and synthesis of a series of novel pyrazolopyridines as HIF-1alpha prolyl hydroxylase inhibitors**. *Bioorg. Med. Chem. Lett.* 2006, **16**(21):5687–5690.

35. Bozoky B, Savchenko A, Csermely P, Korcsmaros T, Dul Z, Ponten F, Szekely L, Klein G: **Novel signatures of cancer-associated fibroblasts**. *Int. J. Cancer* 2013, **133**(2):286–293.

36. Leung IK, Flashman E, Yeoh KK, Schofield CJ, Claridge TD: **Using NMR solvent water relaxation to investigate metalloenzyme-ligand binding interactions**. *J. Med. Chem.* 2010, **53**(2):867–875.

37. Leite de Oliveira R, Deschoemaeker S, Henze AT, Debackere K, Finisguerra V, Takeda Y, Roncal C, Dettori D, Tack E, Jonsson Y, Veschini L, Peeters A, Anisimov A, Hofmann M, Alitalo K, Baes M, D'hooge J, Carmeliet P, Mazzone M: **Gene-targeting of Phd2 improves tumor response to chemotherapy and prevents side-toxicity**. *Cancer Cell* 2012, **22**(2):263–277.

38. Rose MG, Farrell MP, Schmitz JC: **Thymidylate synthase: a critical target for cancer chemotherapy**. *Clin Colorectal Cancer* 2002, **1**(4):220–229.

39. Carrascal N, Rizzo RC: **Calculation of binding free energies for non-zinc chelating pyrimidine dicarboxamide inhibitors with MMP-13**. *Bioorg. Med. Chem. Lett.* 2009, **19**:47–50.

40. Frohn M, Viswanadhan V, Pickrell AJ, Golden JE, Muller KM, Burli RW, Biddlecome G, Yoder SC, Rogers N, Dao JH, Hungate R, Allen JR: **Structure-guided design of substituted aza-benzimidazoles as potent hypoxia inducible factor-1alpha prolyl hydroxylase-2 inhibitors**. *Bioorg. Med. Chem. Lett.* 2008, **18**(18):5023–5026.

41. Semenza GL: **Targeting HIF-1 for cancer therapy**. *Nat. Rev. Cancer* 2003, **3**(10):721–732.

42. Deng Y, Zhou X, Kugel Desmoulin S, Wu J, Cherian C, Hou Z, Matherly LH, Gangjee A: **Synthesis and biological activity of a novel series of 6-substituted thieno[2,3-d]pyrimidine antifolate inhibitors of purine biosynthesis with selectivity for high affinity folate receptors over the reduced folate carrier and proton-coupled folate transporter for cellular entry**. *J. Med. Chem.* 2009, **52**(9):2940–2951.

43. Cherian C, Kugel Desmoulin S, Wang L, Polin L, White K, Kushner J, Stout M, Hou Z, Gangjee A, Matherly LH: **Therapeutic targeting malignant mesothelioma with a novel 6-substituted pyrrolo[2,3-d]pyrimidine thienoyl antifolate via its selective uptake by the proton-coupled folate transporter**. *Cancer Chemother. Pharmacol.* 2013, **71**(4):999–1011.

44. Gangjee A, Li W, Kisliuk RL, Cody V, Pace J, Piraino J, Makin J: **Design, synthesis, and X-ray crystal structure of classical and nonclassical 2-amino-4-oxo-5-substituted-6-ethylthieno[2,3-d]pyrimidines as dual thymidylate synthase and dihydrofolate reductase inhibitors and as potential antitumor agents**. *J. Med. Chem.* 2009, **52**(15):4892–4902.

45. Gangjee A, Lin X, Kisliuk RL, McGuire JJ: **Synthesis of N-4-[(2,4-diamino-5-methyl-4,7-dihydro-3H-pyrrolo[2,3-d]pyrimidin-6-yl)thio]benzoyl-L-glutamic acid and N-4-[(2-amino-4-oxo-5-methyl-4,7-dihydro-3H-pyrrolo[2,3-d]pyrimidin-6-yl)thio]benzoyl-L-glutamic acid as dual inhibitors of dihydrofolate reductase and thymidylate synthase and as potential antitumor agents**. *J. Med. Chem.* 2005, **48**(23):7215–7222.

46. Cho YB, Chung HJ, Lee WY, Choi SH, Kim HC, Yun SH, Chun HK: **Relationship between TYMS and ERCC1 mRNA expression and in vitro chemosensitivity in colorectal cancer**. *Anticancer Res.* 2011, **31**(11):3843–3849.

47. Morales C, Garcia MJ, Ribas M, Miro R, Munoz M, Caldas C, Peinado MA: **Dihydrofolate reductase amplification and sensitization to methotrexate of methotrexate-resistant colon cancer cells**. *Mol. Cancer Ther.* 2009, **8**(2):424–432.

48. Xu J, Escamilla J, Mok S, David J, Priceman S, West B, Bollag G, McBride W, Wu L: **CSF1R signaling blockade stanches tumor-infiltrating myeloid cells and improves the efficacy of radiotherapy in prostate cancer**. *Cancer Res.* 2013, **73**(9):2782–2794.

49. Brubaker KD, Vessella RL, True LD, Thomas R, Corey E: **Cathepsin K mRNA and protein expression in prostate cancer progression**. *J. Bone Miner. Res.* 2003, **18**(2):222–230.

50. Estebanez-Perpina E, Arnold LA, Arnold AA, Nguyen P, Rodrigues ED, Mar E, Bateman R, Pallai P, Shokat

KM, Baxter JD, Guy RK, Webb P, Fletterick RJ: **A surface on the androgen receptor that allosterically regulates coactivator binding**. *Proc. Natl. Acad. Sci. U.S.A.* 2007, **104**(41):16074–16079.

51. Yu S, Xia S, Yang D, Wang K, Yeh S, Gao Z, Chang C: **Androgen receptor in human prostate cancer-associated fibroblasts promotes prostate cancer epithelial cell growth and invasion**. *Med. Oncol.* 2013, **30**(3):674.

52. Murakami A, Yakabe K, Yoshidomi K, Sueoka K, Nawata S, Yokoyama Y, Tsuchida S, Al-Mulla F, Sugino N: **Decreased carbonyl reductase 1 expression promotes malignant behaviours by induction of epithelial mesenchymal transition and its clinical significance**. *Cancer Lett.* 2012, **323**:69–76.

53. Zhang K, Ye C, Zhou Q, Zheng R, Lv X, Chen Y, Hu Z, Guo H, Zhang Z, Wang Y, Tan R, Liu Y: **PKD1 inhibits cancer cells migration and invasion via Wnt signaling pathway in vitro**. *Cell Biochem. Funct.* 2007, **25**(6):767–774.

54. Li JN, Zhao L, Wu J, Wu B, Yang H, Zhang HH, Qian JM: **Differences in gene expression profiles and carcinogenesis pathways between colon and rectal cancer**. *J Dig Dis* 2012, **13**:24–32.
